# Two classes of amine/glutamate multi-transmitter neurons innervate Drosophila internal male reproductive organs

**DOI:** 10.1101/2025.07.23.666348

**Published:** 2025-07-28

**Authors:** Martha Chaverra, John Paul Toney, Lizetta D. Dardenne-Ankringa, Jace Tolleson Knee, Ann R. Morris, Joseph B. Wadhams, Sarah J. Certel, R. Steven Stowers

**Affiliations:** 1 Montana State University, Department of Microbiology and Cell Biology, Bozeman, MT 59717; 2 The University of Montana, Division of Biological Sciences, Missoula, MT 59812

## Abstract

The essential outcome of a successful mating is the transfer of genetic material from males to females in sexually reproducing animals from insects to mammals. In males, this culminates in ejaculation, a precisely timed sequence of organ contractions driven by the concerted activity of interneurons, sensory neurons, and motor neurons. Although central command circuits that trigger copulation have been mapped, the motor architecture and the chemical logic that couple specific neuronal subclasses to organ specific contractility, seminal fluid secretion, and sperm emission remain largely uncharted. This gap in knowledge limits our ability to explain how neural circuits adapt to varying contexts and how their failure contributes to infertility. Here we present an in-depth anatomical and functional analysis of the motor neurons that innervate the internal male reproductive tract of Drosophila melanogaster. We identify two classes of multi-transmitter motor neurons based on neurotransmitter usage, namely octopamine and glutamate neurons (OGNs) and serotonin and glutamate neurons (SGNs), each with a biased pattern of innervation: SGNs predominate in the accessory glands, OGNs in the ejaculatory duct, with equal contributions of each to the seminal vesicles. Both classes co-express vesicular transporters for glutamate (vGlut) and amines (vMAT), confirming their dual chemical identity. Their target organs differentially express receptors for glutamate, octopamine, and serotonin, suggesting combinatorial neuromodulation of contractility. Functional manipulations show that SGNs are essential for male fertility but OGNs are dispensable. Glutamatergic transmission from both classes is also dispensable for fertility. These findings provide the first high-resolution map linking multi-transmitter motor neurons to specific reproductive organs, reveal an unexpected division of labor between serotonergic and octopaminergic signaling pathways, and establish a framework for dissecting conserved neural principles that govern ejaculation and male fertility.

## Introduction

Reproduction is a biological imperative of all species. For most sexually reproducing animal species, this involves locating a mate, courtship, and most essentially, the transfer of genetic material via copulation. The endpoint of copulation, ejaculation, consists of two coordinated phases, emission and expulsion (ALWAAL et al. 2015; SONI et al. 2022). During the emission phase, sperm and seminal fluid are relocated from their site of origin to a central canal for transfer to the female during the subsequent expulsion phase. The series of events that culminate in ejaculation are regulated by a spinal ejaculation generator located in the lumbar L3-L4 region of the spinal cord of mammals that executes a motor program coordinating a series of muscle contractions and relaxations of the internal and external male genitalia (TRUITT AND COOLEN 2002; CARRO-JUAREZ et al. 2003; CHEHENSSE et al. 2017).

While male external genitalia exhibit remarkable evolutionary variation (EBERHARD 1985), internal male reproductive organs exhibit remarkable evolutionary conservation over the last 600 million years since the last common ancestor of insects and mammals (GUERRERO et al. 2004; GOMES et al. 2012; BEGUELINI et al. 2013; TOMAS et al. 2019; FROMME et al. 2021; OLIVEIRA et al. 2021; YARTSEV AND EVSEEVA 2021; MACIEL et al. 2024). These evolutionarily conserved structures of the male reproductive system include paired testes, seminal vesicles (SVs), and semen producing organs (prostate glands in humans, accessory glands (AGs) in insects), as well as a singular duct (urethra in humans, ejaculatory duct (ED) in insects) through which sperm and seminal fluid flow during mating.

Drosophila melanogaster has been instrumental in revealing fundamental principles of male reproductive behavior and physiology that extend beyond insects, including how neural circuits control mating drive, courtship, and the mechanics and duration of copulation (ACEBES et al. 2004; BILLETER et al. 2006a; VILLELLA AND HALL 2008; TAYLER et al. 2012; CRICKMORE AND VOSSHALL 2013; PAVLOU et al. 2016; JOIS et al. 2018; BAKER et al. 2024). Among the findings of these studies is the existence of neural circuitry in the ventral nerve cord that regulates ejaculation similar to the spinal ejaculation generator circuitry of mammals, suggesting an ancient evolutionary origin. How the motor neurons directly innervating the internal male reproductive organs control the events leading to ejaculation is less well understood. It is also known in Drosophila and other species, including humans, that male ejaculate quality is plastic and can be adapted to environmental conditions, especially the level of male-male competition (GAGE AND BAKER 1991; WEDELL AND COOK 1999; PIZZARI et al. 2003; POUND AND GAGE 2004; BRETMAN et al. 2009; GARBACZEWSKA et al. 2013; HOPKINS et al. 2019; DELECCE et al. 2025), but the neural mechanisms underlying these adaptations remain to be identified.

Previous studies have determined there are at least three types of motor neurons innervating the Drosophila internal male reproductive system distinguished by neurotransmitter usage including serotonergic (LEE AND HALL 2001), octopaminergic (PAULS et al. 2018), and glutamatergic (PAVLOU et al. 2016) motor neurons, although there may be at least some overlap in neurotransmitter usage as serotonin/glutamate multi-transmitter motor neurons have been reported in the seminal vesicle (CASTELLANOS et al. 2013). How these neurons coordinate the motor program to elicit ejaculation is an open question, as is their anatomical organization relative to each other. Clarifying the targets and anatomy of these motor neuron classes will uncover critical principles for how neuronal coordination of internal male reproductive organ function contributes to male fertility. To address these questions, we performed a comprehensive analysis of the neurons innervating each internal organ, defined their neurotransmitter complements and receptor profiles, and tested their necessity for successful sperm transfer and fecundity.

## Results

### Drosophila male reproductive system anatomy

**Figure 1.**
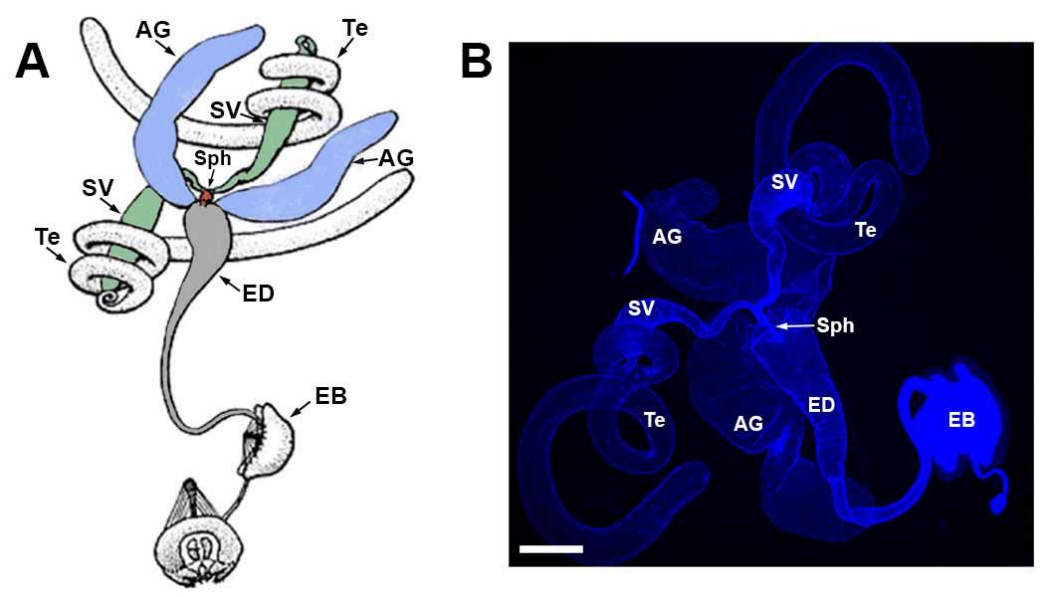
The Drosophila male reproductive system. A) schematic diagram showing paired testes, SVs (green), and AGs (purple), and a singular sphincter (red), ED (gray), and ejaculatory bulb. B) actual male reproductive system. Te-testes, SV-seminal vesicle, AG-accessory gland, Sph-sphincter, ED-ejaculatory duct, EB-ejaculatory bulb. Scale bar: 200μm.

The Drosophila male reproductive system consists of paired testes, SVs, and AGs, as well as a singular ED and ejaculatory bulb (Figure 1) (DEMEREC 1950). The testes are surrounded by smooth muscle with no neuronal innervation, while the SVs, AGs, and ED are ensheathed by neuronally innervated skeletal muscles (LEE AND HALL 2001; BILLETER et al. 2006b; SUSIC-JUNG et al. 2012). The ejaculatory bulb also possesses neuronally innervated skeletal muscle for pumping sperm out of the ED to the female. The SVs fuse just prior to termination in the ED where they form a narrow sphincter that opens during mating to allow sperm to flow. The AGs, insect equivalent of mammalian prostate glands, produce the bulk of seminal fluid (WOLFNER 1997; MAJANE et al. 2022) and remain separate before terminating in the ED (BAIRATI 1968). Underlying the muscles of the internal male reproductive system organs are a layer of secretory epithelial cells (BAIRATI 1968) that are the cellular source of the seminal fluid components (SEPIL et al. 2019; WIGBY et al. 2020).

### Neurotransmitter identity of neurons innervating internal male reproductive organs

**Figure 2.**
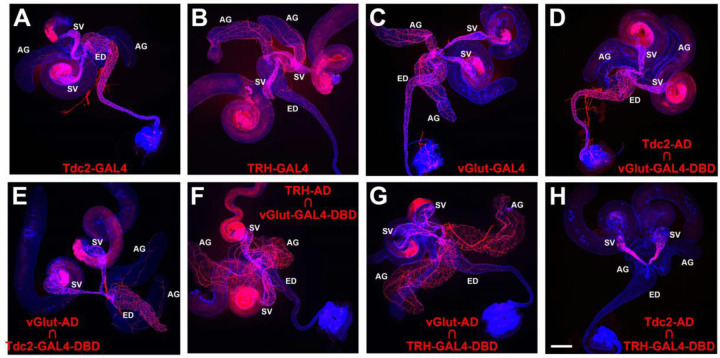
Gal4 and Split-GAL4 expression patterns in the Drosophila male reproductive system. A) *Tdc2-GAL4*; B) *TRH-GAL4*; C) vGlut-GAL4; D) Tdc2-AD ∩ *vGlut-GAL4-DBD*; E) *vGlut-AD* ∩ *Tdc2-GAL4-DBD*; F) *TRH-AD* ∩ *vGlut-GAL4-DBD*; G) *vGlut-AD* ∩ *TRH-GAL4-DBD*; H) *Tdc2-AD* ∩ *TRH-GAL4-DBD*. Scale bar: 200μm.

To confirm previous reports of innervation of the Drosophila male reproductive system by octopaminergic, serotonergic, and glutamatergic neurons, GAL4 drivers for the octopamine (OA) neurotransmitter synthesis enzyme tyrosine decarboxylase 2 (Tdc2), the serotonin (5-Hydroxytryptamine, 5-HT) neurotransmitter synthesis enzyme tryptophan hydroxylase (TRH) and the vesicular glutamate transporter (vGlut) were used to drive expression of a *UAS-CD8-mCherry* plasma membrane reporter. As expected, all three drivers exhibited expression in neurons of the male reproductive system, but with distinguishable differences in expression patterns. *Tdc2-GAL4* exhibited dense innervation of the SV and ED with sparse innervation of the AG (Figure 2, A). *TRH-GAL4* exhibited dense innervation of the SV and AGs, with modest innervation of the ED (Figure 2, B), and *vGlut-GAL4* exhibited dense innervation of all three organs (Figure 2, C).

### OA/glutamate neurons are distinct from 5-HT/glutamate neurons

To determine if the observed neuronal expression of the GAL4 drivers for Tdc2, TRH, and vGlut are coincident or exclusive, two separate approaches were implemented. In the first approach, split-GAL4 drivers specific for each neurotransmitter were tested in pairwise combinations. Both *Tdc2-AD* paired with *vGlut-GAL4-DBD* (Figure 2, D) and *vGlut-AD* paired with *Tdc2-GAL4-DBD* (Figure 2, E) exhibited expression patterns very similar to *Tdc2-GAL4* with dense innervation of the SV and ED, and sparse innervation of the AGs. These results suggest that all the Tdc2 neurons innervating the male reproductive system are OA/glutamate dual neurotransmitter neurons (OGNs). Similarly, both *TRH-AD* paired with *vGlut-GAL4-DBD* (Figure 2, F) and *vGlut-AD* paired with *TRH-GAL4-DBD* (Figure 2, G) exhibited expression patterns very similar to *TRH-GAL4* with dense innervation of the SVs and AGs with modest innervation of the ED. These results suggest that all the serotonergic neurons innervating the male reproductive system are 5-HT/glutamate dual neurotransmitter neurons (SGNs). To determine if there is overlap between the OGNs and SGNs, *Tdc2-AD* was paired with *TRH-GAL4-DBD* with the result that no neuronal expression was observed (Figure 2, H). This result suggests the OGNs and SGNs are exclusive and non-overlapping.

A second approach to assess overlap between octopaminergic, serotonergic, and glutamatergic neurons of the male reproductive system utilized pairwise combinations of GAL4 and LexA drivers specific for each neurotransmitter. Pairing *TRH-GAL4* and *Tdc2-LexA* together with *UAS-6XGFP* and *LexAop-6XmCherry* reporters revealed no overlap in either the SVs (Figure 3, A-F), ED (Figure 3, G-L), or AGs (Figure 3, M-R). These results are consistent with the split-GAL4 result above of no intersection between octopaminergic and serotonergic neurons of the male reproductive system, although both neuron types are often tightly fasciculated, especially in the SVs. Pairing *vGlut-LexA* with *TRH-GAL4* revealed a high degree of overlap in the SVs (Figures 3S1, A-F), partial overlap in the ED (Figures 3S1, G-L), and near complete overlap in the AGs (Figures 3S1, M-R). Pairing *vGlut-LexA* with *Tdc2-GAL4* revealed significant overlap in the SVs (Figures 3S2, A-F), majority overlap in the ED (Figures 3S2, G-L), and limited overlap in the AGs (Figures 3S2, M-R). The results of the split-GAL4 intersectional strategy and the GAL4/LexA overlap strategy are entirely consistent with each other. Taken together, they demonstrate that the Drosophila internal male reproductive system receives input from two distinct and nonoverlapping motor-neuron populations, one population that co-expresses octopamine and glutamate (OGNs) and another distinct population that co-expresses serotonin and glutamate (SGNs). The SV receives roughly equal innervation from both classes, the AG is innervated more extensively by SGNs, and the ED is innervated more extensively by OGNs.

### OGNs are *fru* +/*dsx* + while SGNs are *fru*+/*dsx* −

Neurons that innervate the male reproductive tract are expected to be sexually dimorphic (BILLETER AND GOODWIN 2004; YAMAMOTO 2007; SIWICKI AND KRAVITZ 2009; VERHULST AND VAN DE ZANDE 2015). To determine whether they express the canonical sex-determination genes *fruitless* (*fru*) and/or *doublesex* (*dsx*), *fru-GAL4* and *vGlut-LexA* were paired with *UAS-6XGFP* and *LexAop-6XmCherry*. This experiment revealed complete overlap: every vGlut-positive neuron in the reproductive system—both OGNs and SGNs—was also *fru*-positive (Fig. 3S3, A–C). To examine *dsx* expression, the octopaminergic driver *Tdc2-AD* was combined with *dsx-GAL4-DBD*. The resulting pattern (dense terminals in the SVs and ED, sparse arbors in the AGs; Fig. 3S3, D) matched that of *Tdc2-GAL4* alone, indicating that all OGNs are *dsx*-positive. In contrast, pairing the serotonergic *TRH-AD* with *dsx-GAL4-DBD* produced no signal in the reproductive tract (Fig. 3S3, E), showing that SGNs are *dsx*-negative. Together, these experiments establish that OGNs are *fru*+/*dsx*+, whereas SGNs are *fru*+/*dsx*−, underscoring a molecular distinction between the two neuromodulatory inputs to the male reproductive system.

### Non-overlapping expression of TβH and Tdc2 with 5-HT confirms exclusivity of OGNs and SGNs

**Figure 4.**
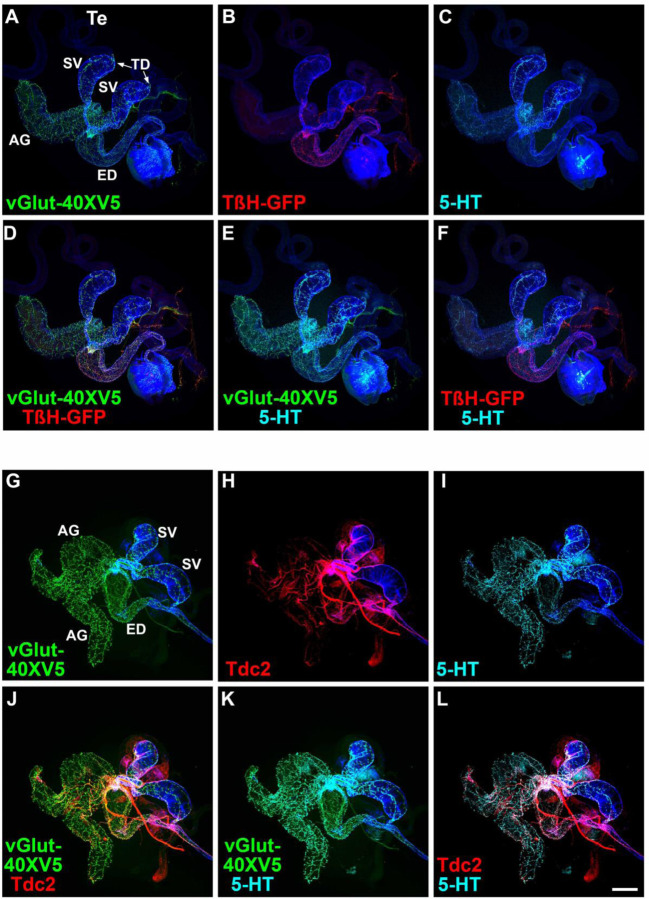
Expression of vGlut, TβH-GFP, Tdc2, and 5-HT in the Drosophila male reproductive system. A) vGlut-40XV5; B) TβH-GFP; C) 5-HT; D) vGlut-40XV5, TβH-GFP overlay; E) vGlut-40XV5, 5-HT overlay; F) TβH-GFP, 5-HT overlay. G) vGlut-40XV5; H) Tdc2; I) 5-HT; J) vGlut-40XV5, Tdc2 overlay; K) vGlut-40XV5, 5-HT overlay; L) Tdc2, 5-HT overlay. Scale bar: 200μm.

Several predictions follow from the finding that non-overlapping sets of OGNs and SGNs innervate the male reproductive system. Among these are that the neurotransmitter synthesis enzymes for OA, Tyramine-β-hydroxylase (TβH), and Tyrosine decarboxylase 2 (Tdc2) should be exclusively expressed in the OGNs along with the sole Drosophila vesicular glutamate transporter (vGlut), while 5-HT should be exclusively expressed in the SGNs along with vGlut-40XV5. To test these predictions, vGlut-40XV5 expression was separately assessed in combination with TβH or Tdc2. 5-HT expression, visualized with an anti-5-HT antibody, was also included in this analysis to distinguish SGNs. As predicted, extensive vGlut-40XV5 expression was observed throughout the male reproductive system except for the testes (Figures 4, A and G) with a sharp boundary of neuronal innervation at the testicular duct (arrows, Figure 4, A). Also as predicted, TβH-GFP and Tdc2 were observed in the male reproductive system with expression patterns similar to that of *Tdc2-GAL4* (Figures 4, B and H, respectively). Pairwise combinations of overlapping expression of vGlut-40XV5, 5-HT, and either TβH (Figures 4, D-F) or Tdc2 (Figures 4, J-L) are also shown.

Higher resolution images of vGlut-40XV5, TβH-GFP, Tdc2, and 5-HT in the male reproductive system allow an assessment of overlapping expression. In the SV, broad robust expression was observed for vGlut-40XV5 (Figures 4S1, A, G, M, and S) and 5-HT (Figures 4S1, C, I, O, and U), with somewhat more restricted expression for TβH-GFP (Figure 4S1, B and H) and Tdc2 (Figure 4S1, N, T). Pairwise overlapping expression is also shown for vGlut-40XV5 and 5-HT with TβH-GFP (Figures 4S1, D-F and J-L) and Tdc2 (Figures 4S1, P-R and V-X). In the highest resolution images, coincident expression is evident between vGlut-40XV5 with TβH-GFP (Figure 4S1, J) and vGlut-40XV5 with Tdc2 (Figure 4WS1, V). Consistent with results above, exclusivity of expression is apparent between TβH-GFP and 5-HT (Figure 4S1, L) and between Tdc2 and 5-HT (Figure 4S1, X), although the tight fasciculation of OGNs and SGNs is evident.

Similar results were obtained in higher resolution images of the ED and AGs although the extent of innervation of the different amines varies between these tissues with more OGNs than SGNs in the ED and the reciprocal in the AGs. In higher resolution images of the ED, strong extensive expression was observed for vGlut-40XV5 (Figures 4S2, A, G, M, and S) with moderately less expression for TβH-GFP (Figure 4S2, B and H) and Tdc2 (Figure 4S2, N, T), with significantly less expression of 5-HT (Figures 4S2, C, I, O, and U. Pairwise overlapping expression is also shown for vGlut-40XV5 and 5-HT with TβH-GFP (Figures 4S2, D-F and J-L) and Tdc2 (Figures 4S2, P-R and V-X). In the highest resolution images, coincident expression is evident between vGlut-40XV5 with TβH-GFP (Figure 4S2, J) and vGlut-40XV5 with Tdc2 (Figure 4S2, V). As in the SV, exclusivity of expression is apparent between TβH-GFP and 5-HT (Figure 4S2, L) and between Tdc2 and 5-HT (Figure 4S2, X), also with tight fasciculation of the OGNs and SGNs.

In higher resolution images of the AGs, robust widespread expression was observed for vGlut-40XV5 (Figures 4S3, A, G, M, and S) and 5-HT (Figures 4S3, C, I, O, and U, with no expression of TβH-GFP (Figure 4S3, B and H), and sparse expression of Tdc2 (Figure 4S3, N, T). Pairwise overlapping expression is also shown for vGlut-40XV5 and 5-HT with TβH-GFP (Figures 4S3, D-F and J-L) and Tdc2 (Figures 4S3, P-R and V-X). In the highest resolution images, coincident expression is evident between vGlut-40XV5 and Tdc2 (Figure 4S3, V). As in the SV and ED, exclusivity of expression is apparent between Tdc2 and 5-HT (Figure 4S3, X).

The expression of vGlut-40XV5, TβH-GFP, and 5-HT was also assessed at the junction of the SVs and AGs with the ED. vGlut-40XV5 innervation of both the SV and AG is particularly dense (Figure 4S4, A). Significant expression of TβH-GFP and 5-HT was also observed (Figure 4S4, B and C, respectively). Pairwise overlapping expression for vGlut-40XV5, TβH-GFP, and 5-HT is also shown (Figure 4S4, D-F). These experiments showing no overlap in expression of TβH-GFP and Tdc2 with 5-HT provide additional support to the exclusivity of OGNs and SGNs. Collectively, these findings confirm that octopaminergic vGlut-positive and serotonergic vGlut-positive motor neurons form distinct, nonoverlapping populations that differentially innervate the Drosophila male reproductive tract, underscoring strict transmitter exclusivity within this circuit.

### Expression of vGlut and vMAT in both OGNs and SGNs further confirms multi-neurotransmitter usage

**Figure 5.**
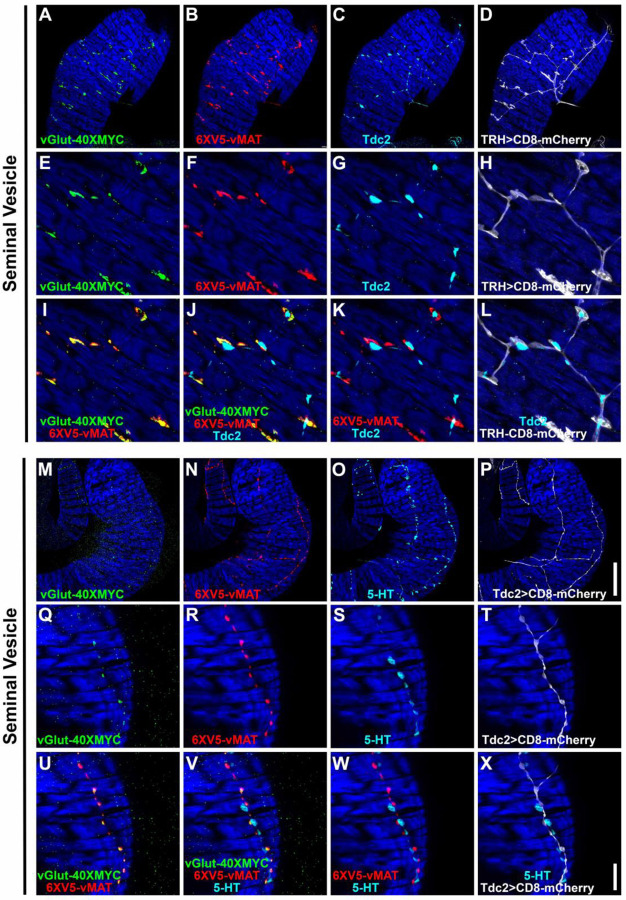
Co-conditional expression of vGlut-40XMYC and 6XV5-vMAT in the SV of serotonergic (TRH) or octopaminergic (Tdc2) neurons of the Drosophila male reproductive system. A-D) 63X. A) vGlut-40XMYC; B) 6XV5-vMAT; C) Tdc2; D) TRH>CD8-mCherry. E-L) 63X zoom 4X. E) vGlut-40XMYC; F) 6XV5-vMAT; G) Tdc2; H) TRH>CD8-mCherry; I) vGlut-40XMYC, 6XV5-vMAT overlay; J) vGlut-40XMYC, 6XV5-vMAT, Tdc2 overlay; K) 6XV5-vMAT, Tdc2 overlay; L) Tdc2, TRH>CD8-mCherry overlay. M-P) 63X. M) vGlut-40XMYC; N) 6XV5-vMAT; O) 5-HT; P) Tdc2>CD8-mCherry. Q-X) 63X zoom 4X. Q) vGlut-40XMYC; R) 6XV5-vMAT; S) 5-HT; T) Tdc2>CD8-mCherry; U) vGlut-40XMYC, 6XV5-vMAT overlay; V) vGlut-40XMYC, 6XV5-vMAT, 5-HT overlay; W) 6XV5-vMAT, 5-HT overlay; X) Tdc2, Tdc2>CD8-mCherry overlay. Scale bars: P-50μm; X-10μm.

**Figure 6.**
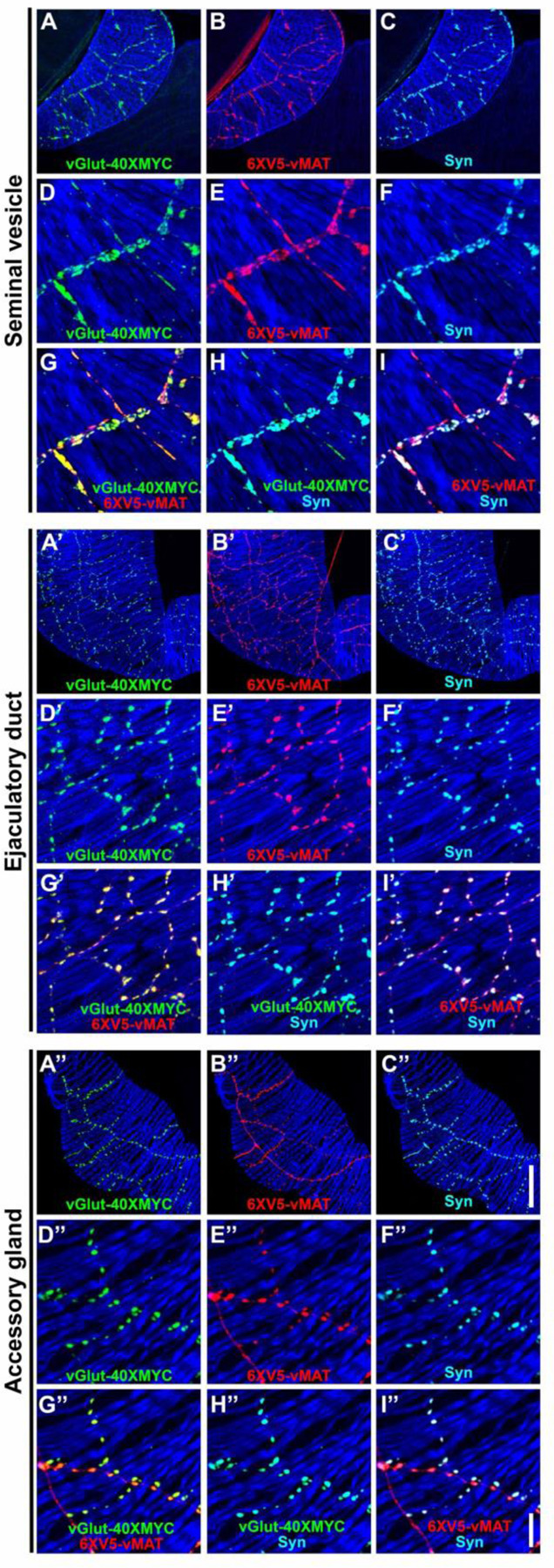
Expression of the synaptic vesicle marker Synapsin in combination with vGlut-40XMYC and 6XV5-vMAT in the SV, ED, and AG of the Drosophila male reproductive system. A-I) SV. A) vGlut-40XMYC; B) 6XV5-vMAT; C) Synapsin; D) vGlut-40XMYC; E) 6XV5-vMAT; F) Synapsin; G) vGlut-40XMYC, 6XV5-vMAT overlay; H) vGlut-40XMYC, Synapsin overlay; I) 6XV5-vMAT, Synapsin overlay. A’-I’) ED. A’) vGlut-40XMYC; B’) 6XV5-vMAT; C’) Synapsin; D’) vGlut-40XMYC; E’) 6XV5-vMAT; F’) Synapsin; G’) vGlut-40XMYC, 6XV5-vMAT overlay; H’) vGlut-40XMYC, Synapsin overlay; I’) 6XV5-vMAT, Synapsin overlay. A’’-I’’) AG. A’’) vGlut-40XMYC; B’’) 6XV5-vMAT; C’’) Synapsin; D’’) vGlut-40XMYC; E’’) 6XV5-vMAT; F’’) Synapsin; G’’) vGlut-40XMYC, 6XV5-vMAT overlay; H’’) vGlut-40XMYC, Synapsin overlay; I’’) 6XV5-vMAT, Synapsin overlay. Scale bars: C’’-50μm; I’’-10μm.

Co-labeling of vGlut-40XV5 with TβH / Tdc2 in OGNs and with 5-HT in SGNs implies both neuron types are multi-transmitter and predicts they will express not only vGlut but also the sole Drosophila monoamine vesicular transporter vMAT known to package both OA and 5-HT (DESHPANDE et al. 2020). Since OGNs and SGNs often fasciculate tightly together, co-conditional expression of vGlut and vMAT was implemented to visualize expression separately in each population. This was achieved using either the *TRH-GAL4* or *Tdc2-GAL4* drivers in combination with the *B2RT-STOP-B2RT-vGlut-40XMYC*, *RSRT-STOP-RSRT-6XV5-vMAT*, *UAS-B2* and *UAS-R* recombinase transgenes. Using the *TRH-GAL4* driver to restrict conditional expression to SGNs, as predicted, both vGlut-40XMYC (Figures 5, A, E) and 6XV5-vMAT (Figures 5, B, F) were detected in the SV. Tdc2 expression was also included in this experiment (Figures 5, C and G) as was a *UAS-CD8-mCherry* reporter to outline SGNs (Figures 5, D and H). A high degree of overlap was observed between vGlut-40XMYC and 6XV5-vMAT (Figure5, I) with no overlap between Tdc2 and the other markers (Figures 5, J-L).

In a reciprocal experiment in the SV using the *Tdc2-GAL4* driver and 5-HT immunostaining, both vGlut-40XMYC (Figure 5, M and Q) and 6XV5-vMAT (Figure 5, N and R) were detected with a mostly overlapping distribution (Figure 5, U). 5-HT immunostaining was in close proximity but did not overlap with any of the other markers (Figure 5, V-X). It is also notable that the morphology of the SGNs is distinct from the OGNs with the SGNs being larger and more extensively branched compared to the OGNs (SGNs-Figures 5, D and H; OGNs-Figures 5, P and T), suggesting SGNs may assert more regulatory control of SV muscle activity than OGNs.

Similar results were obtained in these co-conditional experiments for the ED and AGs. With the *TRH-GAL4* experiment, vGlut-40XMYC (Figure 5S1, A and E) and 6XV5-vMAT (Figure 5S1, B and F) were both present with a highly overlapping distribution (Figure 5S1, I). Tdc2 did not overlap with any of the other markers (Figure 5S1, J-L). With the *Tdc2-GAL4* experiment, vGlut-40XMYC (Figure 5S1, M and Q) and 6XV5-vMAT (Figure 5S1, N and R) were both observed in a highly overlapping distribution (Figures 5S1, U). 5-HT immunostaining did not overlap with any of the other markers (Figures 5S1, V-X). Note that morphological differences between the SGNs and OGNs in the ED are not as easily distinguished as in the SV.

With the *TRH-GAL4* experiment in the AGs, both vGlut-40XMYC (Figures 5S2, A and E) and 6XV5-vMAT (Figures 5S2, B and F) are present in a largely overlapping distribution (Figure 5S2, I). As with the SV and ED, Tdc2 expression did not overlap with any of the other markers (Figures 5S2, J-L). With the *Tdc2-GAL4* experiment in the AG, both vGlut-40XMYC (Figures 5S2, M and Q) and 6XV5-vMAT (Figures 5S2, N and R) are present in an overlapping configuration (Figure 5S2, U). The distribution of 5-HT is distinct from any of the other markers (Figure 5S2, V-X). In the AGs the morphology of the SGNs (Figures 5S2, D and H) is distinct from that of the OGNs (Figures 5S2, P and T).

Since OGNs and SGNs are intermingled in tightly fasciculated neuron bundles, we used intersectional labeling to visualize the glutamate transporter vGlut-40XV5 and the monoamine transporter 6XV5-vMAT in each class separately. Together, the results show that both vesicular transporters are expressed in OGNs and SGNs in an overlapping distribution while the OGNs and SGNs remain anatomically distinct throughout the male reproductive tract.

### Expansion microscopy reveals prominent co-occurrence of vGlut and vMAT on ED synaptic vesicles of both OGNs and SGNs

Although there is apparent overlap between vGlut-40XMYC and 6XV5-vMAT in both SGNs and OGNs it is not possible to resolve whether they reside on the same or different synaptic vesicles using standard microscopy methods. This constraint is due to the ~250nm diffraction limit of light at visible wavelengths (ABBE 1873). To overcome this limitation, expansion microscopy was performed on EDs using co-conditional expression of vGlut-40XMYC and 6XV5-vMAT separately in SGNs and OGNs as above. The protocol utilized in these experiments reported 10X expansion in a single round (DAMSTRA et al. 2023) and this agreed well with our empirically determined expansion factor of 10.47X based on the size comparison of nuclei area in muscles of the unexpanded and expanded EDs (Figure 5S5, A and B, respectively). In a representative example of expanded SGNs, 6XV5-vMAT (Figure 5S3, A) and vGlut-40XMYC (Figure 5S3, B) revealed a significantly overlapping distribution (Figure 5S3, C). To quantify the co-occurrence of vGlut-40XMYC and 6XV5-vMAT the BIOP JACop ImageJ plug-in was used to calculate a Mander’s co-efficient, a metric that calculates the fraction of one signal that co-occurs with another. The output of the BIOP JACop plug-in revealed substantial co-occurrence of 6XV5-vMAT and vGlut-40XMYC (Figures 5S3, D-I) including a graphical representation (Figure 5S3, J). For the SGNs, the numerical Mander’s coefficients for 6XV5-vMAT co-occurrence with vGlut-40XMYC were 67.9% and vGlut-40XMYC co-occurrence with 6XV5-vMAT were 57.2% (Figure 5S3, K).

Expanded OGNs yielded similar results. In a representative example, 6XV5-vMAT (Figure 5S4, A) and vGlut-40XMYC (Figure 5S4, B) exhibited substantial overlap (Figure 5S4, C). The output of the BIOP JACop plug-in also revealed majority co-occurrence in image (Figure 5S4, D-I) and graphical form (Figure 5S4, J). For the OGNs, the numerical values of the Mander’s coefficients were 67.4% for 6XV5-vMAT co-occurrence with vGlut-40XMYC and 75.6% for vGlut-40XMYC co-occurrence with 6XV5vMAT (Figure 5S4, K).

The results of the expansion microscopy experiments indicate that in both SGNs and OGNs the majority of ED synaptic vesicles are positive for both vGlut-40XMYC and 6XV5-vMAT and thereby they co-release glutamate and either OA (OGNs) or 5-HT (SGNs). This demonstration of a neurotransmitter co-release mechanism may be a conserved strategy for synchronizing rapid gland and muscle activity during copulation that has general implications across species for how co-transmitter neurons mediate complex motor behaviors.

### OGNs and SGNs express synaptic markers common to other neuromuscular junctions

In addition to providing information on whether a given synapse is primarily excitatory or modulatory, identifying vesicular transporters and synaptic markers allows mapping of active release sites to determine how pre- and post-synaptic specializations align. Such information is essential for (i) elucidating how each neurotransmitter influences muscle contractility and secretory function, and (ii) predicting how synaptic efficacy might change during prolonged copulation or in response to neuromodulatory feedback. To further characterize the synapses of the neurons innervating the male reproductive system immunostaining was performed with vGlut-40XMYC, 6XV5-vMAT, and either of three well-established Drosophila synaptic markers including Synapsin (Syn)-synaptic vesicles, Bruchpilot (Brp)-active zones, and Discs-large (Dlg-Drosophila homolog of mammalian PSD-95)-post-synaptic density. In the SV, strong Syn staining was observed (Figures 6, C and F) that exhibited extensive co-localization with vGlut-40XMYC (Figure 6, H) and 6XV5-vMAT (Figure 6, I). Similar results were obtained in the ED and AGs. Abundant Syn expression was observed (Figure 6, C’ and F’) that exhibited near precise overlap with vGlut-40XMYC (Figure 6, H’) and 6XV5-vMAT (Figure 6H, I’). For the AG, robust Syn expression was observed (Figures 6A, C’’ and F’’) that co-localized strongly with vGlut-40XMYC (Figure 6A, H’’) and 6XV5-vMAT (Figure 6A, I’’). It is notable that a significantly higher density of Syn expression was observed in the SV (Figure 6A, F) compared with the AGs (Figure 6A, F’) and ED (Figure 6A. F’’), presumably indicating greater neurotransmitter output.

The active zone marker Brp exhibited strong expression in the SV (Figure 6S1, C and G) that overlapped almost entirely with 6XV5-vMAT. Brp expression in the ED and AG was noticeably sparser as compared to the SV. The sparse Brp expression in the ED (Figure 6S1, K and O) overlapped with 6XV5-vMAT. Similarly, in the AGs, Brp expression was sparse and overlapped with 6XV5-vMAT (Figure 6S1, S and W).

The post-synaptic density marker Dlg was expressed broadly throughout the male reproductive system (Figure 6S2, C). In the SV, the bulk of Dlg expression is predominantly in the epithelial cells with lower levels of expression in the muscles (Figure 6S2, G and K) and no preferential post-synaptic accumulation (Figures 6S2, H and L). Similar results were observed in the ED and AGs. In the ED at the level of the muscle Dlg expression is diffuse (Figure 6S2, O) and does not accumulate post-synaptically opposite synapses in the muscles (Figure 6S2, P). In the AGs, Dlg expression is also diffuse in the muscles (Figure 6S2, T) and does not accumulate post-synaptically opposite synapses in the muscle (Figure 6C, U). Images of Dlg expression in the epithelial layer of the ED (Figure 6C, Q) and AG (Figure 6C, V) reveal apparent membrane localization in a honeycomb pattern. The lack of post-synaptic accumulation of Dlg opposite the synapses of SGNs and OGNs in the male reproductive system is reminiscent of larval type II OGNs and distinct from larval type I glutamatergic neurons that exhibit prominent post-synaptic concentration of Dlg (PARNAS et al. 2001). It is also notable that there is no vGlut-40XMYC that does not co-localize with 6XV5-vMAT, indicating there are no neurons innervating the SVs, AGs, or ED that uses glutamate as their sole small molecule neurotransmitter. The absence of synapses with post-synaptic Dlg accumulation, a characteristic of type I glutamate-only larval neurons, also supports this assertion. Defining the molecular architecture of these synapses is critical for understanding how they package, release, and receive neurotransmitters to shape the strength and specificity of communication between motor neurons and target organs.

### LDCVs are present in OGNs and SGNs

In addition to synaptic transmission via synaptic vesicles, neurons are also known to communicate using neuropeptides released via Large Dense Core Vesicles (LDCVs). To determine whether neurons innervating the male reproductive system contain LDCVs, immunostaining was performed with the LDCV marker IA2-GFP (YU et al. 2025) in combination with vGlut-40XMYC and 6XV5-vMAT. Abundant IA2-GFP expression was observed throughout the male reproductive system (Figure 6S3, C). In the SVs, IA2-GFP (Figure 6S3, F and L) localizes to the same pre-synaptic regions of the neurons as vGlut-40XMYC (Figures 6S3, D and J) and 6XV5-vMAT (Figures 6S3, E and K). In the highest resolution images, there appears to be some overlap between IA2-GFP and vGlut-40XMYC (Figure 6S3, N) as well as between IA2-GFP and 6XV5-vMAT (Figure 6S3, O), but a substantial fraction of IA2-GFP distributes in close proximity to, but is distinct from, vGlut-40XMYC and 6XV5-vMAT. This distribution pattern is not entirely surprising as it is consistent with the prior observation that vesicular neurotransmitter transporters have been detected on LDCVs (YU et al. 2025).

Similar results were observed in the ED and AGs. In the ED, prominent IA2-GFP expression was evident (Figures 6S4, C and I) that partially co-localized with vGlut-40XMYC (Figures 6S4, E and K) and 6XV5-vMAT (Figures 6S4, F and L). Considerable IA2-GFP expression was also apparent in the AGs (Figures 6S4, O and U) that partially overlapped with vGlut-40XMYC (Figures 6S4, Q and W) and 6XV5-vMAT (Figures 6S4, R and X). This finding of pronounced expression of IA2-GFP throughout the pre-synaptic terminals of neurons innervating the male reproductive system suggest substantial LDCVs are present in both SGNs and OGNs, and that these neurons are thus communicating not just via small molecule neurotransmitters inside synaptic vesicles but also by neuropeptides contained within LDCVs.

### No cholinergic or GABAergic transmission at OGN or SGN synapses

Although to our knowledge there have been no motor neurons reported in Drosophila that use acetylcholine or GABA as a neurotransmitter, to rule out these possibilities for the male reproductive system, Drosophila containing genome-edited 7XMYC-vAChT and 9XV5-vGAT were immunostained for their corresponding epitope tags. Not unexpectedly, no expression of 7XMYC-vAChT was observed in the SVs, ED, or AGs (Figures 6S5, C, G, and K, respectively). Similarly, no expression of 9XV5-vGAT was observed in the SVs, ED, or AGs (Figures 6S5, D, H, and L, respectively), indicating that neither acetylcholine nor GABA are used as neurotransmitters in the OGNs and SGNs of the Drosophila internal male reproductive system.

### GAL4 drivers for OA, 5-HT, and glutamate receptors express in the muscles of male reproductive organs

**Figure 7.**
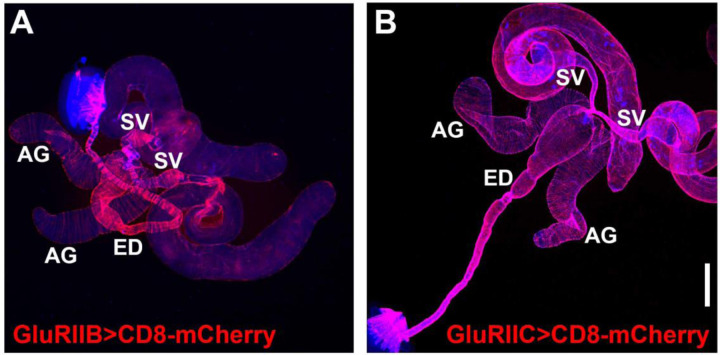
Glutamate receptor GAL4 expression patterns in Drosophila male reproductive system. A) GluRIIB; B) GluRIIC. Scale bar: 200μm.

Given that OGNs and SGNs innervate the male reproductive system, an obvious prediction that follows is that neurotransmitter receptors for glutamate, OA, and 5-HT should be expressed in the muscles of the male reproductive system. As a first step in testing this prediction, GAL4 drivers for each of these neurotransmitters were paired with the *UAS-CD8-mCherry* reporter and assessed for expression. As it is well established that the GluRIIA-E/kainate type glutamate receptors mediate synaptic transmission at Drosophila NMJs (DIANTONIO 2006; HE AND DICKMAN 2025), GAL4 drivers for all five were assessed. GAL4 drivers for GluRIIA, GluRIID, and GluRIIE showed no expression in the male reproductive system, but they also exhibited no expression in other adult or larval muscles where they are known to be expressed. These results suggest that the GAL4 drivers for these three GluRII receptors are simply non-functional and are therefore non-informative. However, GluRIIB-GAL4 and GluRIIC-GAL4 exhibited broad expression in the musculature of the male reproductive system (Figures 7, A and B, respectively). This suggests glutamate is used for signaling throughout the male reproductive system, consistent with the widespread expression of vGlut in pre-synaptic terminals.

OA receptor GAL4 drivers for OAMB, OAα2R, Oct-TyrR, OAβ1R, OAβ2R, and OAβ3R were assessed for expression in the male reproductive system. OAMB-GAL4 exhibited expression in the muscles of the SVs and ED, as well as epithelial cells of the AGs (Figure 7S1, A). OAα2R-GAL4 displayed strong expression in the muscles of the SVs, weak expression in the muscles of the AGs, and no expression in the ED (Figure 7S1, B). Oct-TyrR-GAL4 showed strong expression in non-muscle cells of the distal AG, muscle expression in the AGs, ED, and ejaculatory bulb, expression in the testes, but no expression in the muscles of the SVs (Figure 7S1, C). OAβ2R-GAL4 exhibited strong expression in the epithelial cells of the ED, muscle expression in the SVs, ED, and ejaculatory bulb, expression in the testes, but no muscle expression in the AGs (Figure 7S1, E). OAβ1R-GAL4 and OAβ3R-GAL4 exhibited broad expression in the neurons innervating the male reproductive system (Figures 7S1, D and F, respectively) but no muscle expression, suggesting they are not mediating OA signaling in the muscles. OAβ3R-GAL4 also displayed expression in the testes. These results suggest OA receptors play a prominent role in the functioning of the male reproductive system and differentially mediate OA signaling in specific parts of the male reproductive system based on their restricted, non-uniform expression patterns.

5-HT receptor GAL4 drivers for 5-HT1A, 5-HT2A, 5-HT-1B, 5-HT2B, and 5-HT7 were also assessed for expression in the male reproductive system. 5-HT1A-GAL4, 5-HT1B-GAL4, and 5-HT2B-GAL4 exhibited broad expression in the neurons innervating the male reproductive system, but no muscle expression (Figures 7S2, A, C, and D, respectively), suggesting these receptors are not mediating 5-HT signaling in the muscles. 5-HT2A exhibited expression specifically in the epithelial cells of the SVs (Figure 7S2, B). 5-HT7 showed substantial expression in neurons, but most strikingly, strong expression in the muscles of the distal SVs, including the sphincter region, with a discrete boundary of expression (arrows, Figure 7S2, E).

### GluRIIA is expressed in the SVs and AGs, but not the ED

**Figure 8.**
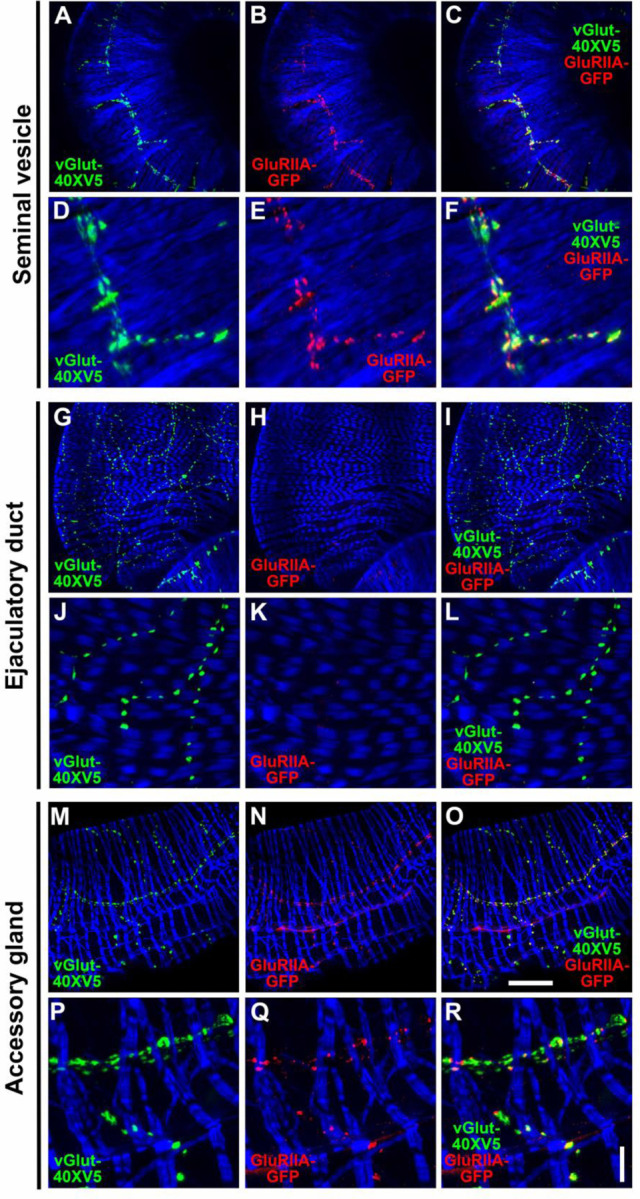
GluRIIA-GFP expression in the SV, ED, and AG of the Drosophila male reproductive system. A-F) SV. A, D) vGlut-40XV5; B,E) GluRIIA-GFP; C, F) vGlut-40XV5, GluRIIA-GFP overlay. G-L) ED. G, J) vGlut-40XV5; H, K) GluRIIA-GFP; I, L) vGlut-40XV5, GluRIIA-GFP overlay. M-R) AG. M, P) vGlut-40XV5; N, Q) GluRIIA-GFP; O, R) vGlut-40XV5, GluRIIA-GFP overlay. Scale bars: O-50μm; R-10μm.

While informative for determining cellular expression, GAL4 driven reporters for genes of interest do not provide critical mechanistic information about the subcellular distribution of the corresponding proteins. To gain further insight into the role of OA, 5-HT, and glutamate receptors in the function of the male reproductive system the expression patterns of several receptors were assessed using epitope-tagging. To determine the subcellular distribution of the ionotropic GluRII/kainate-type glutamate receptors known to mediate fast excitatory glutamatergic transmission at other Drosophila NMJs (DIANTONIO 2006; HE AND DICKMAN 2025), the expression of a previously characterized genome-edited GluRIIA-GFP fly strain in which GFP has been inserted near the carboxy-terminus of GluRIIA at its endogenous genomic location was evaluated in the male reproductive system. In the SV, GluRIIA-GFP (Figure 8, B and E) exhibited punctate expression that paralleled, but was complementary to, that of the glutamatergic synaptic vesicle marker vGlut-40XV5 (Figure 8, A and D). This distribution of GluRIIA-GFP is not unlike its distribution at the NMJ of third instar larva, optimally localized post-synaptically to bind glutamate released from pre-synaptic terminals (BECKERS et al. 2024). Surprisingly, no GluRIIA-GFP was observed in the ED (Figures 8, H and K) despite abundant expression of vGlut-40XV5 (Figures 8, G and J). Expression of GluRIIA-GFP in the AGs (Figures 8, N and Q) was similar to that in the SVs in that it closely paralleled vGlut-40XV5 expression (Figure 8, M and P) but was complementary to it. Finding GluRIIA-GFP expression in the SVs and AGs, but not the ED, and the expression of GluRIIB in all three tissues based on the *GluRIIB-GAL4* expression pattern, suggest the SVs and AGs use a mixture of glutamate receptors containing one or the other of the mutually exclusive GluRIIA or GluRIIB subunits in combination with the common GluRIIC, D, and E subunits, while all glutamate receptors in the ED solely utilize GluRIIB. As GluRII receptors containing GluRIIA generate higher currents than those that contain GluRIIB (DIANTONIO et al. 1999), the lower GluRIIB currents are apparently sufficient for a functional ED.

### OA receptors exhibit distinct expression patterns in the muscles, neurons, and epithelial cells of the male reproductive system

OA receptors are modulatory G-protein coupled receptors (GPCRs) that mediate slower-acting but longer-lasting effects than ionotropic receptors. To visualize expression of the OA receptors OAMB, OAα2R, and OAβ2R, CRISPR/Cas9 genome editing was used to create conditional alleles of each receptor fused at their carboxy termini to a multimerized epitope tag for enhanced detection sensitivity. These genome edits are at the endogenous genomic location of each gene and their expression is thus under the control of their complete endogenous regulatory regions. For visualizing expression in the male reproductive system, germline inversion or excision variants were generated that converted conditional expression to constitutive expression.

OAMB-10XV5 exhibited strong expression in the epithelial cells of the ED and AGs with limited expression in muscles (Figure 8S1, A). Co-immunostaining with the synaptic vesicle marker Synapsin was conducted to visualize pre-synaptic terminals (Figure 8S1, B). In the SVs, higher resolution images revealed low level expression of OAMB-10XV5 in muscles as well as obvious expression in pre-synaptic neurons (Figures 8S1, D and G) that largely overlapped with Syn (Figures 8S1, F and I). OAMB-10XV5 was similarly distributed in the ED with low-level expression in muscle and clearly discernible expression in pre-synaptic neurons (Figure 8S1, J) based on substantial overlap with Syn (Figure 8S1, L).

Strong OAMB-10XV5 expression was also observed in the epithelial cells of the ED in a honeycomb pattern (Figure 8S1, M and P) reminiscent of Dlg and presumably associated with the plasma membrane. OAMB-10XV5 expression in the epithelial cells of the AGs was similar to its expression in the epithelial cells of the ED with prominent signal in a honeycomb pattern (Figure 8S2, A and D). OAMB-10XV5 also exhibited low-level expression in the muscles of the AG and expression in the pre-synaptic neurons (Figure 8S2, G) by virtue of significant overlap with Syn (Figure 8S2, I). Prominent OAMB-10XV5 expression was also observed in the ejaculatory bulb. Low-level expression of OAMB-10XV5 was detected in the muscles of the ejaculatory bulb as was expression in the pre-synaptic neurons (Figure 8S2, J and M) as evidenced by mostly overlapping expression with Syn (Figure 8S2, L and O). In the non-muscular portion of the ejaculatory bulb, OAMB-10XV5 expression was readily discernable in the epithelial cells in a honeycomb pattern (Figure 8S2, J and P).

OAα2R-20XV5 exhibited strong expression in the muscles of both the SVs and more distal regions of the proximal ED near where it connects with the ejaculatory bulb, as well as lower-level expression in the AGs (Figure 8S3, A). Higher resolution images of OAα2R-20XV5 in the SV indicate expression is restricted to the muscle (Figure 8S3, G) as there is no apparent overlap with Syn (Figure 8S3, I). A cross-section of the SV muscle reveals OAα2R-20XV5 is localized to the membrane of the muscle (Figure 8S3, J), as would be expected for a GPCR. In the AGs, OAα2R-20XV5 is expressed in the muscles (Figure 8S3, M and P) as well as in the pre-synaptic neurons as evidenced by co-localization with Syn (Figure 8S3, O and R). OAα2R-20XV5 expression ends abruptly just after the SVs fuse into the sphincter that regulates the movement of sperm into the ED (Figure 8S4, A and D). OAα2R is expressed in the pre-synaptic neurons innervating the sphincter with little to no expression in the sphincter muscles (Figure 8S4, C and F). In the proximal region of the anterior ED prominent OAα2R-20XV5 expression is observed throughout the innervating neurons with little to no OAα2R-20XV5 in the muscles (Figures 8S4, G, I, J, and L). In contrast, in the distal region of the anterior ED, robust expression of OAα2R-20XV5 is detected in the muscles (Figures 8S4, M, P, and S) but not in the pre-synaptic neurons (Figures 8E, O, R, and U). This complementary pattern of OAα2R-20XV5 in neurons but not muscles in the proximal ED and muscles but not neurons in the distal ED suggests these different regions of the ED are exhibiting a differential response to OA. As in the SV, OAα2R-20XV5 localizes to the membrane of the muscles as evident in a cross-sectional image (Figure 8E, S). Interestingly in this region, Syn expression is inside the muscles of the ED (Figure 8E, T), potentially suggesting the muscles are being stimulated from the inside, or alternatively, that the neurons are stimulating the underlying epithelial cells. As pre-synaptic neuronal expression of adrenoreceptors in mammals has been shown to be release-inhibiting (BROWN AND GILLESPIE 1957; STARKE 1972), including in the urinary bladder (SOMOGYI AND DE GROAT 1990) and vas deferens (O'CONNOR et al. 1999; SCHEIBNER et al. 2001), OAα2R expression in the pre-synaptic neurons of the internal male reproductive system may also function via inhibitory auto-reception.

OAβ2R-40XV5 exhibits strong expression in the epithelial cells of the ED, the muscles of the ejaculatory bulb, the muscles of the SVs near the point of fusion, the testes, with lower-level muscle expression in the SVs, ED, and AGs (Figure 8S5, A). Higher resolution images of the SV reveal obvious expression in the muscle (Figure 8S5, D and G) but not in the pre-synaptic neurons as there is no apparent overlap with Syn (Figures 8S5, F and I). A cross-sectional image of the SV reveals OAβ2R-40XV5 is localized to the plasma membrane of muscle cells (Figure 8S5, J). In higher resolution images of the ED, moderate levels of OAβ2R-40XV5 are present in the muscles (Figures 8S5, M and P) but not in the innervating pre-synaptic neurons as there is no obvious overlap with Syn (Figures 8S5, O and R). At the junction where the SVs fuse into a single duct, OAβ2R-40XV5 expression is strong prior to fusion but drops off abruptly at the point of full fusion (arrow, Figure 8S6, A). The strongest levels of OAβ2R-40XV5 expression throughout the entire male reproductive system is in what appears to be the epithelial cells that line the ED (Figure 8S6, A). A cross-section of the ED reveals the distribution of OAβ2R-40XV5 in epithelial cells is exclusively at the apical surface (Figure 8S6, D). Also notable, there is another boundary of OAβ2R-40XV5 expression in the SVs somewhat prior to fusion (solid arrow, Figure 8S6, G). Higher resolution images of OAβ2R-40XV5 expression at the point of SV fusion shows the sharp dip in expression at the point of full fusion (arrow, Figure 8S6, J). A cross-sectional image also reveals the drop in expression and membrane localization of OAβ2R-40XV5 in the muscle (Figure 8S6, M). Similar to the proximal anterior ED, the more distal region of the anterior ED exhibits strong expression in the muscles, and especially the underlying epithelial cells (Figures 8S7, A and D), In the AGs, OAβ2R-40XV5 expression is manifest in the muscles (Figures 8S7, G and J) but does not overlap with the pre-synaptic neurons as evidenced by the lack of overlap with Syn (Figures 8S7, I and L). Prominent OAβ2R-40XV5 expression was also observed in the ejaculatory bulb (Figure 8S7, M and P). As β2 adrenoreceptors relax bladder muscles in mice (WUEST et al. 2009), OAβ2R may relax muscles in the male reproductive system.

Octopamine receptor expression in the internal organs of the male reproductive tract was predicted based on the dense OGN innervation of these tissues. Although OA receptor presence in these muscles aligns with their role in modulating contractile strength and rhythm, the organ-specific differences in receptor subtype abundance could only be established empirically. Epithelial expression implies that octopamine influences non-neuronal functions such as protein secretion or barrier permeability, but the precise cellular responses remain to be defined. Mapping OAMB, OAα2R, and OAβ2R to discrete muscle, neuronal, and epithelial compartments provides mechanistic information about how octopamine fine-tunes contraction, secretion, and presynaptic feedback across the male reproductive tract, offering an evolutionary framework for adrenergic control of fertility and pinpointing cell-specific targets for modulating reproductive output.

### 5-HT7 is widely expressed in the male reproductive system but most prominently in the sphincter region

Since the *5-HT7-GAL4* reporter delineates a sharp posterior boundary in the SV, endogenous 5-HT7 receptor expression was evaluated to pinpoint where 5-HT may directly modulate reproductive tract motility. To assess the expression pattern of the 5-HT7 receptor, a 20XV5 tag was added to the carboxy-terminus by CRISPR/Cas9 genome editing at its endogenous genomic location. 5-HT7–20XV5 localizes to the muscles of the SV just before and after the junction, the epithelial cells of the AGs, and the ejaculatory bulb (Figures 8S8, A). Higher resolution images of the SV reveal low level expression in muscles (Figures 8S8, D and G), as well as expression in the pre-synaptic neurons innervating the male reproductive system as evidenced by co-localization with Syn (Figures 8S8, F and I). In the ED, 5-HT7–20XV5 is expressed in the muscles (Figures 8S8, J and M), but unlike the SV, it is not expressed the pre-synaptic neurons as it does not appear to co-localize with Syn (Figures 8S8, L and O). Expression levels of 5-HT7–20XV5 increase in the SV near the point of fusion and are maximal from just before the junction all the way to the terminus in the ED (Figure 8S9, A). Expression levels of 5-HT7–20XV5 appear to increase at a discrete boundary point on the SVs prior to fusion (arrow, Figure 8S9, D). This boundary of increasing expression at this same region of the SV is highly reminiscent of OAβ2R-40XV5 (Figure 8S6, G). A higher resolution image of the sphincter region of the SV post-fusion reveals high levels of 5-HT7–20XV5 expression (Figure 8S9, G) but not in the innervating pre-synaptic neurons based on the absence of co-localization with Syn (Figure 8S9, I). A cross-section of this same region reveals the 5-HT7–20XV5 protein localizes to muscle membranes (Figure 8S9, J). A high-resolution image of the terminus of the sphincter confirms 5-HT7–20XV5 expression extends all the way to the end (Figure 8S9, M). This observation of 5-HT7–20XV5 expression throughout the fused sphincter region of the SV contrasts with that of OAα2R-20XV5 (Figure 8S4, D) and OAβ2R-40XV5 (Figure 8S6, A), both of which end at the point of full SV fusion and suggests 5-HT plays a more important role than OA in sphincter regulation. 5-HT7–20XV5 expression is also elevated in the muscles at the terminus of the AGs (Figure 8S9, P). In the AGs, 5-HT7–20XV5 is most prominently expressed in the underlying epithelial cells (Figures 8S10, A and D). An AG cross-section reveals 5-HT7–20XV5 localizes exclusively to the apical surface of the epithelial cells (Figure 8S10, G), highly reminiscent of OAβ2R-40XV5 localization to the apical surface of epithelial cells of the ED (Figure 8S6, D). Nearer the surface of the AGs, low-level expression of 5-HT7–20XV5 is detected in the muscle (Figure 8S10, J) and obvious expression is observed in the innervating pre-synaptic neurons based on overlapping expression with Syn (Figure 8S10, L). 5-HT7–20XV5 expression was also observed in the ejaculatory bulb (Figures 8S10, M). Interestingly, none of the OA or serotonin receptors exhibited preferential accumulation in the post-synaptic muscle opposite the pre-synaptic terminals, as observed for GluRIIA. This observation implies volume transmission may play a significant role in modulating muscle activity in the male reproductive system. Neurotransmitter signaling via volume transmission has previously been proposed for the Drosophila female reproductive system based on expression patterns of OA receptors(ROHRBACH et al. 2024a).

### SGNs are essential for male fertility but OGNs are dispensable

Having established that both OGNs and SGNs innervate the male reproductive system an obvious question follows: what are their roles in male fertility? It has previously been reported that OA-deficient flies are male fertile and female sterile (MONASTIRIOTI et al. 1996; COLE et al. 2005). However, in Tβh and Tdc2 mutant flies that fail to synthesize OA, glutamate signaling in the OGNs innervating the male reproductive system is still intact. To determine if simultaneously silencing the OGNs innervating the male reproductive system for both glutamate and OA signaling causes sterility, *Tdc2-GAL4-DBD* was combined with *AbdB-AD* and *UAS-BONT-C* to block all synaptic transmission in these neurons. This intersectional combination results in the silencing of only the Tdc2 neurons in the posterior VNC by virtue of the restricted expression of the AbdB-AD hemidriver to the posterior few segments of the VNC. The result was the same as eliminating OA signaling alone, males were fertile, and females were sterile (Table 1), thus establishing that neither OA nor glutamate signaling is required for fertility in the OGNs innervating the male reproductive system. The observed female sterility validates the silencing function of the UAS-BONT-C transgene.

To assess the requirement of the SGNs innervating the male reproductive system for fertility, flies containing TRH-DBD, AbdB-AD, and UAS-BONT-C were generated. Both males and females of this genotype were sterile (Table 1). The observation that females were also sterile, while no serotonergic innervation of the female reproductive system has been reported, suggests the male sterility is due to 5-HT neurons common to both males and females, and thus the male sterility cannot be unambiguously attributed to the SGNs innervating the male reproductive system.

**Figure 9.**
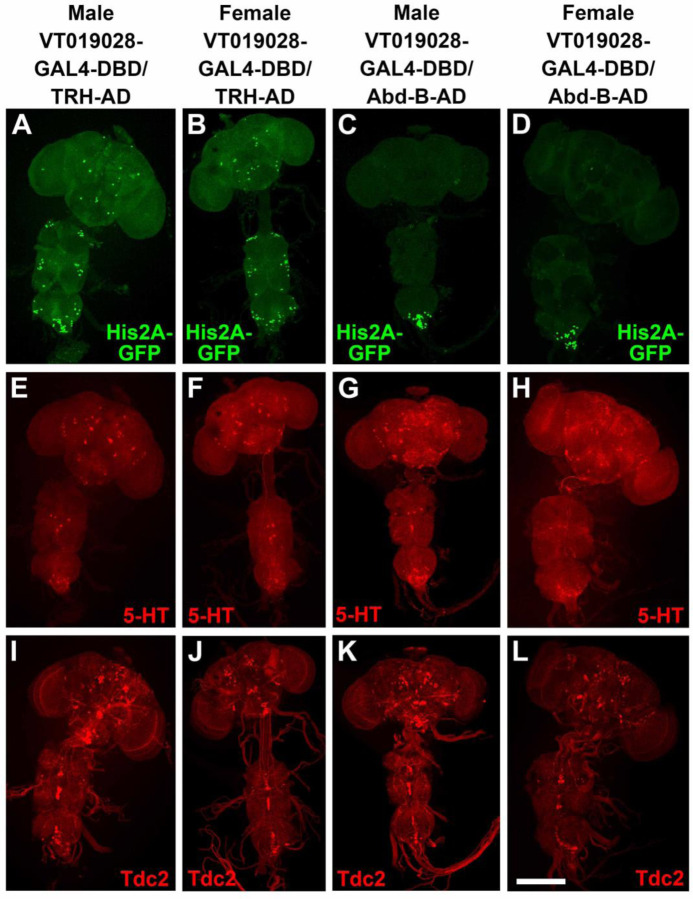
Low resolution images of nuclear expression of *VT019028-GAL4-DBD* in combination with *TRH-AD* and *AbdB-AD* in Drosophila male and female adult nervous system relative to 5-HT and Tdc2. A-D) nuclear expression using HIS2A-GFP marker. A) male *VT019028-GAL4-DBD*/*TRH-AD*; B) female *VT019028-GAL4-DBD*/*TRH-AD*; C) male *VT019028-GAL4-DBD*/*AbdB-AD*; B) female *VT019028-GAL4-DBD*/*AbdB-AD*. E-H) 5-HT expression. E) male *VT019028-GAL4-DBD*/TRH-AD; F) female *VT019028-GAL4-DBD*/*TRH-AD*; G) male *VT019028-GAL4-DBD*/*AbdB-AD*; H) female *VT019028-GAL4-DBD*/*AbdB-AD*. Tdc2 expression. I) male *VT019028-GAL4-DBD*/*TRH-AD*; J) female *VT019028-GAL4-DBD*/*TRH-AD*; K) male *VT019028-GAL4-DBD*/*AbdB-AD*; L) female *VT019028-GAL4-DBD*/*AbdB-AD*. Scale bar: 200μm.

In an attempt to identify a split-GAL4 combination that more specifically targets SGNs innervating the male reproductive system, several dozen hemi-drivers that express in neurons whose cell bodies are located in the most posterior region of the VNC were screened for male sterility in combination with UAS-BONT-C and either *TRH-AD* or *TRH-GAL4-DBD*, as appropriate. One line, *VT019028-GAL4-DBD* was identified that resulted in male sterility and female fertility, suggesting silencing of male-specific sexually dimorphic neurons, and not neurons common to both males and females, were responsible for the male sterility. The *TRH-AD*/*VT019028-GAL4-DBD* driver in combination with a *UAS-His2A-GFP* nuclear reporter results in expression in sparse neurons in the brain and VNC as well as around two dozen neurons at the posterior tip of the VNC (Figure 9, A). Female flies of the same genotype exhibited a similar sparse pattern of expression in the brain and VNC with the exception that there were noticeably fewer His2A-GFP expressing neurons at the tip of the VNC (Figure 9, B). The neurons most likely responsible for male sterility are those unique to males. The *VT019028-GAL4-DBD* driver was also assessed for fertility in combination with *AbdB-AD* and *UAS-BONT-C* with the same result of male sterility and female fertility (Table 1). The expression patterns of *VT019028-GAL4-DBD* with *AbdB-AD* in combination with *UAS-His2A-GFP* reveals restricted expression to the tip of the ventral nerve cord with noticeably more neurons in the male (Figure 9A, C and D). These results with *AbdB-AD* narrow the neurons responsible for the male sterility to the tip of the VNC. The expression patterns of 5-HT and Tdc2 in these immunostains are also shown (Figures 9, E-H, and I-L, respectively).

To determine if any of the *VT019028-GAL4-DBD* neurons intersecting with *TRH-AD* or *AbdB-AD* are serotonergic or octopaminergic, higher resolution images of the tip of the VNC were examined. In the full confocal stack image, Three His2A-GFP neurons are clearly visible that overlap with 5-HT (Figure 9S1, D). However, it has previously been shown that there are both dorsal (~10 neurons) and ventral (~9 neurons) clusters of serotonergic neurons innervating the male reproductive system (BILLETER et al. 2006b) and some neurons may thus be obscured from a dorsal perspective. A partial stack containing the dorsal cluster reveals the same three neurons (Figure 9S1, I). However, a ventral view of these neurons shows a fourth neuron overlapping with 5-HT (Figure 9S1, K). A partial stack of the ventral cluster reveals three His2A-GFP neurons overlapping with 5-HT (Figure 9S1, O). There also appears to be one or two Tdc2 neurons overlapping with His2A-GFP. However, examination of three-dimensional images revealed they are only in close proximity but not overlapping.

Similar results were obtained with *VT019028-GAL4-DBD* neurons intersecting with *AbdB-AD*. Four His2A-GFP neurons overlap with 5-HT in a full confocal stack view (Figure 9S2, D). These four neurons are visible in the dorsal serotonergic cluster (Figure 9S2, I), while three additional His2A-GFP neurons overlapping with 5-HT are apparent in the ventral serotonergic neuron cluster (Figure 9S2, N). One His2A-GFP neuron appears to overlap with Tdc2 (Figure 9S2, E), but this apparent overlap is not reproduced in the dorsal (Figure 9S2, J) or ventral (Figure 9S2. O) partial stacks. The innervation of *VT019028-GAL4-DBD* neurons with *TRH-AD* and *AbdB-AD* visualized with the *UAS-CD8-mCherry* reporter is similarly broad in the SVs, ED, and AGs (Figure 9S3, A and B, respectively). Taken together, these results strongly suggest synaptic transmission in the SGNs included in the *VT019028-GAL4-DBD* intersections with *TRH-AD* and *AbdB-AD* that innervate the male reproductive organs are required for male fertility, although a role for the small number of other interneurons at the tip of the VNC in common between both combinations cannot be excluded.

To rule out the possibility that the male sterility observed in the *VT019028-GAL4-DBD* intersections with *UAS-BONT-C* and either *TRH-AD* and *AbdB-AD* is due to failure to mate, mating assays were performed where single males and single females of the different genotypes were placed together in a well of a 12-well plate and videotaped. The mating success rates of *VT019028-GAL4-DBD*/*TRH-AD* and *VT019028-GAL4-DBD*/*AbdB-AD* were 81.25% (n=16) and 57.9% (n=28), respectively. These results demonstrate the observed male sterility is not due to failure to copulate.

**Figure 10.**
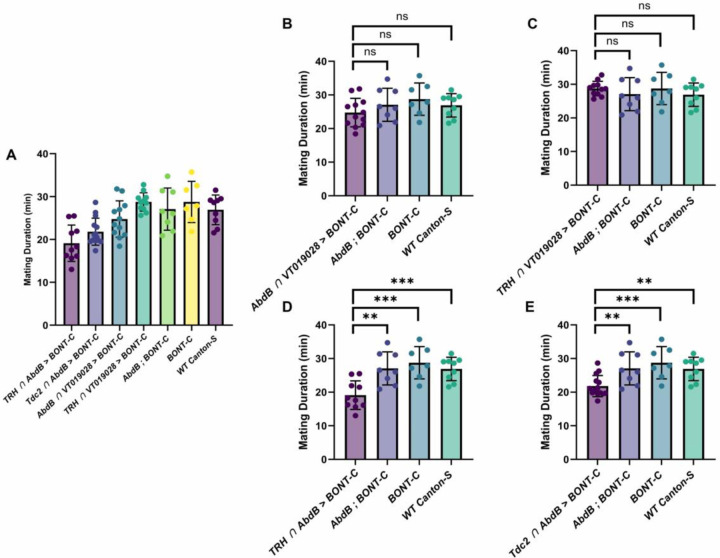
Comparison of mating duration between genotypes. Bar plots show the mean ± SD for each group with individual data points overlaid on each bar. Statistical comparisons were made using unpaired two-tailed t tests where appropriate. Asterisks above indicate significance levels: p < 0.001 (**), p < 0.0001 (***), ns = not significant. (A) Columns from left to right: TRH ∩ AbdB > BONT-C: n= 10, mean= 19.11, SD=4.251. Tdc2 ∩ AbdB > BONT-C: n=14, mean=21.80, SD= 3.155. AbdB ∩ VT019028 > BONT-C: n=12, mean=24.75, SD=4.247. TRH ∩ VT019028 > BONT-C: n=11, mean=28.74, SD=2.155. AbdB ; BONT-C: n=8, mean=27.07, SD=4.907. BONT-C: n=7, mean=28.75, SD=4.809. WT Canton-S: n=9, mean=26.91, SD=3.473.

In these mating assays, mating duration was also assessed along with several control genotypes. Mean mating durations for *VT019028-GAL4-DBD*/*TRH-AD* and *VT019028-GAL4-DBD*/*AbdB-AD* were 28.74 and 24.75 minutes, respectively (Figure 10, A). This was not significantly different from *Canton-S* wildtype controls (26.91), and other controls missing one or more of the components necessary for silencing *AbdB-AD*, *UAS-BONT-C* (27.07) and *UAS-BONT-C* alone (28.75) (Figures 10, B and C). These results are in line with previously reported mating durations for Drosophila (CRICKMORE AND VOSSHALL 2013). Interestingly, silencing all 5-HT neurons (*TRH-GAL4-DBD* ∩ *AbdB-AD*) or all OA neurons (*TRH-GAL4-DBD* ∩ *AbdB-AD*) innervating the male reproductive system resulted in statistically significant reductions in mating duration, 19.11 (Figure 10B, D) and 21.8 (Figure 10B, E), respectively.

To assess the distribution of sperm in the reproductive system during mating by *VT019028-GAL4-DBD*/*TRH-AD* > *UAS-BONT-C* sterile males, a Protamine-GFP reporter that fluorescently labels sperm nuclei was incorporated into the genotype. For this experiment, control and experimental males were placed in petri dishes with virgin females, separated 10 minutes after the initiation of mating, immediately dissected, and the isolated male reproductive systems video recorded using a stereofluorescent microscope. In the control males, ProtB-GFP sperm were readily visible in the ED (Figure 10S1, A, Video 1). In contrast, in the experimental males, no ProtB-GFP sperm were observed (Figure 10S1, B, Video 2). These results demonstrate that the male sterility is due to a failure of the sphincter that regulates the flow of sperm between the SV and ED to open and thus the defect is with the emission phase of ejaculation.

### Glutamate signaling in OGNs and SGNs is dispensable for male fertility

In the *VT019028-GAL4-DBD*/*TRH-AD* > *UAS-BONT-C* sterile males, 5-HT and glutamate signaling are both abolished due to the BONT-C blockade of synaptic transmission. To determine if glutamate signaling alone is required in these neurons, a conditional allele of vGlut was incorporated into this genotype such that specifically glutamate signaling was eliminated while serotonergic signaling remained intact. These males were determined to be fertile. To ascertain whether there were changes in the level of fertility, fertility assays were conducted on 5-day post-eclosion males. No changes in fertility as compared to controls were observed (Figure 10S2, A). Immunostaining the AGs of control and experimental flies confirmed the effectiveness of the conditional vGlut allele by the presence of vGlut in control flies and its absence in experimental flies (Figure 10S3, A and D, respectively). As glutamate has been shown to promote neuron survival as neurons age (SHEN et al. 2018; BUCK et al. 2023), to determine if fertility might be altered in older males, the same 5-day old males used in the previous fertility experiment were held in isolation from females until they were 30 days post-eclosion and the experiment was repeated. While there was not unexpectedly a modest decrease in fertility with age, no differences in fertility were apparent between the control and experimental genotypes (Figure 10S2, B). These results establish that glutamatergic signaling is not required in *VT019028-GAL4-DBD*/*TRH-AD* neurons for fertility. To determine if more extensive elimination of glutamate signaling in the OGNs and SGNs would alter fertility, glutamate was specifically eliminated in both classes of neurons. No reductions in fertility were observed in 5-day post-eclosion males (Figure 10S2, A). These results indicate glutamate is not required in either OGNs or SGNs for fertility.

### The sphincter region of the SV initiates ED peristaltic waves

Neuron-independent peristaltic waves are known to occur spontaneously in the male ED of Drosophila that persist even when all nerves innervating the male reproductive system have been severed (NORVILLE et al. 2010). To gain a better understanding of the muscle activity occurring during these waves in both the ED and throughout the rest of the male reproductive system, a fly strain was generated that expressed the Ca++ sensor GCAMP8m specifically in muscles under control of the myosin heavy chain promoter (*MHC-GCAMP8m*). This fly strain allows visualization of the changes in Ca++ levels that occur in the muscles during these peristaltic waves. As expected, waves of fluorescence are visualizable coincident with peristaltic waves of muscle contractions in the ED (video 3). These peristaltic waves originate not in the ED but in the sphincter region of the SV just prior to and after the point of SV fusion (video 4), although the region of the SV prior to the fusion point that is involved in initiation varies (video 5). Muscle contractions in other regions of the male reproductive system are also apparent. These contractions are weak and seemingly random in the SV (video 6), AG (video 7), and testes (video 8), but occasionally in the AG strong coordinated contractions are observed (video 9). Together, these imaging data reveal that the seminal-vesicle sphincter acts as an autonomous pacemaker, launching neuron-independent peristaltic waves that drive ejaculatory-duct motility.

## Discussion

The male reproductive tract of flies and mammals have obvious morphological differences, yet they share many essential anatomical features and a common organizational logic. During copulation Drosophila males deliver seminal fluid and ~4000–6000 sperm to females during a tightly choreographed ~20-minute window (KAUFMAN 1942; GILBERT 1981). This involves the emission of sperm and seminal fluid from their sites of origin into the ED where they mix and are subsequently expelled into the female reproductive tract. Despite the critical importance of ejaculation, its neuronal mechanisms have remained poorly defined, hindering broader insights into the neural logic of male fertility. Here we close this gap by demonstrating that two distinct yet intermingled classes of amine/glutamate neurons, OGNs and SGNs, coordinate the ejaculatory sequence that ensures successful reproduction. Their transmitter pairs confer the capacity to co-release both fast excitatory and slower acting aminergic modulatory neurotransmitters. Differential expression of ionotropic glutamate receptors and aminergic metabotropic neurotransmitter receptors combine in post-synaptic muscle across the male reproductive system to confer an additional level of complexity to coordinately regulate fluid secretion, organ contractility, and directional sperm movement, to the end of optimizing male fertility.

This system can serve as a genetically tractable model to gain general insights into how multi-transmitter neurons coordinate and modulate interrelated action sequences critical for complex organ function. Lessons learned from this system may have implications for understanding how males of Drosophila and other species adapt their reproductive strategies to a dynamic environment. Additionally, understanding the mechanisms by which this system operates has the potential to identify neuromodulation strategies for male infertility and prostate cancer as multi-transmitter sympathetic and parasympathetic neurons innervating human male reproductive organs have been implicated in these conditions (BURNSTOCK 2009; MAGNON et al. 2013; BURNSTOCK 2014; DROBNIS AND NANGIA 2017; DWIVEDI et al. 2021).

### Mechanisms of multi-transmitter neuron-mediated transfer of sperm and seminal fluid

Movement of sperm and seminal fluid through the male reproductive tract requires an alternating series of coordinated muscle contractions and relaxations. Based on the observed co-packaging of vMAT and vGlut in the majority of synaptic vesicles in the ED of both the OGNs and SGNs, combined with the observed non-uniform distribution of neurotransmitter receptors for OA, 5-HT, and glutamate in neurons and muscles, potential pre- and post-synaptic mechanisms for regulating the activity of the muscles of the internal male reproductive system are possible.

Pre-synaptic expression of the α-type OA receptors OAMB and OAα2R may facilitate alternating bursts of action potential-dependent neurotransmitter release by diminishing pre-synaptic neurotransmitter release via an auto-receptive mechanism. As α-type adrenergic and noradrenergic receptors have been consistently shown to inhibit neurotransmitter release in multiple rodent species in both the peripheral and central nervous system including the vas deferens, heart, and brain when expressed in pre-synaptic neurons (STARKE 1972; TRENDELENBURG et al. 1994; MIYAZAKI et al. 1998; SCHEIBNER et al. 2001; TRENDELENBURG et al. 2003), the presence of OAMB and OAα2R in the pre-synaptic terminals of neurons innervating the internal male reproductive organs may have the effect of suppressing neurotransmitter output from pre-synaptic terminals and shortening the duration of muscle contractions. Thus, pre-synaptic expression of OAMB and OAα2R may have the effect of increasing the cycling frequency of muscle contractions and relaxations.

As glutamate is acting via post-synaptic GluRII-type receptors, its effect will almost certainly be excitatory with the result of eliciting fast muscle contractions. In contrast, the functional consequences of OA and 5-HT are not so clear cut. Either of these amines could enhance muscle contractions or facilitate muscle relaxation, depending on the neurotransmitter receptor(s) receiving the signal. In muscles of the mammalian prostate and lower urinary tract, α-type adrenergic and noradrenergic receptors have been demonstrated to augment muscle contractions while β-type receptors have been found to facilitate muscle relaxation (HONDA et al. 1985; MICHEL AND VRYDAG 2006; WUEST et al. 2009). In contrast, OAMB and OAβ2R have been shown to enhance muscle relaxation and contraction, respectively in the Drosophila female reproductive system (DESHPANDE et al. 2022).

5-HT7 receptors are known to promote smooth muscle relaxation in the urinary tract, gut, oviduct, and blood vessels of mammals (CARTER et al. 1995; LEUNG et al. 1996; PRINS et al. 1999; INOUE et al. 2003; RECIO et al. 2009; CHANG CHIEN et al. 2015; MATSUMOTO-MIYAI et al. 2015). Although muscle contraction receives preferential focus, muscle relaxation is also critically important for the execution of motor programs (GOULDING et al. 2014; WONG AND LANGE 2014; GOWDA et al. 2018; HIRAMOTO et al. 2021) and 5-HT7 most likely plays a role in mediating muscle relaxation in the internal male reproductive system. An additional confounding factor is that OAα2R has been shown to respond not just to OA but also to 5-HT (QI et al. 2017). The observations of the presence of both muscle contraction enhancing, and muscle relaxation promoting, OA and 5-HT receptors in the same muscles of the internal male reproductive organs indicates the mechanisms of neuronal regulation of their activity are complex and may even involve synergistic effects that can only be established empirically.

### A potential timing mechanism mediated by multi-transmitter neurons

Pre-synaptic release of glutamate acting on post-synaptic ionotropic GluRII-type glutamate receptors will cause near immediate, short-term, contraction of muscles. At least in the ED, our results show that the majority of synaptic vesicles co-package either OA or 5-HT along with glutamate, and thus simultaneous co-release is being used as a mechanism of neurotransmitter signaling. The amines OA or 5-HT acting on post-synaptic metabotropic OA and 5-HT receptors will have the effect of either prolonging the length of, or augmenting the relaxation of, muscles, dependent on the specific aminergic neurotransmitter receptors involved. These latter effects will be delayed relative to the glutamate-induced fast muscle contractions due to the discrepancy in the time course of action between ionotropic and metabotropic receptors. Thus, co-releasing a fast-acting excitatory neurotransmitter acting through an ionotropic receptor simultaneous with a modulatory aminergic neurotransmitter acting through slower acting GPCRs in the same multi-transmitter neuron may create a sequential timing mechanism whereby fast glutamate-mediated muscle contractions always precede either amine-mediated prolonging of muscle contraction or facilitation of muscle relaxation. A single neuron co-releasing one fast and one slow neurotransmitter may thus have the advantage over two distinct neurons separately releasing these transmitters in that the former can more effectively synchronize, and at the same time, stagger events that require precise timing.

### Potential for adaptation to environment

The complexity of neuronal control of male reproductive function revealed in this investigation exposes a variety of possibilities for adaptive regulation to dynamic environmental conditions. Polygynous Drosophila males are well-established to alter the quantity and quality of their ejaculate based on environmental circumstances, especially the level of competition with other males (BRETMAN et al. 2009; MOATT et al. 2014), but also due to nutrient availability (MACARTNEY et al. 2021) and the presence of pathogens (LIAO et al. 2024). The male reproductive system may adapt to environmental conditions by tuning octopamine and serotonin signaling, adjusting the levels of their biosynthetic enzymes, the vesicular monoamine transporter vMAT, or relevant receptors to alter the amount of sperm and seminal fluid released during copulation. A potential mechanism for altering OA and/or 5-HT levels is extra-synaptic volume transmission whereby the level of OA or 5-HT secreted into the hemolymph in locations anatomically distant from the male reproductive system. Thus, global levels of OA and 5-HT in the hemolymph could be adjusted based on environmental conditions and this signal could then impact the male reproductive system to optimize ejaculate quality and quantity. Finally, the presence of OAMB and 5-HT7 on epithelial cells suggest the potential for the regulation of the quality of seminal fluid as the expression of these receptors implies, they could differentially alter gene expression in the epithelial cells in response to changing levels of OA and 5-HT. The results of this study create a foundation for mechanistic investigations into the molecular basis by which male reproductive resources are adaptively modulated.

### Potential for signaling via neuropeptides

The broad abundant expression of the LDCV reporter IA2 in the neurons innervating the internal organs of the male reproductive system suggests a significant role for neuropeptide signaling since neuropeptides are known to be packaged into LDCVs (PELLETIER AND LECLERC 1979; KREINER et al. 1986). Bolstering the prospect of this possibility, numerous neuropeptides across evolutionary distant insect species have been shown to alter the frequency and/or amplitude of the spontaneous peristaltic waves in the ED of male insect reproductive systems (MARCINIAK et al. 2017; CIZMAR et al. 2019; LANGE et al. 2023). Multiple neuropeptides have also been implicated in the function of the internal male reproductive organs of mammals, including humans, (SEGAWA et al. 1978; ALLEN et al. 1982; TAINIO 1995). Given these considerations, it thus seems likely that neuropeptides outside the scope of this investigation also play a role in regulating Drosophila male reproduction.

### The SV/ED junction is a control center

Immediately proximal to the ED, SVs fuse into a single duct that forms the sphincter regulating sperm flow from the SV to the ED during mating. The differential expression patterns of OAα2R, OAβ2R, and 5-HT7 in this region of the SV is intriguing. The extent of OAα2R and OAβ2R expression ends very shortly after the merge, at precisely the point where full SV fusion occurs and there is no longer a partition separating the ducts (BAIRATI 1968). Only 5-HT7 expression extends into the fully fused SV that constitutes the sphincter separating the SV from the ED. This expression pattern implies a prominent role for 5-HT7 in sphincter regulation. Previous reports of 5-HT7 mediating relaxation of smooth muscle in the mammalian gut (CARTER et al. 1995; PRINS et al. 1999), urinary bladder neck (RECIO et al. 2009), cardiovascular system (LEUNG et al. 1996; CHANG CHIEN et al. 2015), lymphatic system (CHAN AND VON DER WEID 2003), and oviduct (INOUE et al. 2003) raises the possibility it may function to relax the sphincter muscles during copulation to permit sperm flow. Additionally, expression of OAβ2R and the likely relaxation-promoting 5-HT7 receptors increases sharply in the SV just prior to the point of fusion. Perhaps not coincidentally, this limit of expression corresponds with the distal-most extent of sperm localization in the SV where relaxation of the SV muscles must also occur for sperm to move along the duct to the ED. It is also notable, as revealed in the Ca++ imaging experiments, that the sphincter region is the site of initiation of the peristaltic waves of muscle contractions that are promulgated through the ED. The SV/ED junction is thus a critical control center for both regulation of sperm flow into the ED during mating and initiating neuron-independent peristaltic waves in the ED.

### Molecular continuity of neurotransmitter signaling between males and females?

The expression of 5-HT7 and OAβ2R on the apical surface of the epithelial cells lining the AG and ED, respectively, is surprising because it implies that levels of 5-HT and OA are fluctuating in the lumen of these organs. The origin of these neurotransmitters is not obvious but if OA and 5-HT are present in the lumen of the AG and ED, another intriguing possibility is that these neurotransmitters are transferred to the female at mating for signaling in order to modulate female behavior and/or reproductive activity. A recent study detailed the molecular continuity of proteins in the seminal fluid of males functionally interacting with proteins in the female reproductive tract (MCCULLOUGH et al. 2022). The potential presence of OA and 5-HT in the lumen of the AG and ED raises the possibility that this molecular continuity could extend to neurotransmitters in male seminal fluid signaling to neurotransmitter receptors in the female reproductive tract to convey information such as health, internal state, or environmental conditions to regulate female reproductive output. A detailed examination of the expression patterns of OA receptors in the female reproductive tract indicates this is indeed a possibility, at least for OA signaling (ROHRBACH et al. 2024b).

## Materials and methods

### Fly strains

Stocks from the Bloomington Drosophila Stock Center (NIH P40OD018537) were used in this study. *Tdc2-GAL4* (COLE et al. 2005) RRID:BDSC_9313, *GluRIIA-GAL4*

RRID:BDSC_84637, *GluRIID-GAL4* RRID:BDSC_84638, and *TRH-GAL4*

RRID:BDSC_84694 (DENG et al. 2019)

*vGlut-GAL4* RRID:BDSC_60312, *vGlut-GAL4-DBD* (DIAO et al. 2015)

*Tdc2-AD* (DIONNE et al. 2018) RRID:BDSC_601902

*TRH-GAL4-DBD* RRID:BDSC_70371

*TRH-AD* RRID:BDSC_70975

*GluRIIB-GAL4* RRID:BDSC_60333 and *GluRIIE-GAL4* RRID:BDSC_60332 (DIAO et al. 2015)

*Oct-TyrR-GAL4* RRID:BDSC_77735 and *GluRIIC-GAL4* RRID_83268 (LEE et al. 2018)

*GluRIIA-GFP* RRID:BDSC_99517 (BECKERS et al. 2024)

*5-HT1A-GAL4* RRID:BDSC_84588, *5-HT1B-GAL4* RRID:BDSC_84589 , *5-HT2A-GAL4* RRID:BDSC_84590, *5-HT2B-GAL4* RRID:BDSC_84591, and *5-HT7-GAL4* RRID:BDSC_84592 (DENG et al. 2019)

*T*β*H-GFP* RRID:BDSC_80113 (LI-KROEGER et al. 2018)

*fru-GAL4* RRID:BDSC_66696

*UAS-CD8-mCherry* RRID:BDSC_27392

*ProtB-GFP* (MANIER et al. 2010) RRID:BDSC_58406

*AbdB-AD* (DIAO et al. 2024)

*vGlut-AD* (GAO et al. 2008)

*IA2-GFP* (YU et al. 2025)

*UAS-BONT-C* (HAN et al. 2022)

*dsx-GAL4-DBD* (SHIRANGI et al. 2016)

*vGlut-40XV5* and *vGlut-40XMYC* (STOWERS 2025)

*B2RT-7XMYC-vAChT* (TISON et al. 2020)

*RSRT-9XV5-vGAT* (CERTEL et al. 2022)

*OAMB-GAL4*, *OAα2R-GAL4*, *OAβ1R-GAL4*, *OAβ2R-GAL4*, *OAβ3R-GAL4* (MCKINNEY et al. 2020)

*FRT-F3 OAMB-10XV5* (BAKSHINSKA et al. 2025)

*B3RT-Tdc2-LexA*, *B3RT-vGlut-LexA*, *RSRT-STOP-RSRT-6XV5-vMAT*, *UAS-B3* and *20XUAS-R* (SHERER et al. 2020)

*20XUAS-B2* (WILLIAMS et al. 2019)

*13LexAop-6XmCherry* (SHEARIN et al. 2014)

### Stocks original to this report

*Tdc2-GAL4-DBD* (*JK65C*)

*B3RT-Tdc2-LexA* germline excision

*B3RT-vGlut-LexA* germline excision

*RSRT-6XV5-vMAT* germline excision

FRT-F3-OAα2R-20XV5

*FRT-F3-OAα2R-20XV5* germline inversion

B2RT-STOP-B2RT-OAβ2R-40XV5

*B2RT-OAβ2R-40XV5* germline excision

5-HT7–20XV5

*MHC-GCAMP8m* (*VK27*)

### Entry clones

The *R4-GAL4-DBD-R3* entry clone contains a 640bp insert encoding the GAL4 DNA binding domain. The *L1-MHC-5’Reg-R5* entry clone contains 5254bp of upstream regulatory sequence from the Drosophila Myosin Heavy Chain (MHC) gene. The *L5-GCAMP8m-L2* entry clone contains a 1276bp insert encoding GCAMP8m (ZHANG et al. 2023). Entry clones were generated as previously described (PETERSEN AND STOWERS 2011).

### Expression clones

*Tdc2-GAL4-DBD* was constructed by combining entry clones *L1-Tdc2–5’Reg-L4* and *L3-Tdc2–3’Reg-L2* (SHEARIN et al. 2013) with the *R4-GAL4-DBD-R3* entry clone and the destination vector *pDESTP10* (SHEARIN et al. 2013). *MHC-GCAMP8m* was assembled by combining entry clones *L1-MHC-5’Reg-R5* and *L5-GCAMP8m-L2* with *pDESTP10-SPEC*. *pDESTP10-Spec* is a derivative of *pDESTP10* in which AmpR was replaced with SpecR.

### Donor plasmid assembly

Donor plasmids were assembled starting with gene synthesis of the homology arms (Synbio). The gene synthesis also included recombinase target sites and mutations in the PAM sites associated with the guide RNAs. Homology arms typically extended ~750bp beyond the cut sites of the guide RNAs. STOP cassettes were added via Gibson cloning. Multimerized epitope tags (STOWERS 2025) were added via *Asc I*/*Not I* restriction enzyme cloning. The *OAα2R-FRT-F3–20XV5* donor plasmid contained a pair of *FRT-F3* sites appropriately arranged for conditional expression of OAα2R-20XV5 after inversion/excision by FLP recombinase (FISHER et al. 2017) with the 20XV5 fused to the carboxy-terminus. The *OAβ2R-B2RT-STOP-B2RT-40XV5* donor plasmid contained B2 recombinase target sites flanking a STOP cassette such that OAβ2R-40XV5 was expressed after excision of the STOP cassette with 40XV5 fused to the carboxy-terminus. The 5-HT7–20XV5 donor plasmid contained a 20XV5 multimerized epitope tag fused to the carboxy-terminus but was not designed for conditional expression.

### Guide RNA plasmid assembly

Guide RNAs were assembled in the *pCFD4* double-guide RNA plasmid as previously described (PORT et al. 2014). Two double guide RNA plasmids were used with each donor construct for genome editing. The guide RNA plasmids and the guide RNAs they contain are as follows: *pCFD4-OAα2RGd12*-TCTAAATCAATATCAGTG and AACATAAACATCCTAAAA; *pCFD4-OAα2R-Gd34*-ACCTGTAACAGCACACAT and GGCATGTATCAGCACTAC; *pCFD4-OAβ2R-Gd56*-ATAAATGAGATTATACTC and CATGCATCTGGAATTTAT; *pCFD4-OAβ2R-Gd78*-CGGCGGGCGGATCCCGCC and AGGATGGCAGGGAGTCGA; *pCFD4-5-HT7-Gd56*-TGGGCGATGAGAGGCACG and TGGGCGATGAGAGGCACG; *pCFD4-5-HT7-Gd78*-CCACGAGGACCTCCATTC and GGGTCTTCCCTAGGTTTC.

### Genome editing

For each genome edit, the donor plasmid was injected together with both *pCFD4* guide RNA plasmids into the *nos-Cas9 attP40* fly strain by Rainbow Transgenic Flies. Adult flies that were injected as embryos were crossed to 3^rd^ chromosome balancer stocks. Candidate progeny males from this first cross containing the *TM6b* balancer chromosome were crossed to yw; n-syb-GAL4, UAS-FLP/TM6c (*OAα2R-FRT-F3–20XV5*, *yw*; *20XUAS-B2*; *n-syb-GAL4* (*OAβ2R-B2RT-STOP-B2RT-40XV5*) or *yw*; *Ly*/*TM6c* (*5-HT7–20XV5*) fly strain and larval progeny were screened by immunostaining. Males whose progeny exhibited positive immunostaining were subsequently crossed to a third chromosome balancer stock to establish stable lines.

### Germline excision/inversion

Germline inversion of *OAα2R-FRT-F3–20XV5* was accomplished by crossing to *yw*; *nos-GAL4*; *UAS-FLP*. Males containing all three genetic components were crossed to a third chromosome balancer stock. Individual progeny males from this cross were crossed a second time to the third chromosome balancer stock and non-Tb larva were screened by anti-V5 immunostaining to identify germline excisions. A similar strategy was used with *OAβ2R-B2RT-STOP-B2RT-40XV5* for a germline excision except the initial cross was to *yw*; *nos-GAL4*; *UAS-B2*. *vGlut-LexA* was generated by crossing *B3RT-vGlut-LexA* to *yw*: *nos-GAL4*; *UAS-B3*. Progeny from this cross containing all three genetic components were crossed to a second chromosome balancer stock. Single progeny males were then crossed to a *13XLexAop-6XmCherry* reporter and larva were screened for expression in the ventral nerve cord to identify germline excision positives. A similar strategy was used to create *B3RT-Tdc2-LexA* starting with *B3RT-Tdc2-LexA* (MCKINNEY et al. 2020). *RSRT-6XV5-vMAT* was generated by crossing *RSRT-STOP-RSRT-6XV5-vMAT* with *yw*; *nos-GAL4*; *UAS-R*. Progeny from this cross containing all three genetic components were crossed to a second chromosome balancer stock. Single progeny males were crossed to the second balancer a second time and larval progeny were subjected to anti-V5 immunostaining to identify germline excisions.

### Immunostaining

Male reproductive systems were dissected in PBS and fixed in 4% paraformaldehyde for 30 minutes with rotation. After 2X brief washes with PBS, they were incubated in PBS containing 2% Triton-X 100 and 2% BSA (PBSTB) for at least two hours with rotation. Subsequently, they were incubated in primary antibodies PBSTB for one hour with rotation, followed by overnight incubation at 4°C without rotation to prevent tissue damage. After 3X 5-minute washes in PBSTB, male reproductive systems were incubated for 2–3 hours in PBSTB with secondary antibodies with rotation. After three 5-minute washes in PBS, they were incubated in PBS containing Phalloidin 405 for one hour. After two 5-minute washes in PBSTB and one 5-minute wash in PBS, male reproductive systems were mounted on glass slides in Vectashield Plus (Vector Laboratories, Cat. #H-1900) using a coverslip that was glued on the edges with clear nail polish.

Primary antibodies and dilution factors: The SYN (3C11) mAb 1:20 (KLAGGES et al. 1996). Brp nc82 mAb 1:40 (deposited at the DSHB by Eric Buchner), Dlg 4F3 mAb 1:40 (PARNAS et al. 2001), were obtained from the Developmental Studies Hybridoma Bank, created by the NICHD of the NIH and maintained at The University of Iowa, Department of Biology, Iowa City, IA 52242. Mouse anti-V5 SV5-P-K (Novus NBP2–52703) 1:200; rat anti-V5 SV5-P-K (Novus NBP2–81037) 1:200, rabbit anti-V5 SV5-P-K (Novus NBP2–52653) 1:200; Human anti-V5 (Novus NBP2–81035) 1:200; rat anti-MYC 9E10 (Novus NBP2–81020) 1:200, rabbit anti-MYC 9E10 (Novus NBP2–52636), mouse anti-MYC 4A6; (Millipore Sigma) 1:200, mouse anti-vGlut 1:10 (BANERJEE et al. 2021), mouse anti-GFP 3E6 (Thermo-Fisher A11120) 1:200; Rabbit anti-GFP (Thermo-Fisher G10362); rat anti-mCherry 16D7 (ThermoFisher-M11217) 1:200, rabbit anti-Tdc2 pab0822-P, Covalab) 1:500; rat anti-5-HT YC5 (Abcam ab6336) 1:300.

Secondary antibodies and dilution factors: Goat anti-Mouse Alexa 405 (Thermo-Fisher A31553) 1:400; Goat anti-Mouse Alexa 488 (Jackson Immunoresearch 115-545-166) 1:400; Donkey anti-Rat Alexa 488 (Jackson Immunoresearch 712-546-153) 1:400, Goat anti-Rabbit Alexa 488 (Thermo-Fisher A32731) 1:400, Goat anti-Mouse Alexa 568 (Thermo Fisher A11031) 1:400, Goat anti-Rabbit Alexa 568 (Thermo Fisher A11036)1:400, Donkey anti-Rat Alexa 568 (Thermo-Fisher A78946) 1:400; Goat anti-Mouse Alexa 647 (Thermo-Fisher A32728) 1:400; Goat anti-Rabbit Alexa 647 (Thermo-Fisher A32733) 1:400; Goat anti-Rat Alexa 647 (Thermo-Fisher A48265 1:400); Donkey anti-Human Alexa 790 (Jackson Immunoresearch 709-655-149) 1:400. Muscles were stained with Phalloidin iFluor405 (Abcam AB176752) 1:800.

Confocal microscopy was performed using a Leica Stellaris DMI8 inverted confocal scanning light microscope in the microscopy facility at the Montana State University Center for Biofilm Engineering.

### Expansion microscopy

The Ten-fold Robust Expansion Microscopy (TREx) protocol was used to expand the male ED (ED) (DAMSTRA et al. 2023). Briefly, Drosophila male reproductive systems were dissected and fixed for at least thirty minutes in 4% paraformaldehyde. The ED’s were then dissected out and immunostained as described above. Tissues were then incubated in Acryloyl-X solution, 1:100 dilution (Invitrogen, catalog #2604348), overnight, at room temperature and protected from light. The next day, the ED’s were rinsed in PBS and incubated in TRex solution at 4 C, for 45–60 minutes. 0.15% ammonium persulfate and 0.15% TEMED was added and the TREx mixture was quickly transferred to chamber slides. TREx mixture with ED’s were placed in wells (1 ED per well), coverslipped and allowed to polymerize at 37 C for at least 45 minutes. After gel polymerization, each gel-ED was trimmed to eliminate excess gel. Gel-ED’s were incubated in proteinase K (Biolabs, catalog #P81075, 800U/ml) 1:100 solution, overnight at room temperature. Expansion of gel-ED’s was done the following day. Three washes in water were performed, at least 20 minutes per wash. DAPI (Thermofisher Scientific, catalog# D1306), 2ug/ml, was added in the first wash to aid in tissue visualization. After the third wash, hydrogel-ED’s were trimmed again and placed in chamber slides. 1 hydrogel-ED per well and coverslipped. Imaged expanded ED’s in upright confocal (Leica Stellaris 8 DIVE) equipped with a ×40 NA = 0.8 water objective. z Stack- images were deconvoluted using the Lightning confocal function and 3D images were generated for each image. ED’s typically expanded approximately tenfold.

### Colocalization Analysis

Digitally deconvolved- 3D images of expanded EDs were analyzed using ImageJ software (SCHINDELIN et al. 2012). To exclude background in an unbiased manner, we thresholded all images by using the Otsu threshold and calculated Manders’ coefficients by using the BIOP JACop.plugin. A scatter plot of green and red pixel intensities (associated with each of the two markers, Vmat and Vglut) was generated by using the colocalization function. Manders' coefficient ranges from 0 to 1, indicating no or complete colocalization. Prior to doing the colocalization analysis of each image, a region of interest (ROI) was traced using the stain from the CD8 marker. This ROI corresponded to an axon terminal.

### Mating assays

Mating assays involved placing single 3-7-day old virgin males with single 3-7-day old virgin females in the same well of a 12 well tissue culture plate without anesthesia. The wells of the 12 well plates were half-filled with agarose. Flies were videotaped for three hours. Mating assays were conducted at 25C and 50% humidity in a room dedicated to behavioral assays. Mating was scored by viewing the videos and scorers were blind to the genotypes.

### Fertility assays

The fertility assays involved placing single 5 day old or 30-day old virgin males with four 3-7-day old virgin females in fresh vials without anesthesia for six hours. After six hours the males were removed and the four females returned to the vials for 72hrs at which point the females were removed. Six days after removal of the females the pupae on the walls of the vials were counted. The counting was done blind to the genotypes. Fly culturing for all stages of the fertility assays were conducted at 25C and 50% in an incubator.

### Animal use

All experiments involving *Drosophila melanogaster* were conducted in accordance with approved protocols and standard laboratory practices. *Drosophila melanogaster* is not considered a vertebrate animal and accordingly does not require Montana State University Institutional Animal Care and Use Committee (IACUC) approval.

## Supplementary Material

Supplement 1

## Figures and Tables

**Figure 3.** Expression of *TRH-GAL4* and *Tdc2-LexA* in the Drosophila male reproductive system. A, D, G, J, M, P) *TRH-GAL4*, *UAS-6XGFP*; B, E, H, K, N, Q) *Tdc2-LexA*, *LexAop-6XmCherry*. C, F, I, L, O, R) overlay. Scale bars: O-50μm; R-10μm.

**Table 1.** Male and female fertility phenotypes.

Genotype	Male Fertile	Female Fertile
*yw*; *TRH-GAL4DBD*/*AbdB-AD*, *UAS-BONT-C*	No	No
*yw*; *Tdc2-GAL4DBD*/*AbdB-AD*, *UAS-BONT-C*	Yes	No
*yw*; *VT019028-GAL4-DBD*/*AbdB-AD*, *UAS-BONT-C*	No	Yes
*yw*; *TRH-AD*; *UAS-BONT-C*/*VT019028-GAL4-DBD*	No	Yes
*yw*; *AbdB-AD*/*UAS-BONT-C*	Yes	Yes
*yw*; *UAS-BONT-C*	Yes	Yes
*Canton-S* wildtype	Yes	Yes

## Data Availability

All plasmids and their complete sequences are available upon request. Fly strains original to this publication will be deposited at the Bloomington Drosophila Stock Center or made available upon request.

## References

[R1] Abbe E., 1873 Beitrage zur Theorie des microskops und der mikroskopischen Wahrnehmung. Anatomie IX: 413–468.

[R2] Acebes A., Grosjean Y., Everaerts C. and Ferveur J. F., 2004 Cholinergic control of synchronized seminal emissions in Drosophila. Curr Biol 14: 704–710. 15084286 10.1016/j.cub.2004.04.003

[R3] Allen J. M., Adrian T. E., Tatemoto K., Polak J. M., Hughes J. 1982 Two novel related peptides, neuropeptide Y (NPY) and peptide YY (PYY) inhibit the contraction of the electrically stimulated mouse vas deferens. Neuropeptides 3: 71–77. 6186938 10.1016/0143-4179(82)90001-4

[R4] Alwaal A., Breyer B. N. and Lue T. F., 2015 Normal male sexual function: emphasis on orgasm and ejaculation. Fertil Steril 104: 1051–1060. 26385403 10.1016/j.fertnstert.2015.08.033 PMC4896089

[R5] Bairati A., 1968 [The structure and ultrastructure of the male genital apparatus of the Drosophila melanogaster Meig. 2. The genital duct and accessory glands]. Italian Journal of Zoology 2: 105–182.

[R6] Baker C. A., Guan X. J., Choi M. and Murthy M., 2024 The role of fruitless in specifying courtship behaviors across divergent Drosophila species. Sci Adv 10: eadk1273. 38478605 10.1126/sciadv.adk1273 PMC10936877

[R7] Bakshinska D., Liu W. Y., Schultz R., Stowers R. S., Hoagland A. 2025 Synapse-specific catecholaminergic modulation of neuronal glutamate release. Proc Natl Acad Sci U S A 122: e2420496121. 39793084 10.1073/pnas.2420496121 PMC11725921

[R8] Banerjee S., Vernon S., Jiao W., Choi B. J., Ruchti E. 2021 Miniature neurotransmission is required to maintain Drosophila synaptic structures during ageing. Nat Commun 12: 4399. 34285221 10.1038/s41467-021-24490-1 PMC8292383

[R9] Beckers C. J., Mrestani A., Komma F. and Dannhauser S., 2024 Versatile Endogenous Editing of GluRIIA in Drosophila melanogaster. Cells 13. 10.3390/cells13040323 PMC10887371 38391936

[R10] Beguelini M. R., Puga C. C. I., Martins F. F., Betoli A. H. S., Taboga S. R. 2013 Morphological Variation of Primary Reproductive Structures in Males of Five Families of Neotropical Bats. Anatomical Record-Advances in Integrative Anatomy and Evolutionary Biology 296: 156–167. 10.1002/ar.22613 23117997

[R11] Billeter J. C., and Goodwin S. F., 2004 Characterization of Drosophila fruitless-gal4 transgenes reveals expression in male-specific fruitless neurons and innervation of male reproductive structures. J Comp Neurol 475: 270–287. 15211467 10.1002/cne.20177

[R12] Billeter J. C., Rideout E. J., Dornan A. J. and Goodwin S. F., 2006a Control of male sexual behavior in Drosophila by the sex determination pathway. Curr Biol 16: R766–776. 16950103 10.1016/j.cub.2006.08.025

[R13] Billeter J. C., Villella A., Allendorfer J. B., Dornan A. J., Richardson M. 2006b Isoform-specific control of male neuronal differentiation and behavior in Drosophila by the fruitless gene. Curr Biol 16: 1063–1076. 16753560 10.1016/j.cub.2006.04.039

[R14] Bretman A., Fricke C. and Chapman T., 2009 Plastic responses of male Drosophila melanogaster to the level of sperm competition increase male reproductive fitness. Proc Biol Sci 276: 1705–1711. 19324834 10.1098/rspb.2008.1878 PMC2660996

[R15] Brown G. L., and Gillespie J. S., 1957 The output of sympathetic transmitter from the spleen of the cat. J Physiol 138: 81–102. 13463800 10.1113/jphysiol.1957.sp005839 PMC1363031

[R16] Buck S. A., Rubin S. A., Kunkhyen T., Treiber C. D., Xue X. 2023 Sexually dimorphic mechanisms of VGLUT-mediated protection from dopaminergic neurodegeneration. bioRxiv.

[R17] Burnstock G., 2009 Purinergic cotransmission. Exp Physiol 94: 20–24. 18723580 10.1113/expphysiol.2008.043620

[R18] Burnstock G., 2014 Purinergic signalling in the reproductive system in health and disease. Purinergic Signal 10: 157–187. 24271059 10.1007/s11302-013-9399-7 PMC3944041

[R19] Carro-Juarez M., Cruz S. L. and Rodriguez-Manzo G., 2003 Evidence for the involvement of a spinal pattern generator in the control of the genital motor pattern of ejaculation. Brain Res 975: 222–228. 12763611 10.1016/s0006-8993(03)02686-6

[R20] Carter D., Champney M., Hwang B. and Eglen R. M., 1995 Characterization of a postjunctional 5-HT receptor mediating relaxation of guinea-pig isolated ileum. Eur J Pharmacol 280: 243–250. 8566092 10.1016/0014-2999(95)00195-q

[R21] Castellanos M. C., Tang J. C. and Allan D. W., 2013 Female-biased dimorphism underlies a female-specific role for post-embryonic Ilp7 neurons in Drosophila fertility. Development 140: 3915–3926. 23981656 10.1242/dev.094714 PMC3915572

[R22] Certel S. J., McCabe B. D. and Stowers R. S., 2022 A conditional GABAergic synaptic vesicle marker for Drosophila. J Neurosci Methods 372: 109540. 35219770 10.1016/j.jneumeth.2022.109540 PMC8940707

[R23] Chan A. K., and von der Weid P. Y., 2003 5-HT decreases contractile and electrical activities in lymphatic vessels of the guinea-pig mesentery: role of 5-HT 7-receptors. Br J Pharmacol 139: 243–254. 12770929 10.1038/sj.bjp.0705264 PMC1573860

[R24] Chang Chien C. C., Hsin L. W. and Su M. J., 2015 Activation of serotonin 5-HT(7) receptor induces coronary flow increase in isolated rat heart. Eur J Pharmacol 748: 68–75. 25196212 10.1016/j.ejphar.2014.08.027

[R25] Chehensse C., Facchinetti P., Bahrami S., Andrey P., Soler J. M. 2017 Human spinal ejaculation generator. Ann Neurol 81: 35–45. 27917533 10.1002/ana.24819

[R26] Cizmar D., Roller L., Pillerova M., Slama K. and Zitnan D., 2019 Multiple neuropeptides produced by sex-specific neurons control activity of the male accessory glands and gonoducts in the silkworm Bombyx mori. Sci Rep 9: 2253. 30783175 10.1038/s41598-019-38761-x PMC6381147

[R27] Cole S. H., Carney G. E., McClung C. A., Willard S. S., Taylor B. J. 2005 Two functional but noncomplementing Drosophila tyrosine decarboxylase genes: distinct roles for neural tyramine and octopamine in female fertility. J Biol Chem 280: 14948–14955. 15691831 10.1074/jbc.M414197200

[R28] Crickmore M. A., and Vosshall L. B., 2013 Opposing dopaminergic and GABAergic neurons control the duration and persistence of copulation in Drosophila. Cell 155: 881–893. 24209625 10.1016/j.cell.2013.09.055 PMC4048588

[R29] Damstra H. G. J., Mohar B., Eddison M., Akhmanova A., Kapitein L. C. 2023 Ten-fold Robust Expansion Microscopy. Bio Protoc 13: e4698. 10.21769/BioProtoc.4698 PMC10308184 37397797

[R30] DeLecce T., Vance G. S., Zeigler-Hill V., Welling L. L. M. and Shackelford T. K., 2025 Ejaculate Adjustment in Response to Sperm Competition Risk in Humans. Arch Sex Behav 54: 277–287. 39500803 10.1007/s10508-024-03030-0

[R31] Demerec M., 1950 Biology of Drosophila. Wiley, New York,.

[R32] Deng B., Li Q., Liu X., Cao Y., Li B. 2019 Chemoconnectomics: Mapping Chemical Transmission in Drosophila. Neuron 101: 876–893 e874. 30799021 10.1016/j.neuron.2019.01.045

[R33] Deshpande S. A., Freyberg Z., Lawal H. O. and Krantz D. E., 2020 Vesicular neurotransmitter transporters in Drosophila melanogaster. Biochim Biophys Acta Biomembr 1862: 183308. 32305263 10.1016/j.bbamem.2020.183308 PMC7508792

[R34] Deshpande S. A., Rohrbach E. W., Asuncion J. D., Harrigan J., Eamani A. 2022 Regulation of Drosophila oviduct muscle contractility by octopamine. iScience 25: 104697. 35880044 10.1016/j.isci.2022.104697 PMC9307614

[R35] DiAntonio A., 2006 Glutamate receptors at the Drosophila neuromuscular junction. Int Rev Neurobiol 75: 165–179. 17137928 10.1016/S0074-7742(06)75008-5

[R36] DiAntonio A., Petersen S. A., Heckmann M. and Goodman C. S., 1999 Glutamate receptor expression regulates quantal size and quantal content at the Drosophila neuromuscular junction. J Neurosci 19: 3023–3032. 10191319 10.1523/JNEUROSCI.19-08-03023.1999 PMC6782296

[R37] Diao F., Ironfield H., Luan H., Diao F., Shropshire W. C. 2015 Plug-and-play genetic access to drosophila cell types using exchangeable exon cassettes. Cell Rep 10: 1410–1421. 25732830 10.1016/j.celrep.2015.01.059 PMC4373654

[R38] Diao F., Vasudevan D., Heckscher E. S. and White B. H., 2024 Hox gene-specific cellular targeting using split intein Trojan exons. Proc Natl Acad Sci U S A 121: e2317083121. 38602904 10.1073/pnas.2317083121 PMC11047080

[R39] Dionne H., Hibbard K. L., Cavallaro A., Kao J. C. and Rubin G. M., 2018 Genetic Reagents for Making Split-GAL4 Lines in Drosophila. Genetics 209: 31–35. 29535151 10.1534/genetics.118.300682 PMC5937193

[R40] Drobnis E. Z., and Nangia A. K., 2017 Cardiovascular/Pulmonary Medications and Male Reproduction. Adv Exp Med Biol 1034: 103–130. 29256129 10.1007/978-3-319-69535-8_9

[R41] Dwivedi S., Bautista M., Shrestha S., Elhasasna H., Chaphekar T. 2021 Sympathetic signaling facilitates progression of neuroendocrine prostate cancer. Cell Death Discov 7: 364. 34811362 10.1038/s41420-021-00752-1 PMC8608828

[R42] Eberhard W. G., 1985 Sexual selection and animal genitalia. Harvard University Press, Cambridge, Mass.

[R43] Fisher Y. E., Yang H. H., Isaacman-Beck J., Xie M., Gohl D. M. 2017 FlpStop, a tool for conditional gene control in Drosophila. Elife 6. 10.7554/eLife.22279 PMC5342825 28211790

[R44] Fromme L., Yogui D. R., Alves M. H., Desbiez A. L. J., Langeheine M. 2021 Morphology of the genital organs of male and female giant anteaters (). Peerj 9. 10.7717/peerj.11945 PMC8364315 34447632

[R45] Gage M. J. G., and Baker R. R., 1991 Ejaculate Size Varies with Sociosexual Situation in an Insect. Ecological Entomology 16: 331–337.

[R46] Gao S., Takemura S. Y., Ting C. Y., Huang S., Lu Z. 2008 The neural substrate of spectral preference in Drosophila. Neuron 60: 328–342. 18957224 10.1016/j.neuron.2008.08.010 PMC2665173

[R47] Garbaczewska M., Billeter J. C. and Levine J. D., 2013 Drosophila melanogaster males increase the number of sperm in their ejaculate when perceiving rival males. J Insect Physiol 59: 306–310. 23178803 10.1016/j.jinsphys.2012.08.016

[R48] Gilbert D. G., 1981 Ejaculate Esterast 6 and Initial Sperm Use by Female Drosophila melanogaster. Journal of Insect Physiology 27: 641–650.

[R49] Gomes L. F., Badke J. P., Zama U., Dolder H. and Lino-Neto J., 2012 Morphology of the male reproductive system and spermatozoa in Centris Fabricius, 1804 (Hymenoptera: Apidae, Centridini). Micron 43: 695–704. 22377697 10.1016/j.micron.2012.01.013

[R50] Goulding M., Bourane S., Garcia-Campmany L., Dalet A. and Koch S., 2014 Inhibition downunder: an update from the spinal cord. Curr Opin Neurobiol 26: 161–166. 24743058 10.1016/j.conb.2014.03.006 PMC4059017

[R51] Gowda S. B. M., Paranjpe P. D., Reddy O. V., Thiagarajan D., Palliyil S. 2018 GABAergic inhibition of leg motoneurons is required for normal walking behavior in freely moving Drosophila. Proc Natl Acad Sci U S A 115: E2115–E2124. 29440493 10.1073/pnas.1713869115 PMC5834679

[R52] Guerrero S. M., Calderón M. L., de Pérez G. R. and Pinilla M. P. R., 2004 Morphology of the male reproductive duct system of (Crocodylia, Alligatoridae). Annals of Anatomy-Anatomischer Anzeiger 186: 235–245. 10.1016/s0940-9602(04)80009-8 15255300

[R53] Han Y., Chien C., Goel P., He K., Pinales C. 2022 Botulinum neurotoxin accurately separates tonic vs. phasic transmission and reveals heterosynaptic plasticity rules in Drosophila. Elife 11. 10.7554/eLife.77924 PMC9439677 35993544

[R54] He K., and Dickman D., 2025 Building and modifying diverse synaptic properties: Insights from Drosophila. Curr Opin Neurobiol 92: 102995. 40064134 10.1016/j.conb.2025.102995 PMC12162220

[R55] Hiramoto A., Jonaitis J., Niki S., Kohsaka H., Fetter R. D. 2021 Regulation of coordinated muscular relaxation in Drosophila larvae by a pattern-regulating intersegmental circuit. Nat Commun 12: 2943. 34011945 10.1038/s41467-021-23273-y PMC8134441

[R56] Honda K., Miyata-Osawa A. and Takenaka T., 1985 alpha 1-Adrenoceptor subtype mediating contraction of the smooth muscle in the lower urinary tract and prostate of rabbits. Naunyn Schmiedebergs Arch Pharmacol 330: 16–21. 2864637 10.1007/BF00586704

[R57] Hopkins B. R., Sepil I., Thezenas M. L., Craig J. F., Miller T. 2019 Divergent allocation of sperm and the seminal proteome along a competition gradient in Drosophila melanogaster. Proc Natl Acad Sci U S A 116: 17925–17933. 31431535 10.1073/pnas.1906149116 PMC6731677

[R58] Inoue M., Kitazawa T., Cao J. and Taneike T., 2003 5-HT7 receptor-mediated relaxation of the oviduct in nonpregnant proestrus pigs. Eur J Pharmacol 461: 207–218. 12586216 10.1016/s0014-2999(03)01312-8

[R59] Jois S., Chan Y. B., Fernandez M. P. and Leung A. K., 2018 Characterization of the Sexually Dimorphic fruitless Neurons That Regulate Copulation Duration. Front Physiol 9: 780. 29988589 10.3389/fphys.2018.00780 PMC6026680

[R60] Kaufman B. P. a. D., M., 1942 Utilization of sperm by Female Drosophila Melanogaster. The American Naturalist 76: 445–469.

[R61] Klagges B. R., Heimbeck G., Godenschwege T. A., Hofbauer A., Pflugfelder G. O. 1996 Invertebrate synapsins: a single gene codes for several isoforms in Drosophila. J Neurosci 16: 3154–3165. 8627354 10.1523/JNEUROSCI.16-10-03154.1996 PMC6579133

[R62] Kreiner T., Sossin W. and Scheller R. H., 1986 Localization of Aplysia neurosecretory peptides to multiple populations of dense core vesicles. J Cell Biol 102: 769–782. 3949877 10.1083/jcb.102.3.769 PMC2114117

[R63] Lange A. B., Kisana A., Leyria J. and Orchard I., 2023 The Male Reproductive System of the Kissing Bug, Rhodnius prolixus Stal, 1859 (Hemiptera: Reduviidae: Triatominae): Arrangements of the Muscles and the Myoactivity of the Selected Neuropeptides. Insects 14. 10.3390/insects14040324 PMC10146185 37103139

[R64] Lee G., and Hall J. C., 2001 Abnormalities of male-specific FRU protein and serotonin expression in the CNS of fruitless mutants in Drosophila. J Neurosci 21: 513–526. 11160431 10.1523/JNEUROSCI.21-02-00513.2001 PMC6763814

[R65] Lee P. T., Zirin J., Kanca O., Lin W. W., Schulze K. L. 2018 A gene-specific T2A-GAL4 library for Drosophila. Elife 7. 10.7554/eLife.35574 PMC5898912 29565247

[R66] Leung E., Walsh L. K., Pulido-Rios M. T. and Eglen R. M., 1996 Characterization of putative 5-ht7 receptors mediating direct relaxation in Cynomolgus monkey isolated jugular vein. Br J Pharmacol 117: 926–930. 8851512 10.1111/j.1476-5381.1996.tb15282.x PMC1909429

[R67] Li-Kroeger D., Kanca O., Lee P. T., Cowan S., Lee M. T. 2018 An expanded toolkit for gene tagging based on MiMIC and scarless CRISPR tagging in Drosophila. Elife 7. 10.7554/eLife.38709 PMC6095692 30091705

[R68] Liao A., Cavigliasso F., Savary L. and Kawecki T. J., 2024 Effects of an entomopathogenic fungus on the reproductive potential of Drosophila males. Ecol Evol 14: e11242. 38590549 10.1002/ece3.11242 PMC10999951

[R69] Macartney E. L., Zeender V., Meena A., De Nardo A. N., Bonduriansky R. 2021 Sperm depletion in relation to developmental nutrition and genotype in Drosophila melanogaster. Evolution 75: 2830–2841. 34617270 10.1111/evo.14373 PMC9297908

[R70] Maciel E. D. S., Zieri R. and de Almeida-Santos S. M., 2024 Male genital system of Ameiva ameiva (Squamata: Teiidae). Anat Rec (Hoboken) 307: 3596–3605. 38665006 10.1002/ar.25463

[R71] Magnon C., Hall S. J., Lin J., Xue X., Gerber L. 2013 Autonomic nerve development contributes to prostate cancer progression. Science 341: 1236361. 23846904 10.1126/science.1236361

[R72] Majane A. C., Cridland J. M. and Begun D. J., 2022 Single-nucleus transcriptomes reveal evolutionary and functional properties of cell types in the Drosophila accessory gland. Genetics 220. 10.1093/genetics/iyab213 PMC9097260 34849871

[R73] Manier M. K., Belote J. M., Berben K. S., Novikov D., Stuart W. T. 2010 Resolving mechanisms of competitive fertilization success in Drosophila melanogaster. Science 328: 354–357. 20299550 10.1126/science.1187096

[R74] Marciniak P., Urbanski A., Kudlewska M., Szymczak M. and Rosinski G., 2017 Peptide hormones regulate the physiological functions of reproductive organs in Tenebrio molitor males. Peptides 98: 35–42. 27353004 10.1016/j.peptides.2016.06.006

[R75] Matsumoto-Miyai K., Yoshizumi M. and Kawatani M., 2015 Regulatory Effects of 5-Hydroxytryptamine Receptors on Voiding Function. Adv Ther 32 Suppl 1: 3–15. 26391372 10.1007/s12325-015-0240-2

[R76] McCullough E. L., Whittington E., Singh A., Pitnick S., Wolfner M. F. 2022 The life history of Drosophila sperm involves molecular continuity between male and female reproductive tracts. Proc Natl Acad Sci U S A 119: e2119899119. 35254899 10.1073/pnas.2119899119 PMC8931355

[R77] McKinney H. M., Sherer L. M., Williams J. L., Certel S. J. and Stowers R. S., 2020 Characterization of Drosophila octopamine receptor neuronal expression using MiMIC-converted Gal4 lines. J Comp Neurol 528: 2174–2194. 32060912 10.1002/cne.24883 PMC7998515

[R78] Michel M. C., and Vrydag W., 2006 Alpha1-, alpha2- and beta-adrenoceptors in the urinary bladder, urethra and prostate. Br J Pharmacol 147 Suppl 2: S88–119. 16465187 10.1038/sj.bjp.0706619 PMC1751487

[R79] Miyazaki T., Kobayashi H. and Tosaka T., 1998 Presynaptic inhibition by noradrenaline of the EPSC evoked in neonatal rat sympathetic preganglionic neurons. Brain Res 790: 170–177. 9593880 10.1016/s0006-8993(97)01549-7

[R80] Moatt J. P., Dytham C. and Thom M. D., 2014 Sperm production responds to perceived sperm competition risk in male Drosophila melanogaster. Physiol Behav 131: 111–114. 24769021 10.1016/j.physbeh.2014.04.027

[R81] Monastirioti M., Linn C. E. Jr. and White K., 1996 Characterization of Drosophila tyramine beta-hydroxylase gene and isolation of mutant flies lacking octopamine. J Neurosci 16: 3900–3911. 8656284 10.1523/JNEUROSCI.16-12-03900.1996 PMC6578608

[R82] Norville K., Sweeney S. T. and Elliott C. J., 2010 Postmating change in physiology of male Drosophila mediated by serotonin (5-HT). J Neurogenet 24: 27–32. 20067436 10.3109/01677060903477601

[R83] O'Connor S. C., Brain K. L. and Bennett M. R., 1999 Individual sympathetic varicosities possess different sensitivities to alpha 2 and P2 receptor agonists and antagonists in mouse vas deferens. Br J Pharmacol 128: 1739–1753. 10588930 10.1038/sj.bjp.0702984 PMC1571817

[R84] Oliveira M. S., Serrao J. E., Dias G., Martins L. C. B. and Araújo V. A., 2021 Anatomy and histology of the male reproductive tractof Machtima crucigera (Fabricius, 1775) (Heteroptera: Coreidae). Zoologischer Anzeiger 293: 156–162.

[R85] Parnas D., Haghighi A. P., Fetter R. D., Kim S. W. and Goodman C. S., 2001 Regulation of postsynaptic structure and protein localization by the Rho-type guanine nucleotide exchange factor dPix. Neuron 32: 415–424. 11709153 10.1016/s0896-6273(01)00485-8

[R86] Pauls D., Blechschmidt C., Frantzmann F., El Jundi B. and Selcho M., 2018 A comprehensive anatomical map of the peripheral octopaminergic/tyraminergic system of Drosophila melanogaster. Sci Rep 8: 15314. 30333565 10.1038/s41598-018-33686-3 PMC6192984

[R87] Pavlou H. J., Lin A. C., Neville M. C., Nojima T., Diao F. 2016 Neural circuitry coordinating male copulation. Elife 5. 10.7554/eLife.20713 PMC5114013 27855059

[R88] Pelletier G., and Leclerc R., 1979 Localization of Leu-enkephalin in dense core vesicles of axon terminals. Neurosci Lett 12: 159–163. 460711 10.1016/0304-3940(79)96055-5

[R89] Petersen L. K., and Stowers R. S., 2011 A Gateway MultiSite recombination cloning toolkit. PLoS One 6: e24531. 21931740 10.1371/journal.pone.0024531 PMC3170369

[R90] Pizzari T., Cornwallis C. K., Lovlie H., Jakobsson S. and Birkhead T. R., 2003 Sophisticated sperm allocation in male fowl. Nature 426: 70–74. 14603319 10.1038/nature02004

[R91] Port F., Chen H. M., Lee T. and Bullock S. L., 2014 Optimized CRISPR/Cas tools for efficient germline and somatic genome engineering in Drosophila. Proc Natl Acad Sci U S A 111: E2967–2976. 25002478 10.1073/pnas.1405500111 PMC4115528

[R92] Pound N., and Gage M. J. G., 2004 Prudent sperm allocation in Norway rats, :: a mammalian model of adaptive ejaculate adjustment. Animal Behaviour 68: 819–823.

[R93] Prins N. H., Briejer M. R., Van Bergen P. J., Akkermans L. M. and Schuurkes J. A., 1999 Evidence for 5-HT7 receptors mediating relaxation of human colonic circular smooth muscle. Br J Pharmacol 128: 849–852. 10556917 10.1038/sj.bjp.0702762 PMC1571702

[R94] Qi Y. X., Xu G., Gu G. X., Mao F., Ye G. Y. 2017 A new Drosophila octopamine receptor responds to serotonin. Insect Biochem Mol Biol 90: 61–70. 28942992 10.1016/j.ibmb.2017.09.010

[R95] Recio P., Barahona M. V., Orensanz L. M., Bustamante S., Martinez A. C. 2009 5-hydroxytryptamine induced relaxation in the pig urinary bladder neck. Br J Pharmacol 157: 271–280. 19309355 10.1111/j.1476-5381.2009.00144.x PMC2697801

[R96] Rohrbach E. W., Asuncion J. D., Meera P., Kralovec M., Deshpande S. A. 2024a Heterogeneity in the projections and excitability of tyraminergic/octopaminergic neurons that innervate the Drosophila reproductive tract. Front Mol Neurosci 17: 1374896. 39156129 10.3389/fnmol.2024.1374896 PMC11327148

[R97] Rohrbach E. W., Knapp E. M., Deshpande S. A. and Krantz D. E., 2024b Expression and potential regulatory functions of Drosophila octopamine receptors in the female reproductive tract. G3 (Bethesda) 14. 10.1093/g3journal/jkae012 PMC10917510 38244217

[R98] Scheibner J., Trendelenburg A. U., Hein L. and Starke K., 2001 Stimulation frequency-noradrenaline release relationships examined in alpha2A-, alpha2B- and alpha2C-adrenoceptor-deficient mice. Naunyn Schmiedebergs Arch Pharmacol 364: 321–328. 11683519 10.1007/s002100100432

[R99] Schindelin J., Arganda-Carreras I., Frise E., Kaynig V., Longair M. 2012 Fiji: an open-source platform for biological-image analysis. Nat Methods 9: 676–682. 22743772 10.1038/nmeth.2019 PMC3855844

[R100] Segawa T., Murakami H., Ogawa H. and Yajima H., 1978 Effect of enkephalin and substance P on sympathetic nerve transmission in mouse vas deferens. Jpn J Pharmacol 28: 13–19. 206750 10.1254/jjp.28.13

[R101] Sepil I., Hopkins B. R., Dean R., Thezenas M. L., Charles P. D. 2019 Quantitative Proteomics Identification of Seminal Fluid Proteins in Male Drosophila melanogaster. Mol Cell Proteomics 18: S46–S58. 30287546 10.1074/mcp.RA118.000831 PMC6427238

[R102] Shearin H. K., Dvarishkis A. R., Kozeluh C. D. and Stowers R. S., 2013 Expansion of the gateway multisite recombination cloning toolkit. PLoS One 8: e77724. 24204935 10.1371/journal.pone.0077724 PMC3799639

[R103] Shearin H. K., Macdonald I. S., Spector L. P. and Stowers R. S., 2014 Hexameric GFP and mCherry reporters for the Drosophila GAL4, Q, and LexA transcription systems. Genetics 196: 951–960. 24451596 10.1534/genetics.113.161141 PMC3982691

[R104] Shen H., Marino R. A. M., McDevitt R. A., Bi G. H., Chen K. 2018 Genetic deletion of vesicular glutamate transporter in dopamine neurons increases vulnerability to MPTP-induced neurotoxicity in mice. Proc Natl Acad Sci U S A 115: E11532–E11541. 30442663 10.1073/pnas.1800886115 PMC6298109

[R105] Sherer L. M., Catudio Garrett E., Morgan H. R., Brewer E. D., Sirrs L. A. 2020 Octopamine neuron dependent aggression requires dVGLUT from dual-transmitting neurons. PLoS Genet 16: e1008609. 32097408 10.1371/journal.pgen.1008609 PMC7059954

[R106] Shirangi T. R., Wong A. M., Truman J. W. and Stern D. L., 2016 Doublesex Regulates the Connectivity of a Neural Circuit Controlling Drosophila Male Courtship Song. Dev Cell 37: 533–544. 27326931 10.1016/j.devcel.2016.05.012

[R107] Siwicki K. K., and Kravitz E. A., 2009 Fruitless, doublesex and the genetics of social behavior in Drosophila melanogaster. Curr Opin Neurobiol 19: 200–206. 19541474 10.1016/j.conb.2009.04.001 PMC2716404

[R108] Somogyi G. T., and de Groat W. C., 1990 Modulation of the release of [3H]norepinephrine from the base and body of the rat urinary bladder by endogenous adrenergic and cholinergic mechanisms. J Pharmacol Exp Ther 255: 204–210. 2170623

[R109] Soni K. K., Jeong H. S. and Jang S., 2022 Neurons for Ejaculation and Factors Affecting Ejaculation. Biology (Basel) 11. 10.3390/biology11050686 PMC9138817 35625414

[R110] Starke K., 1972 Alpha sympathomimetic inhibition of adrenergic and cholinergic transmission in the rabbit heart. Naunyn Schmiedebergs Arch Pharmacol 274: 18–45. 4403611 10.1007/BF00501004

[R111] Stowers R. S., 2025 Multimerized Epitope Tags for High Sensitivity Protein Detection. G3 (Bethesda). 10.1093/g3journal/jkaf070 PMC12134993 40191928

[R112] Susic-Jung L., Hornbruch-Freitag C., Kuckwa J., Rexer K. H., Lammel U. 2012 Multinucleated smooth muscles and mononucleated as well as multinucleated striated muscles develop during establishment of the male reproductive organs of Drosophila melanogaster. Dev Biol 370: 86–97. 22841645 10.1016/j.ydbio.2012.07.022

[R113] Tainio H., 1995 Peptidergic innervation of the human prostate, seminal vesicle and vas deferens. Acta Histochem 97: 113–119. 7771181 10.1016/S0065-1281(11)80212-6

[R114] Tayler T. D., Pacheco D. A., Hergarden A. C., Murthy M. and Anderson D. J., 2012 A neuropeptide circuit that coordinates sperm transfer and copulation duration in Drosophila. Proc Natl Acad Sci U S A 109: 20697–20702. 23197833 10.1073/pnas.1218246109 PMC3528491

[R115] Tison K. V., McKinney H. M. and Stowers R. S., 2020 Demonstration of a Simple Epitope Tag Multimerization Strategy for Enhancing the Sensitivity of Protein Detection Using Drosophila vAChT. G3 (Bethesda) 10: 495–504. 31767639 10.1534/g3.119.400750 PMC7003071

[R116] Tomas A. L., Bento M. A. G., Mutti L. D., Zara F. J. and Greco L. S. L., 2019 New insights in the male anatomy, spermatophore formation, and sperm structure in Atyidae: The red cherry shrimp. Invertebrate Biology 138: 17–28.

[R117] Trendelenburg A. U., Meyer A., Klebroff W., Guimaraes S. and Starke K., 2003 Crosstalk between presynaptic angiotensin receptors, bradykinin receptors and alpha 2-autoreceptors in sympathetic neurons: a study in alpha 2-adrenoceptor-deficient mice. Br J Pharmacol 138: 1389–1402. 12721093 10.1038/sj.bjp.0705223 PMC1573813

[R118] Trendelenburg A. U., Starke K. and Limberger N., 1994 Presynaptic alpha 2A-adrenoceptors inhibit the release of endogenous dopamine in rabbit caudate nucleus slices. Naunyn Schmiedebergs Arch Pharmacol 350: 473–481. 7870186 10.1007/BF00173016

[R119] Truitt W. A., and Coolen L. M., 2002 Identification of a potential ejaculation generator in the spinal cord. Science 297: 1566–1569. 12202834 10.1126/science.1073885

[R120] Verhulst E. C., and van de Zande L., 2015 Double nexus--Doublesex is the connecting element in sex determination. Brief Funct Genomics 14: 396–406. 25797692 10.1093/bfgp/elv005 PMC4652034

[R121] Villella A., and Hall J. C., 2008 Neurogenetics of courtship and mating in Drosophila. Adv Genet 62: 67–184. 19010254 10.1016/S0065-2660(08)00603-2

[R122] Wedell N., and Cook P. A., 1999 Butterflies tailor their ejaculate in response to sperm competition risk and intensity. Proceedings of the Royal Society B-Biological Sciences 266: 1033–1039.

[R123] Wigby S., Brown N. C., Allen S. E., Misra S., Sitnik J. L. 2020 The Drosophila seminal proteome and its role in postcopulatory sexual selection. Philos Trans R Soc Lond B Biol Sci 375: 20200072. 33070726 10.1098/rstb.2020.0072 PMC7661438

[R124] Williams J. L., Shearin H. K. and Stowers R. S., 2019 Conditional Synaptic Vesicle Markers for Drosophila. G3 (Bethesda) 9: 737–748. 30635441 10.1534/g3.118.200975 PMC6404611

[R125] Wolfner M. F., 1997 Tokens of love: functions and regulation of Drosophila male accessory gland products. Insect Biochem Mol Biol 27: 179–192. 9090115 10.1016/s0965-1748(96)00084-7

[R126] Wong R., and Lange A. B., 2014 Octopamine modulates a central pattern generator associated with egg-laying in the locust, Locusta migratoria. J Insect Physiol 63: 1–8. 24530620 10.1016/j.jinsphys.2014.02.002

[R127] Wuest M., Eichhorn B., Grimm M. O., Wirth M. P., Ravens U. 2009 Catecholamines relax detrusor through beta 2-adrenoceptors in mouse and beta 3-adrenoceptors in man. J Pharmacol Exp Ther 328: 213–222. 18820136 10.1124/jpet.108.142562

[R128] Yamamoto D., 2007 The neural and genetic substrates of sexual behavior in Drosophila. Adv Genet 59: 39–66. 17888794 10.1016/S0065-2660(07)59002-4

[R129] Yartsev V. V., and Evseeva S. S., 2021 The Male Urogenital System of a Salamander (Amphibia, Caudata). Current Herpetology 40: 10–21.

[R130] Yu J., Zhang Y., Clements K., Chen N. and Griffith L. C., 2025 Genetically-encoded markers for confocal visualization of single dense core vesicles. Commun Biol 8: 383. 40050695 10.1038/s42003-025-07829-y PMC11885565

[R131] Zhang Y., Rozsa M., Liang Y., Bushey D., Wei Z. 2023 Fast and sensitive GCaMP calcium indicators for imaging neural populations. Nature 615: 884–891. 36922596 10.1038/s41586-023-05828-9 PMC10060165

