# Supplementary material for: Two classes of amine/glutamate multi-transmitter neurons innervate Drosophila internal male reproductive organs
Source: bioRxiv. 2025 Jul 28:2025.07.23.666348. Preprint. [Version 1] doi: 10.1101/2025.07.23.666348 (PMC12324174; doi:10.1101/2025.07.23.666348)
Supplement: Supplementary file 1 [file NIHPP2025.07.23.666348v1-supplement-1.pdf]

bioRxiv preprint doi: https://doi.org/10.1101/2025.07.23.666348; this version posted July 28, 2025. The copyright holder for this preprint (which was not certified by peer review) is the author/funder, who has granted bioRxiv a license to display the preprint in perpetuity. It is made available under aCC-BY 4.0 International license.

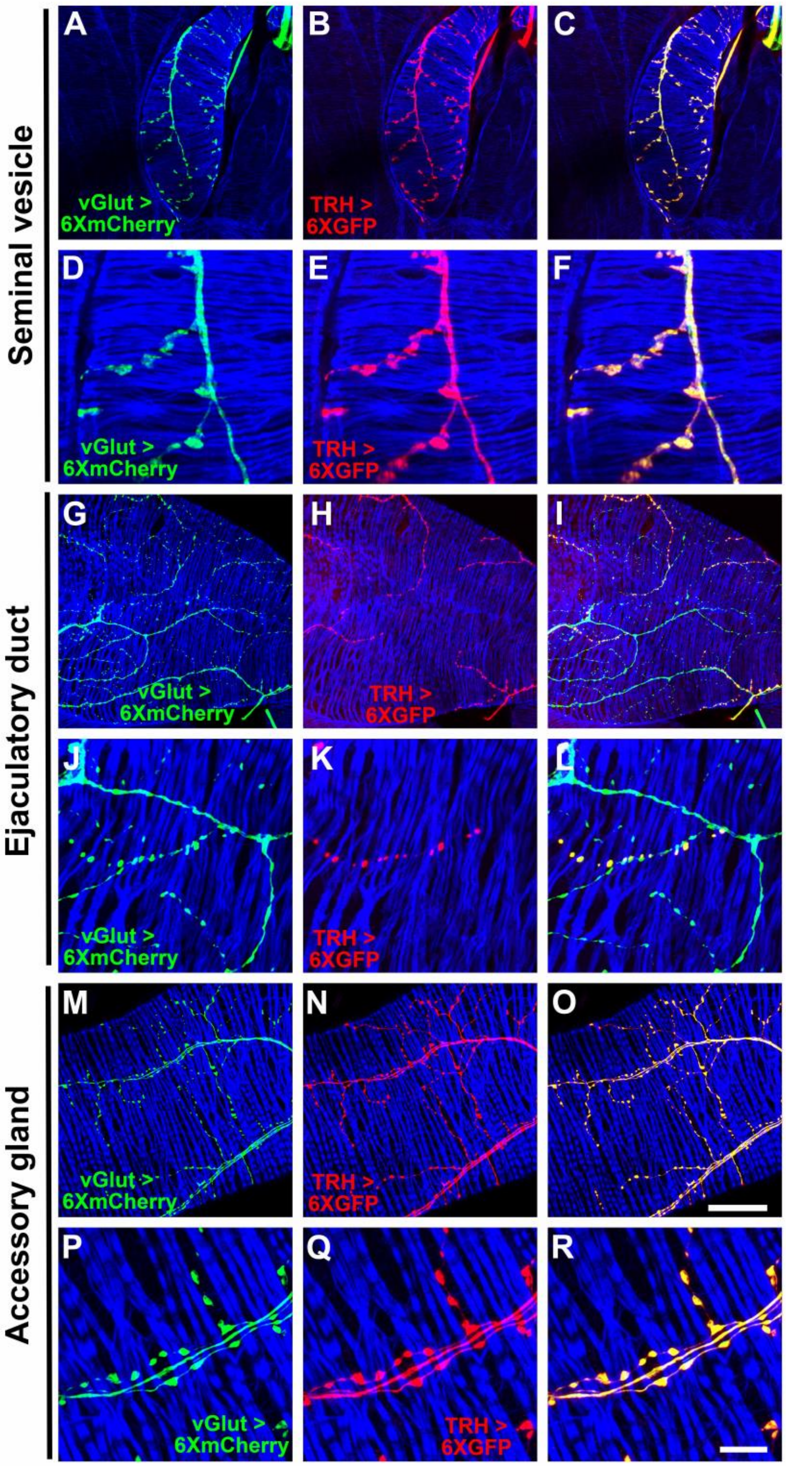

1218

bioRxiv preprint doi: https://doi.org/10.1101/2025.07.23.666348; this version posted July 28, 2025. The copyright holder for this preprint (which was not certified by peer review) is the author/funder, who has granted bioRxiv a license to display the preprint in perpetuity. It is made available under aCC-BY 4.0 International license.

Figure 3S1. Expression of *vGlut-LexA* and *TRH-GAL4* in the Drosophila male reproductive system. A, D, G, J, M, P) *vGlut-LexA*, *LexAop-6XmCherry*; B, E, H, K, N, Q) *TRH-GAL4*, *UAS-6XGFP*. C, F, I, L, O, R) overlay. Scale bars: O-50μm; R-10μm.

bioRxiv preprint doi: https://doi.org/10.1101/2025.07.23.666348; this version posted July 28, 2025. The copyright holder for this preprint (which was not certified by peer review) is the author/funder, who has granted bioRxiv a license to display the preprint in perpetuity. It is made available under aCC-BY 4.0 International license.

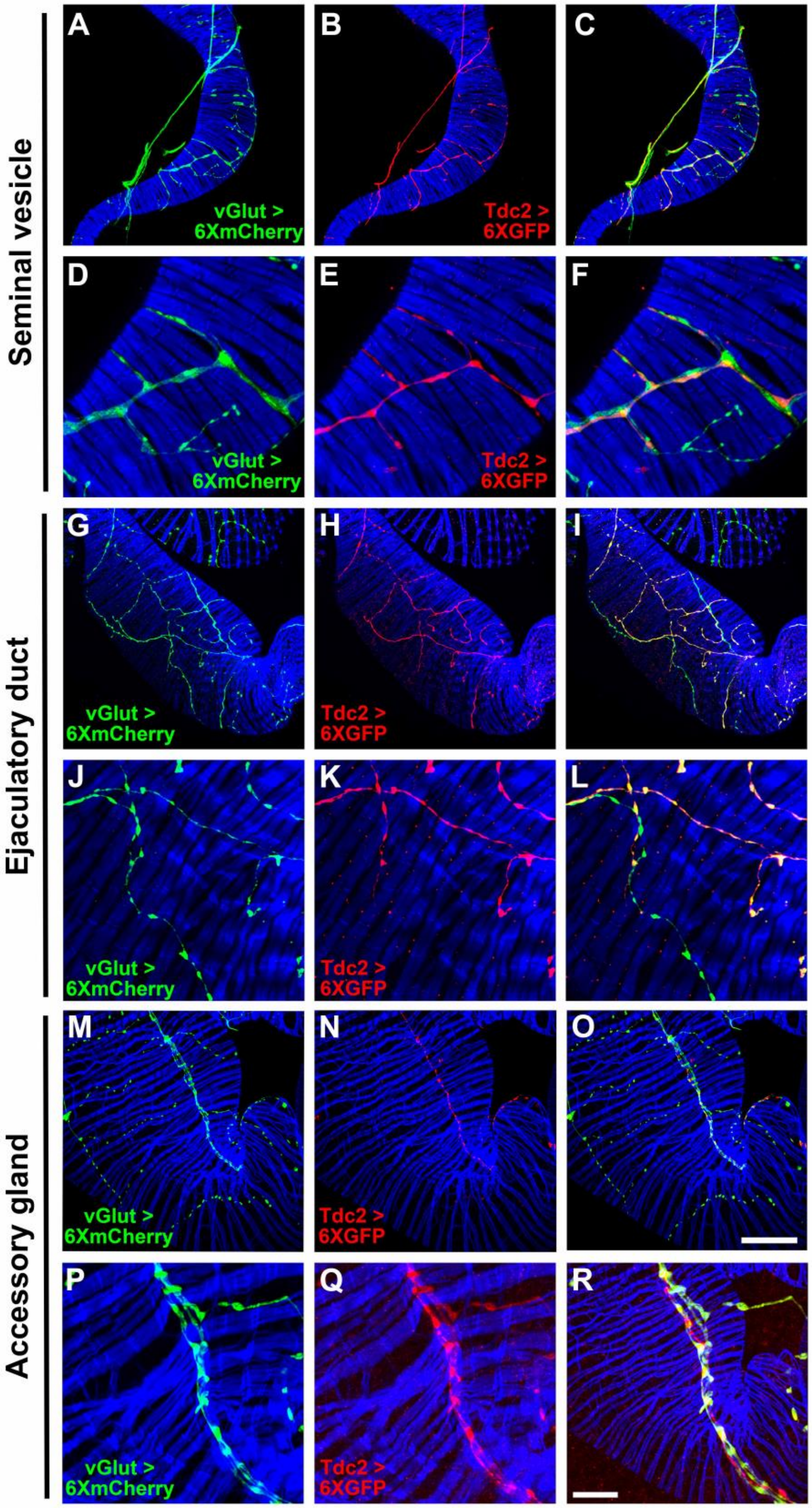

1222

bioRxiv preprint doi: https://doi.org/10.1101/2025.07.23.666348; this version posted July 28, 2025. The copyright holder for this preprint (which was not certified by peer review) is the author/funder, who has granted bioRxiv a license to display the preprint in perpetuity. It is made available under aCC-BY 4.0 International license.

Figure 3S2. Expression of *vGlut-LexA* and *Tdc2-GAL4* in the Drosophila male reproductive system. A, D, G, J, M, P) *vGlut-LexA*; B, E, H, K, N, Q) *Tdc2-GAL4*, *LexAop-6XmCherry*. C, F, I, L, O, R) overlay. Scale bars: O-50μm; R-10μm.

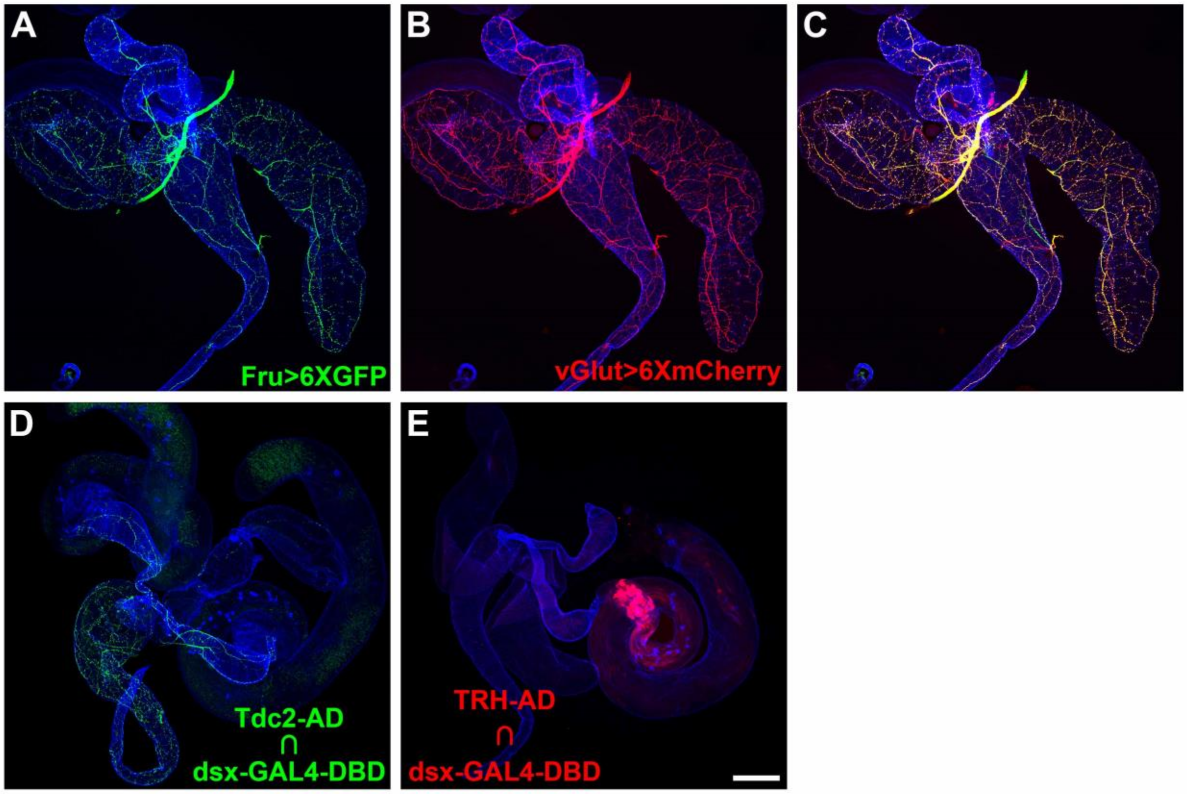

Figure 3S3. Fruitless and Doublesex expression in the Drosophila male reproductive system. A) *fru-GAL4*, *UAS-6XGFP*; B) *vGlut-LexA*, *LexAop-6XmCherry*; C) overlay; D) Tdc2-AD ∩ dsx-GAL4; E) TRH-AD ∩ dsx-GAL4. Scale bar: 200μm.

bioRxiv preprint doi: https://doi.org/10.1101/2025.07.23.666348; this version posted July 28, 2025. The copyright holder for this preprint (which was not certified by peer review) is the author/funder, who has granted bioRxiv a license to display the preprint in perpetuity. It is made available under aCC-BY 4.0 International license.

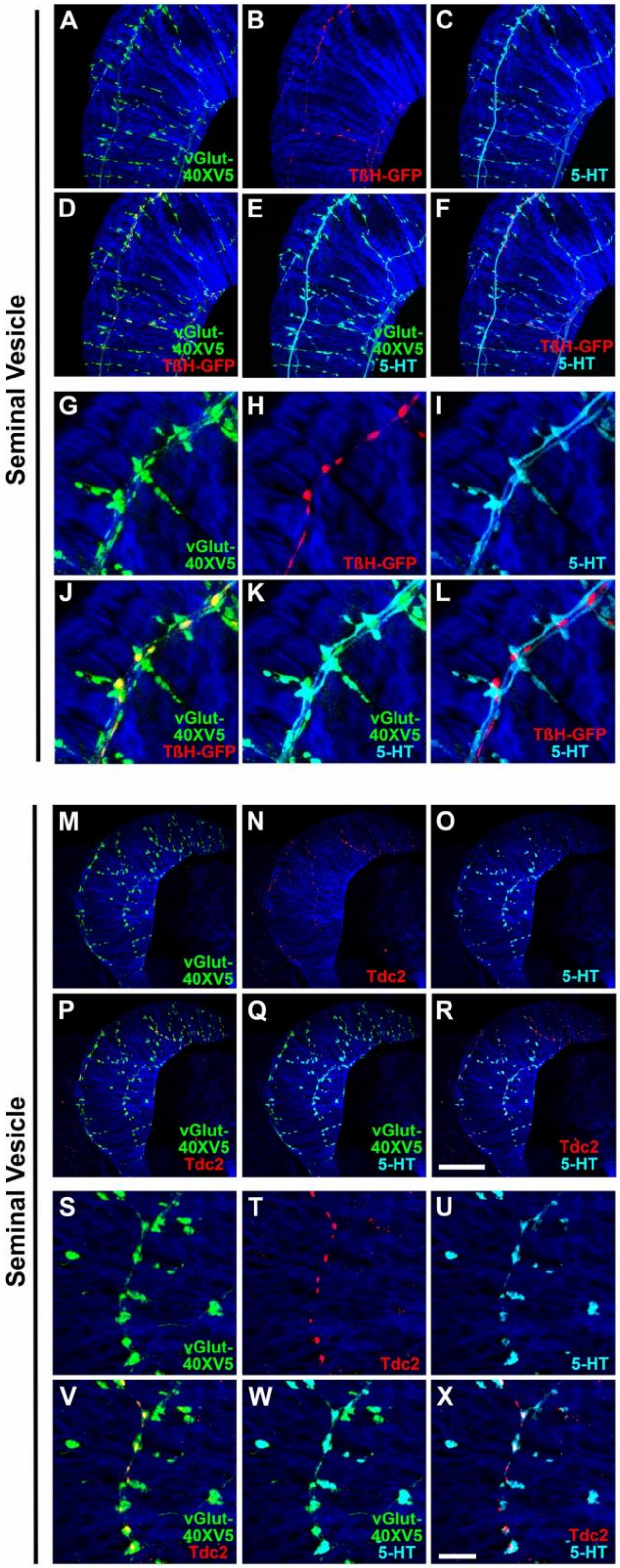

1236

bioRxiv preprint doi: https://doi.org/10.1101/2025.07.23.666348; this version posted July 28, 2025. The copyright holder for this preprint (which was not certified by peer review) is the author/funder, who has granted bioRxiv a license to display the preprint in perpetuity. It is made available under aCC-BY 4.0 International license.

Figure 4S1. Expression of vGlut, TβH-GFP, Tdc2, and 5-HT in the SV of the Drosophila male reproductive system. A, G) vGlut-40XV5; B, H) TβH-GFP; C, I) 5-HT; D, J) vGlut-40XV5, TβH-GFP overlay; E, K) vGlut-40XV5, 5-HT overlay; F, L) TβH-GFP, 5-HT overlay; M, S) vGlut-40XV5; N, T) Tdc2; O, U) 5-HT; P, V) vGlut-40XV5, Tdc2 overlay; Q, W) vGlut-40XV5, 5-HT overlay; R, X) Tdc2, 5-HT overlay. Scale bars: R-50μm; X-10μm.

bioRxiv preprint doi: https://doi.org/10.1101/2025.07.23.666348; this version posted July 28, 2025. The copyright holder for this preprint (which was not certified by peer review) is the author/funder, who has granted bioRxiv a license to display the preprint in perpetuity. It is made available under aCC-BY 4.0 International license.

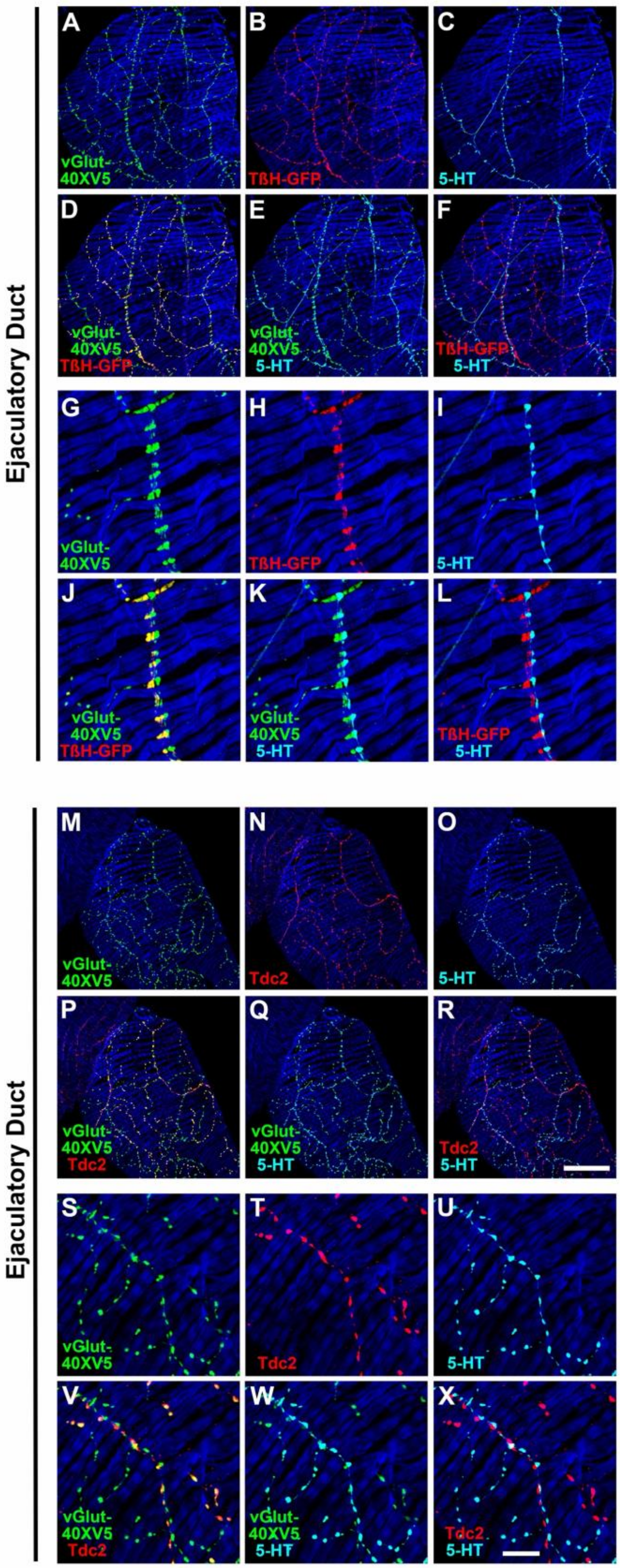

1243

bioRxiv preprint doi: https://doi.org/10.1101/2025.07.23.666348; this version posted July 28, 2025. The copyright holder for this preprint (which was not certified by peer review) is the author/funder, who has granted bioRxiv a license to display the preprint in perpetuity. It is made available under aCC-BY 4.0 International license.

Figure 4S2. Expression of vGlut, TβH-GFP, Tdc2, and 5-HT in the ED of the Drosophila male reproductive system. A, G) vGlut-40XV5; B, H) TβH-GFP; C, I) 5-HT; D, J) vGlut-40XV5, TβH-GFP overlay; E, K) vGlut-40XV5, 5-HT overlay; F, L) TβH-GFP, 5-HT overlay; M, S) vGlut-40XV5; N, T) Tdc2; O, U) 5-HT; P, V) vGlut-40XV5, Tdc2 overlay; Q, W) vGlut-40XV5, 5-HT overlay; R, X) Tdc2, 5-HT overlay. Scale bars: R-50μm; X-10μm.

bioRxiv preprint doi: https://doi.org/10.1101/2025.07.23.666348; this version posted July 28, 2025. The copyright holder for this preprint (which was not certified by peer review) is the author/funder, who has granted bioRxiv a license to display the preprint in perpetuity. It is made available under aCC-BY 4.0 International license.

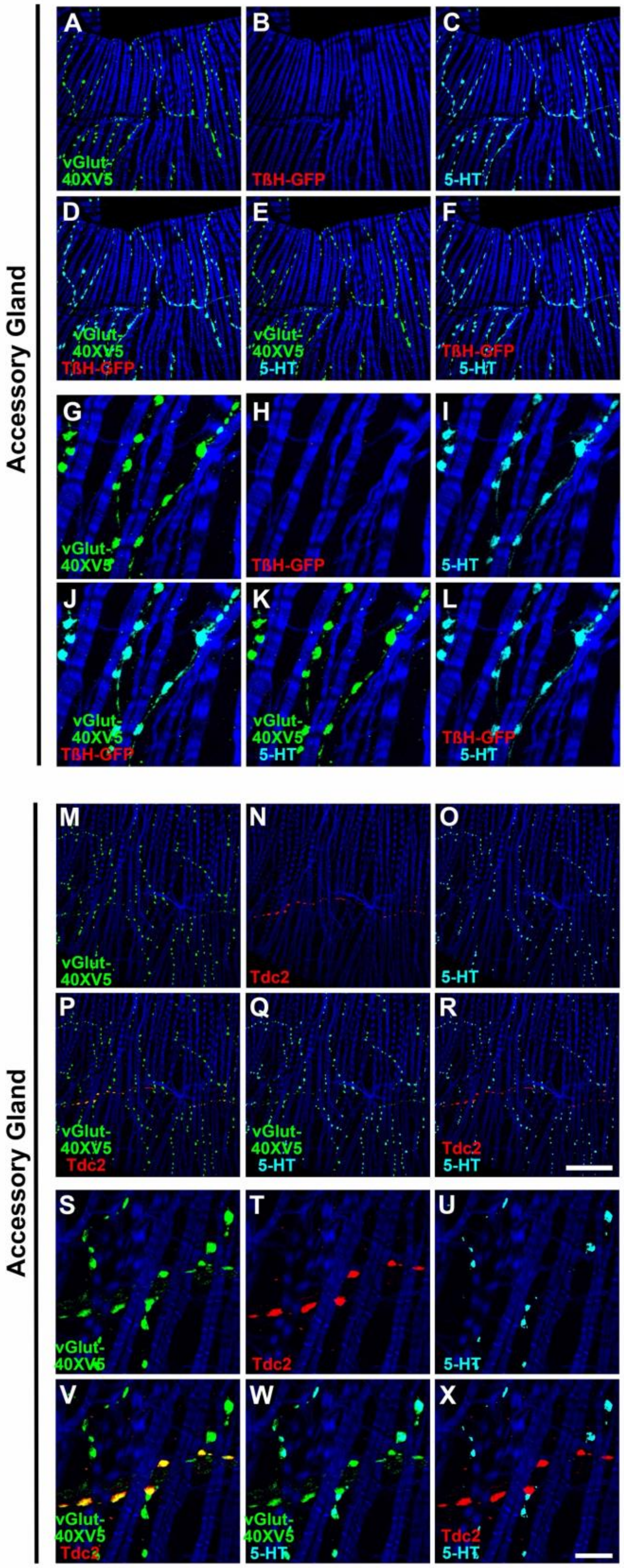

1250

bioRxiv preprint doi: https://doi.org/10.1101/2025.07.23.666348; this version posted July 28, 2025. The copyright holder for this preprint (which was not certified by peer review) is the author/funder, who has granted bioRxiv a license to display the preprint in perpetuity. It is made available under aCC-BY 4.0 International license.

Figure 4S3. Expression of vGlut, TβH-GFP, Tdc2, and 5-HT in the AG of the Drosophila male reproductive system. A, G) vGlut-40XV5; B, H) TβH-GFP; C, I) 5-HT; D, J) vGlut-40XV5, TβH-GFP overlay; E, K) vGlut-40XV5, 5-HT overlay; F, L) TβH-GFP, 5-HT overlay; M, S) vGlut-40XV5; N, T) Tdc2; O, U) 5-HT; P, V) vGlut-40XV5, Tdc2 overlay; Q, W) vGlut-40XV5, 5-HT overlay; R, X) Tdc2, 5-HT overlay. Scale bars: R-50μm; X-10μm.

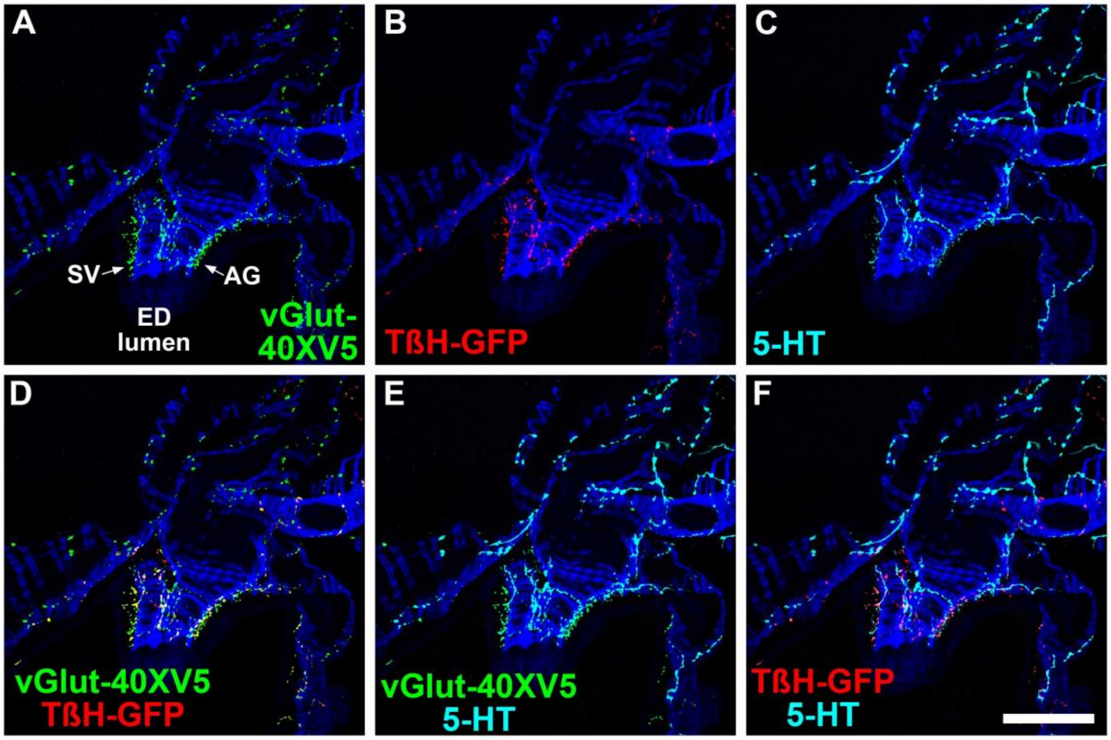

Figure 4S4. Expression of vGlut, TβH-GFP, and 5-HT at the junction of the SV and AGs with the ED of the Drosophila male reproductive system. A) vGlut-40XV5; B) TβH-GFP; C) 5-HT; D) vGlut-40XV5, TβH-GFP overlay; E) vGlut-40XV5, 5-HT overlay; F) TβH-GFP, 5-HT overlay. A) vGlut-40XV5; B) Tdc2; C) 5-HT; D) vGlut-40XV5, Tdc2 overlay; E) vGlut-40XV5, 5-HT overlay; F) Tdc2, 5-HT overlay. Scale bar: 50μm.

bioRxiv preprint doi: https://doi.org/10.1101/2025.07.23.666348; this version posted July 28, 2025. The copyright holder for this preprint (which was not certified by peer review) is the author/funder, who has granted bioRxiv a license to display the preprint in perpetuity. It is made available under aCC-BY 4.0 International license.

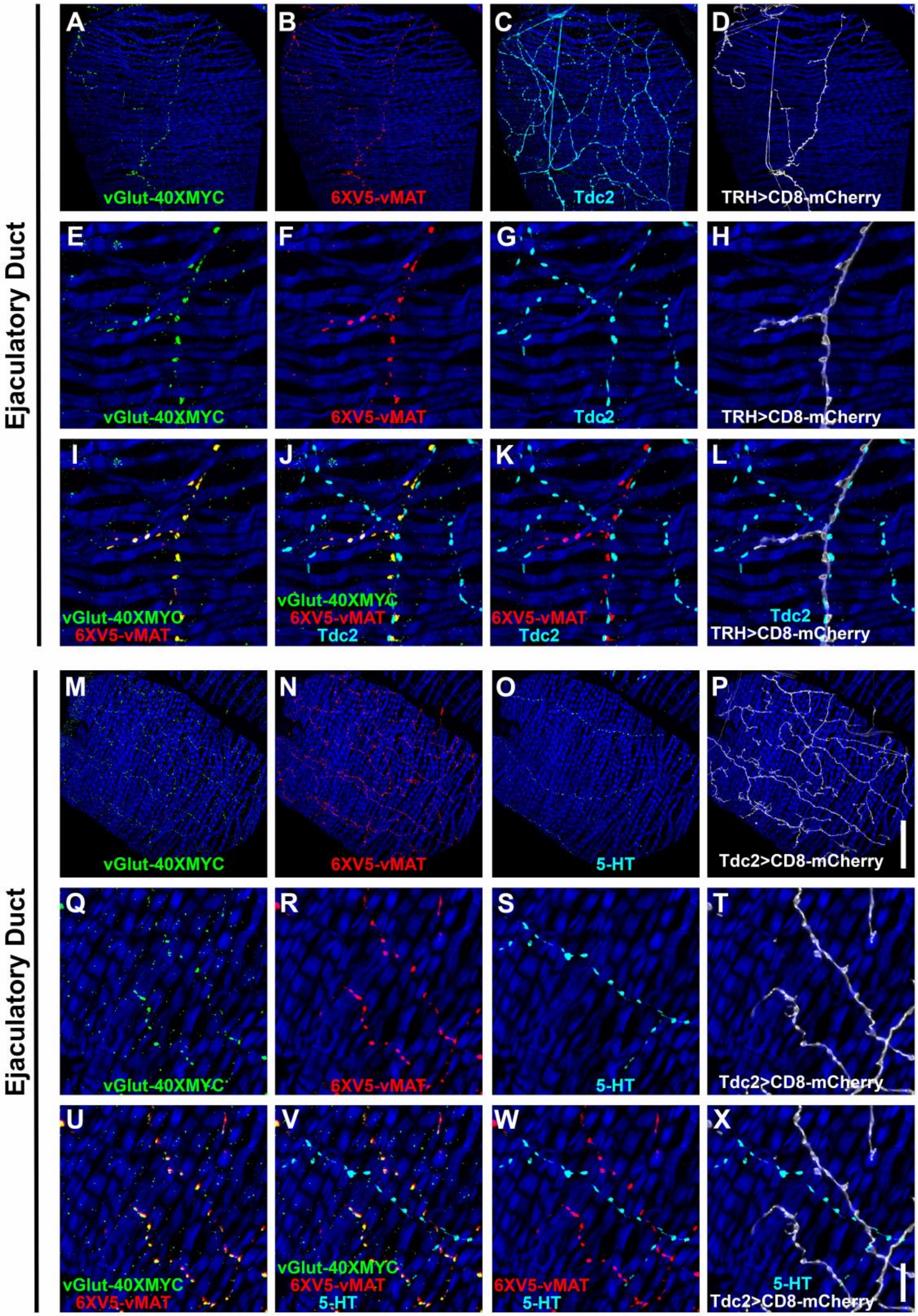

1275

bioRxiv preprint doi: https://doi.org/10.1101/2025.07.23.666348; this version posted July 28, 2025. The copyright holder for this preprint (which was not certified by peer review) is the author/funder, who has granted bioRxiv a license to display the preprint in perpetuity. It is made available under aCC-BY 4.0 International license.

Figure 5S1. Co-conditional expression of vGlut-40XMYC and 6XV5-vMAT in the ED of serotonergic (TRH) or octopaminergic (Tdc2) neurons of the Drosophila male reproductive system. A-D) 63X. A) vGlut-40XMYC; B) 6XV5-vMAT; C) Tdc2; D) TRH>CD8-mCherry. E-L) 63X zoom 4X. E) vGlut-40XMYC; F) 6XV5-vMAT; G) Tdc2; H) TRH>CD8-mCherry; I) vGlut-40XMYC, 6XV5-vMAT overlay; J) vGlut-40XMYC, 6XV5-vMAT, Tdc2 overlay; K) 6XV5-vMAT, Tdc2 overlay; L) Tdc2, TRH>CD8-mCherry overlay. M-P) 63X. M) vGlut-40XMYC; N) 6XV5-vMAT; O) 5-HT; P) Tdc2>CD8-mCherry. Q-X) 63X zoom 4X. Q) vGlut-40XMYC; R) 6XV5-vMAT; S) 5-HT; T) Tdc2>CD8-mCherry; U) vGlut-40XMYC, 6XV5-vMAT overlay; V) vGlut-40XMYC, 6XV5-vMAT, 5-HT overlay; W) 6XV5-vMAT, 5-HT overlay; X) Tdc2, Tdc2>CD8-mCherry overlay. Scale bars: P-50µm; X-10µm.

bioRxiv preprint doi: https://doi.org/10.1101/2025.07.23.666348; this version posted July 28, 2025. The copyright holder for this preprint (which was not certified by peer review) is the author/funder, who has granted bioRxiv a license to display the preprint in perpetuity. It is made available under aCC-BY 4.0 International license.

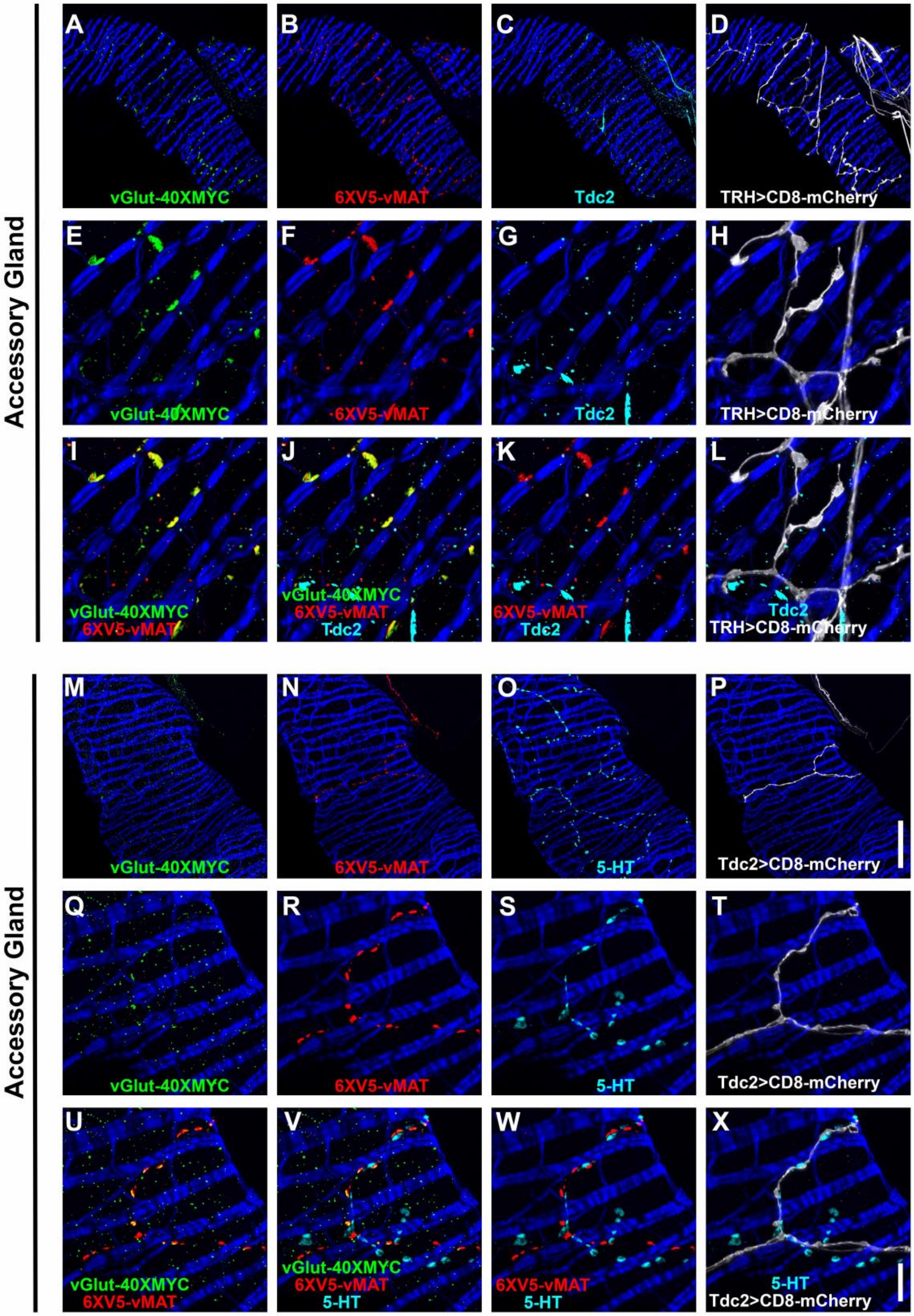

1287

bioRxiv preprint doi: https://doi.org/10.1101/2025.07.23.666348; this version posted July 28, 2025. The copyright holder for this preprint (which was not certified by peer review) is the author/funder, who has granted bioRxiv a license to display the preprint in perpetuity. It is made available under aCC-BY 4.0 International license.

Figure 5S2. Co-conditional expression of vGlut-40XMYC and 6XV5-vMAT in the AG of serotonergic (TRH) or octopaminergic (Tdc2) neurons of the Drosophila male reproductive system. A-D) 63X. A) vGlut-40XMYC; B) 6XV5-vMAT; C) Tdc2; D) TRH>CD8-mCherry. E-L) 63X zoom 4X. E) vGlut-40XMYC; F) 6XV5-vMAT; G) Tdc2; H) TRH>CD8-mCherry; I) vGlut-40XMYC, 6XV5-vMAT overlay; J) vGlut-40XMYC, 6XV5-vMAT, Tdc2 overlay; K) 6XV5-vMAT, Tdc2 overlay; L) Tdc2, TRH>CD8-mCherry overlay. M-P) 63X. M) vGlut-40XMYC; N) 6XV5-vMAT; O) 5-HT; P) Tdc2>CD8-mCherry. Q-X) 63X zoom 4X. Q) vGlut-40XMYC; R) 6XV5-vMAT; S) 5-HT; T) Tdc2>CD8-mCherry; U) vGlut-40XMYC, 6XV5-vMAT overlay; V) vGlut-40XMYC, 6XV5-vMAT, 5-HT overlay; W) 6XV5-vMAT, 5-HT overlay; X) Tdc2, Tdc2>CD8-mCherry overlay. Scale bars: P-50μm; X-10μm.

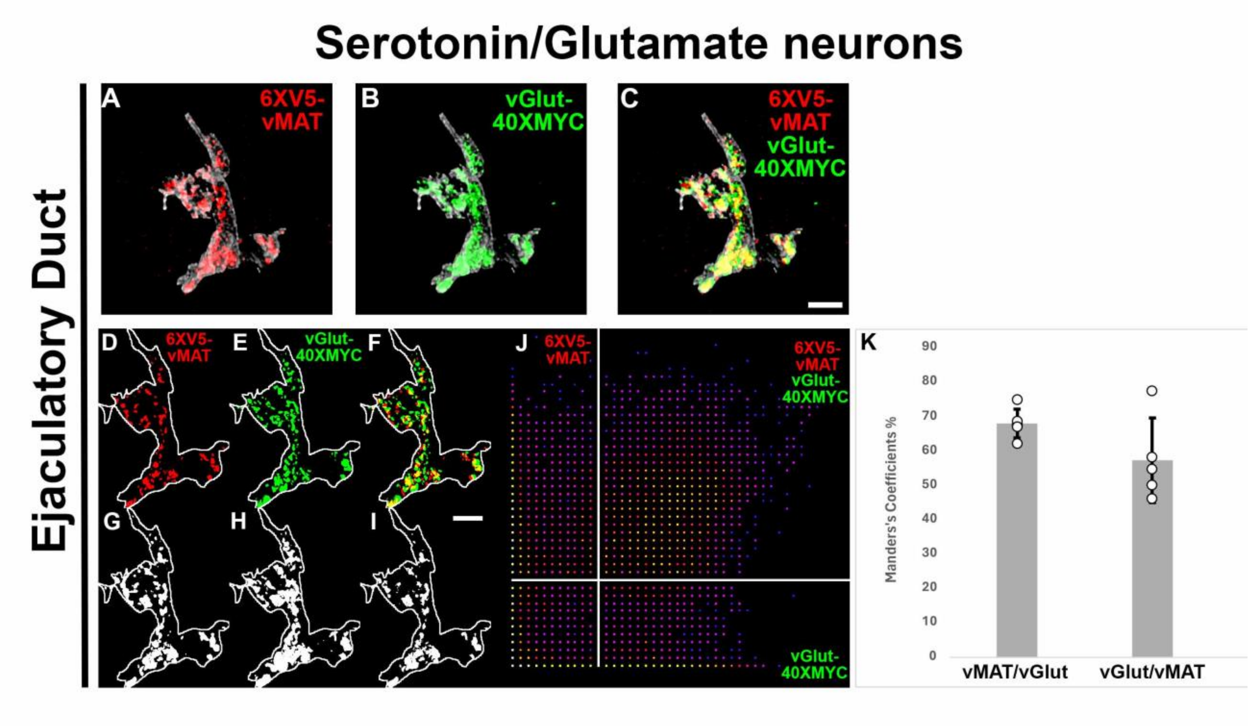

Figure 5S3. Co-conditional expression of vGlut-40XMYC and 6XV5-vMAT in serotonergic neurons of the Drosophila ED using expansion microscopy. A, D) 6XV5-vMAT; B, E) vGlut-40XMYC; C, F) overlay. A-C) 6XV5-vMAT and vGlut-40XMYC expression above the threshold intensity values. D-F) Complete 6XV5-vMAT and vGlut-40XMYC expression. G) Mander's plot of 6XV5-vMAT and vGlut-40XMYC. Vertical line indicates intensity threshold value for 6XV5-vMAT. Horizontal line indicates intensity threshold value for vGlut-40XMYC. H) Mander's coefficients. Grayscale signal in A-C) denotes the CD8-mCherry plasma membrane marker. Scale bars: 500nm. Scale bars have been corrected by 10X to reflect the empirically determined 10.5X expansion factor.

bioRxiv preprint doi: https://doi.org/10.1101/2025.07.23.666348; this version posted July 28, 2025. The copyright holder for this preprint (which was not certified by peer review) is the author/funder, who has granted bioRxiv a license to display the preprint in perpetuity. It is made available under aCC-BY 4.0 International license.

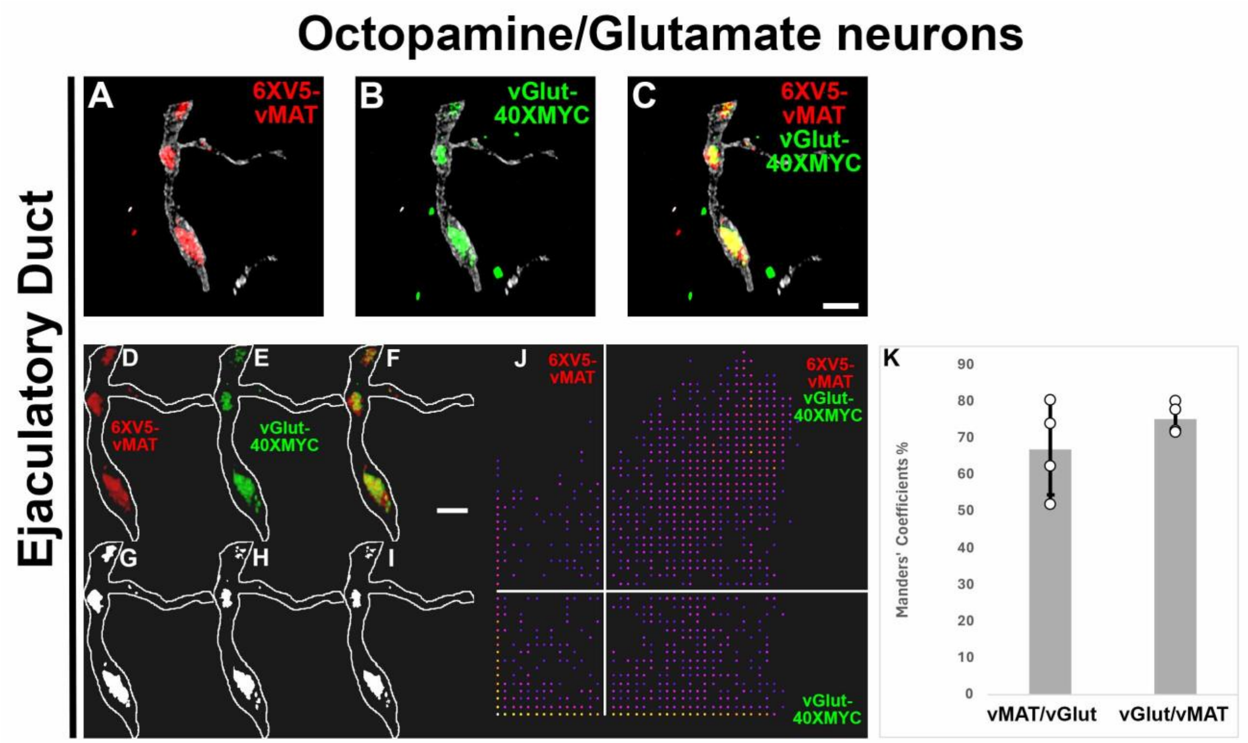

Figure 5S4. Co-conditional expression of vGlut-40XMYC and 6XV5-vMAT in octopaminergic neurons of the Drosophila ED using expansion microscopy. A, D) 6XV5-vMAT; B, E) vGlut-40XMYC; C, F) overlay. A-C) 6XV5-vMAT and vGlut-40XMYC expression above the threshold intensity values. D-F) Complete 6XV5-vMAT and vGlut-40XMYC expression. G) Mander's plot of 6XV5-vMAT and vGlut-40XMYC. Vertical line indicates intensity threshold value for 6XV5-vMAT. Horizontal line indicates intensity threshold value for vGlut-40XMYC. H) Mander's coefficients. Grayscale signal in A-C) denotes the CD8-mCherry plasma membrane marker. Scale bars: 500nm. Scale bars have been corrected by 10X to reflect the empirically determined 10.5X expansion factor.

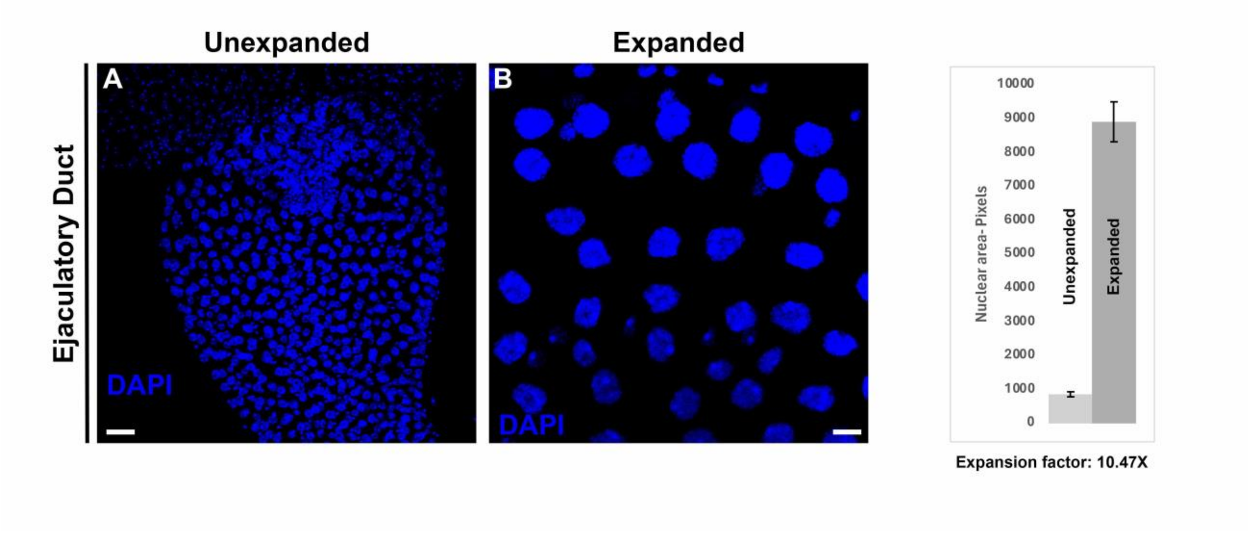

bioRxiv preprint doi: https://doi.org/10.1101/2025.07.23.666348; this version posted July 28, 2025. The copyright holder for this preprint (which was not certified by peer review) is the author/funder, who has granted bioRxiv a license to display the preprint in perpetuity. It is made available under aCC-BY 4.0 International license.

Figure 5S5. Nuclei of unexpanded and expanded EDs. A) DAPI-stained nuclei from an unexpanded ED. B) DAPI-stained nuclei of an expanded ED. C) Bar graph plot of nuclei area (mean ± SE-black bars). For the experiment shown the calculated expansion factor (post-ExM nuclear area/pre-ExM nuclear area) was 10.47, p-value < 0.00001. Fij/ImageJi open-source software was used to segment images and calculate nuclear area. Scale bars: 25um.

bioRxiv preprint doi: https://doi.org/10.1101/2025.07.23.666348; this version posted July 28, 2025. The copyright holder for this preprint (which was not certified by peer review) is the author/funder, who has granted bioRxiv a license to display the preprint in perpetuity. It is made available under aCC-BY 4.0 International license.

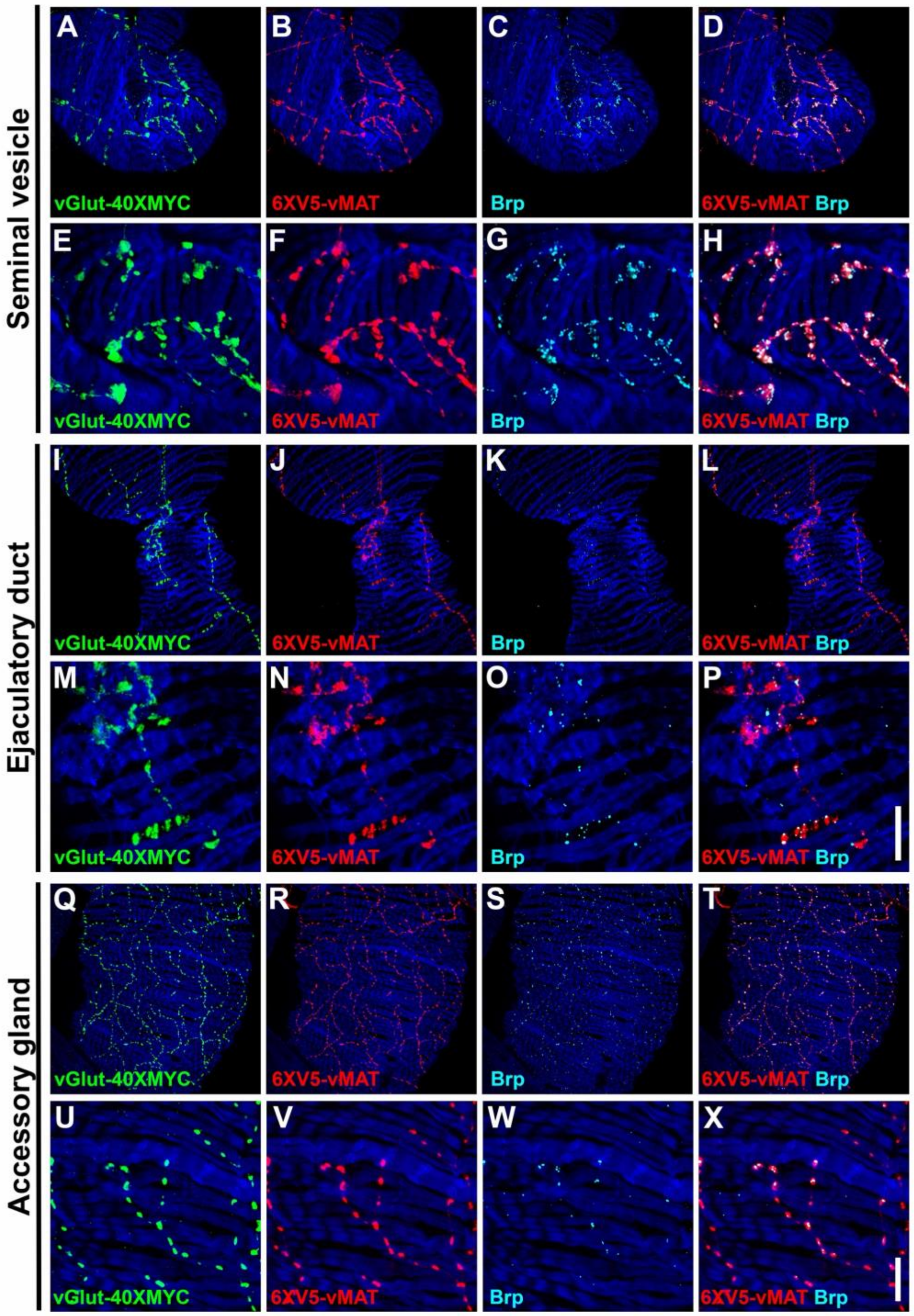

1340

bioRxiv preprint doi: https://doi.org/10.1101/2025.07.23.666348; this version posted July 28, 2025. The copyright holder for this preprint (which was not certified by peer review) is the author/funder, who has granted bioRxiv a license to display the preprint in perpetuity. It is made available under aCC-BY 4.0 International license.

Figure 6S1. Expression of the active zone marker Brp in combination with vGlut-40XMYC and 6XV5-vMAT in the SV, ED, and AG of the Drosophila male reproductive system. A-H) SV. A) vGlut-40XMYC; B) 6XV5-vMAT; C) Brp; D) 6XV5-vMAT, Brp overlay; E) vGlut-40XMYC; F) 6XV5-vMAT; G) Brp; H) 6XV5-vMAT, Brp overlay. I-P) ED. I) vGlut-40XMYC; J) 6XV5-vMAT; K) Brp; L) 6XV5-vMAT, Brp; M) vGlut-40XMYC; N) 6XV5-vMAT; O) Brp; P) 6XV5-vMAT, Brp overlay. I-P) AG. Q) vGlut-40XMYC; R) 6XV5-vMAT; S) Brp; T) 6XV5-vMAT, Brp; U) vGlut-40XMYC; V) 6XV5-vMAT; W) Brp; X) 6XV5-vMAT, Brp overlay. Scale bars: P-50μm; X-10μm.

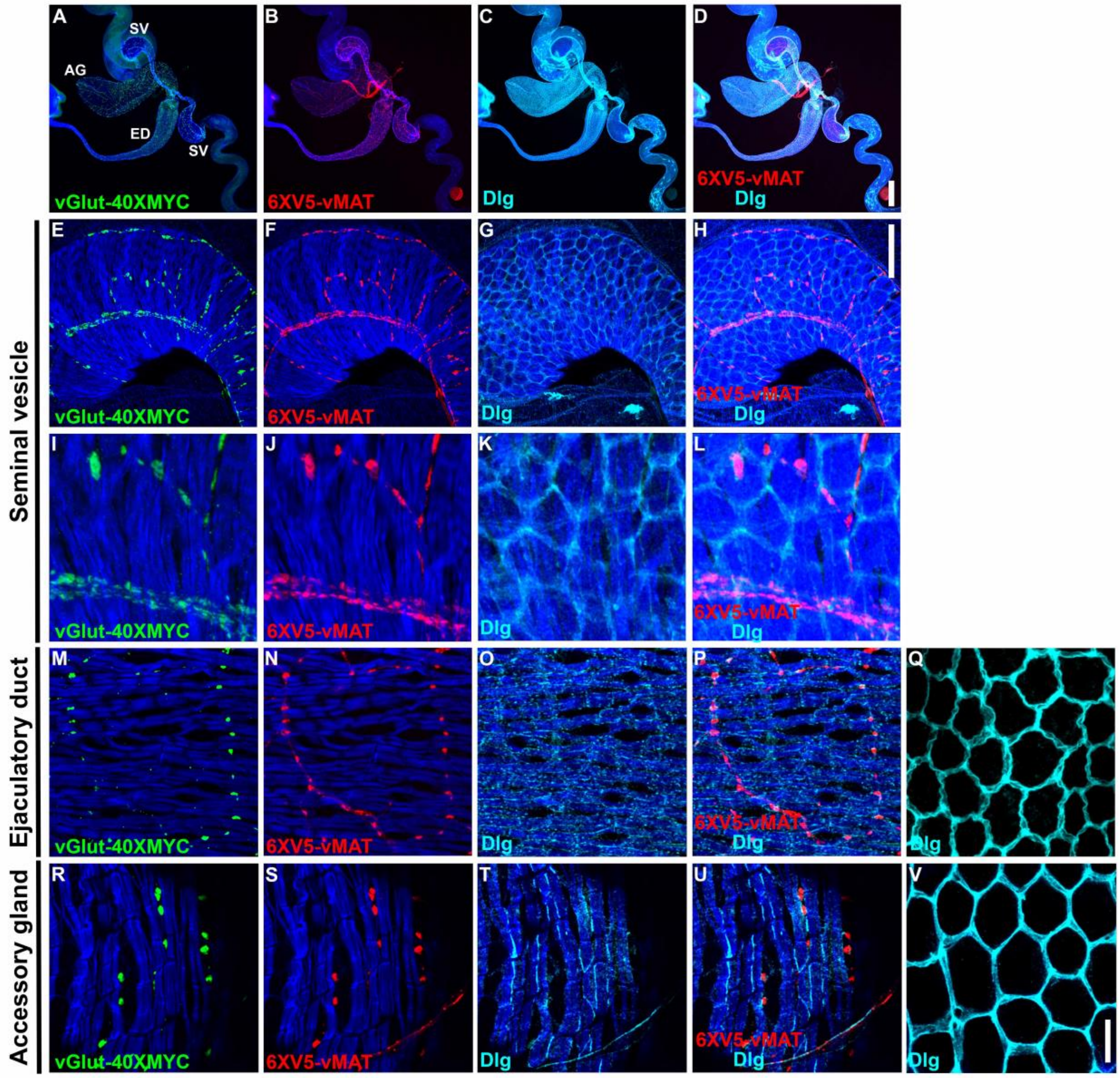

Figure 6S2. Expression of the post-synaptic density marker Dlg in combination with vGlut-40XMYC and 6XV5-vMAT in the SV, ED, and AG of the Drosophila male reproductive system. A-D) Complete reproductive system. A) vGlut-40XMYC; B) 6XV5-

bioRxiv preprint doi: https://doi.org/10.1101/2025.07.23.666348; this version posted July 28, 2025. The copyright holder for this preprint (which was not certified by peer review) is the author/funder, who has granted bioRxiv a license to display the preprint in perpetuity. It is made available under aCC-BY 4.0 International license.

vMAT; C) Dlg; D) 6XV5-vMAT, Dlg overlay; E-L) SV. E) vGlut-40XMYC; F) 6XV5-vMAT; G) Dlg; H) 6XV5-vMAT, Dlg overlay; I) vGlut-40XMYC; J) 6XV5-vMAT; G) Dlg; H) 6XV5-vMAT, Dlg overlay. I-P) ED. I) vGlut-40XMYC; K) 6XV5-vMAT; L) Dlg; M-P) ED muscle surface. M) vGlut-40XMYC; N) 6XV5-vMAT; O) Dlg; P) 6XV5-vMAT, Dlg overlay Q) Dlg in the ED epithelial cells. R-U) AG muscle surface. R) vGlut-40XMYC; S) 6XV5-vMAT; T) Dlg; U) 6XV5-vMAT, Dlg overlay; V) Dlg in AG epithelial cells. Scale bars: D-200μm; H-50μm; V-10μm.

bioRxiv preprint doi: https://doi.org/10.1101/2025.07.23.666348; this version posted July 28, 2025. The copyright holder for this preprint (which was not certified by peer review) is the author/funder, who has granted bioRxiv a license to display the preprint in perpetuity. It is made available under aCC-BY 4.0 International license.

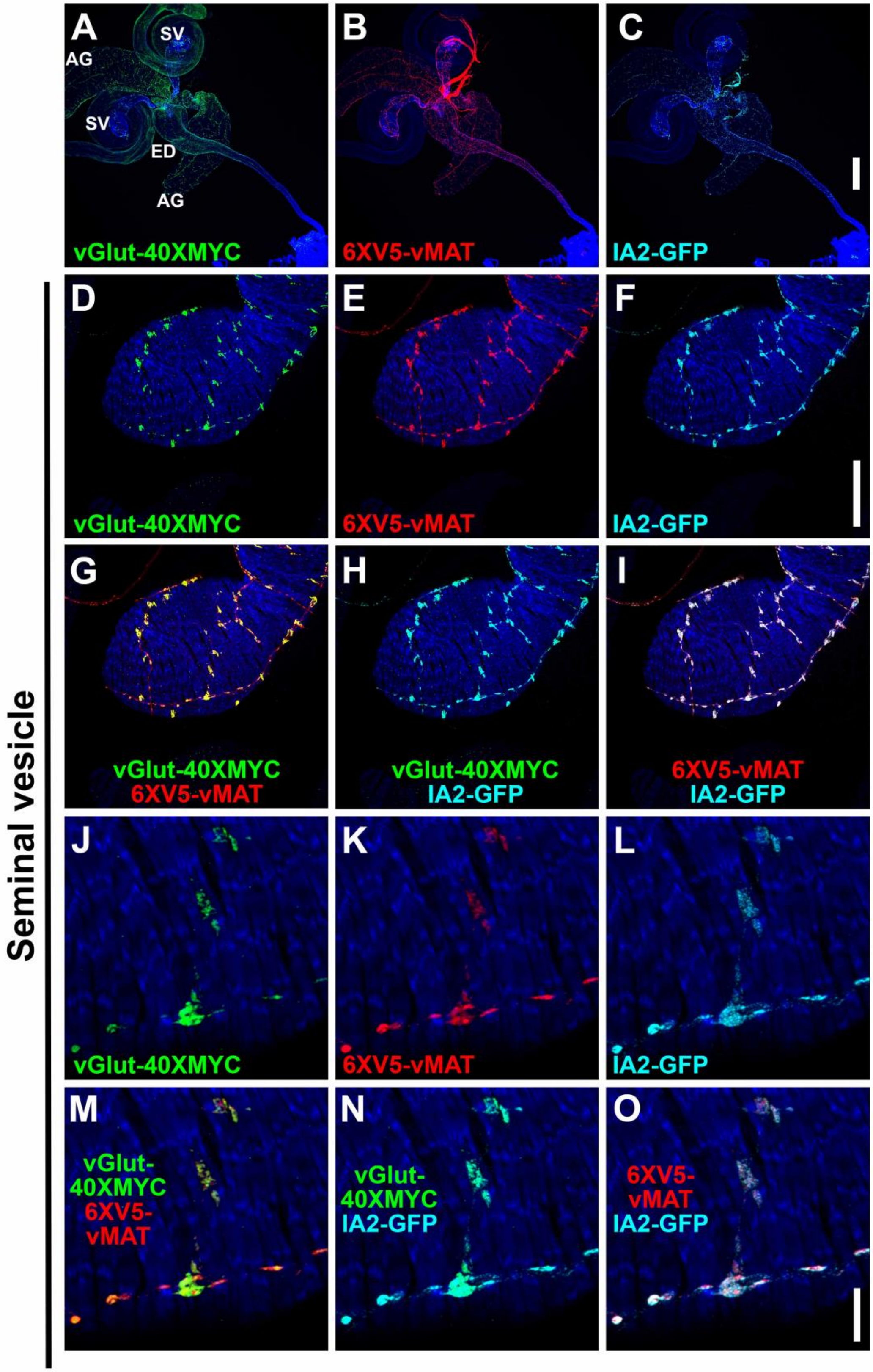

1361

bioRxiv preprint doi: https://doi.org/10.1101/2025.07.23.666348; this version posted July 28, 2025. The copyright holder for this preprint (which was not certified by peer review) is the author/funder, who has granted bioRxiv a license to display the preprint in perpetuity. It is made available under aCC-BY 4.0 International license.

Figure 6S3. Expression of the large core dense vesicle marker IA2-GFP in combination with vGlut-40XMYC and 6XV5-vMAT in the SV of the Drosophila male reproductive system. A-C) Complete reproductive system. A) vGlut-40XMYC; B) 6XV5-vMAT; C) IA2-GFP; D-O) SV. D, J) vGlut-40XMYC; E, K) 6XV5-vMAT; F, L) IA2-GFP; G, M) vGlut-40XMYC, 6XV5-vMAT overlay; H, N) vGlut-40XMYC, IA2-GFP overlay; I, O) 6XV5-vMAT, IA2 overlay. Scale bars: C-200μm; F-50μm; O-10μm.

bioRxiv preprint doi: https://doi.org/10.1101/2025.07.23.666348; this version posted July 28, 2025. The copyright holder for this preprint (which was not certified by peer review) is the author/funder, who has granted bioRxiv a license to display the preprint in perpetuity. It is made available under aCC-BY 4.0 International license.

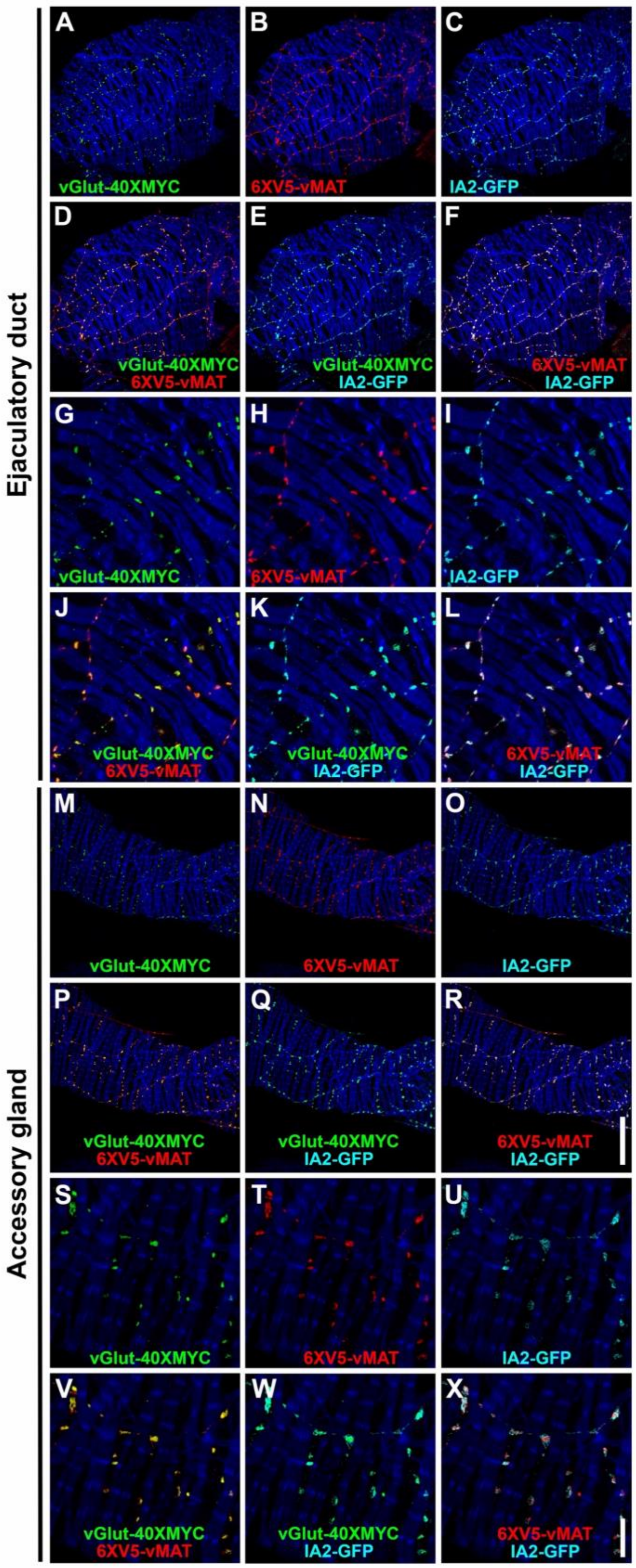

1368

bioRxiv preprint doi: https://doi.org/10.1101/2025.07.23.666348; this version posted July 28, 2025. The copyright holder for this preprint (which was not certified by peer review) is the author/funder, who has granted bioRxiv a license to display the preprint in perpetuity. It is made available under aCC-BY 4.0 International license.

Figure 6S4. Expression of the large core dense vesicle marker IA2-GFP in combination with vGlut-40XMYC and 6XV5-vMAT in the ED and AGs of the Drosophila male reproductive system. A-L) ED. A, G) vGlut-40XMYC; B, H) 6XV5-vMAT; C, I) IA2-GFP; D, J) vGlut-40XMYC, 6XV5-vMAT overlay; E, K) vGlut-40XMYC, IA2-GFP overlay; F, L) 6XV5-vMAT, IA2 overlay. M-X) AG. M, S) vGlut-40XMYC; N, T) 6XV5-vMAT; O, U) IA2-GFP; P, V) vGlut-40XMYC, 6XV5-vMAT overlay; Q, W) vGlut-40XMYC, IA2-GFP overlay; R, X) 6XV5-vMAT, IA2 overlay. Scale bars: R-50μm; X-10μm.

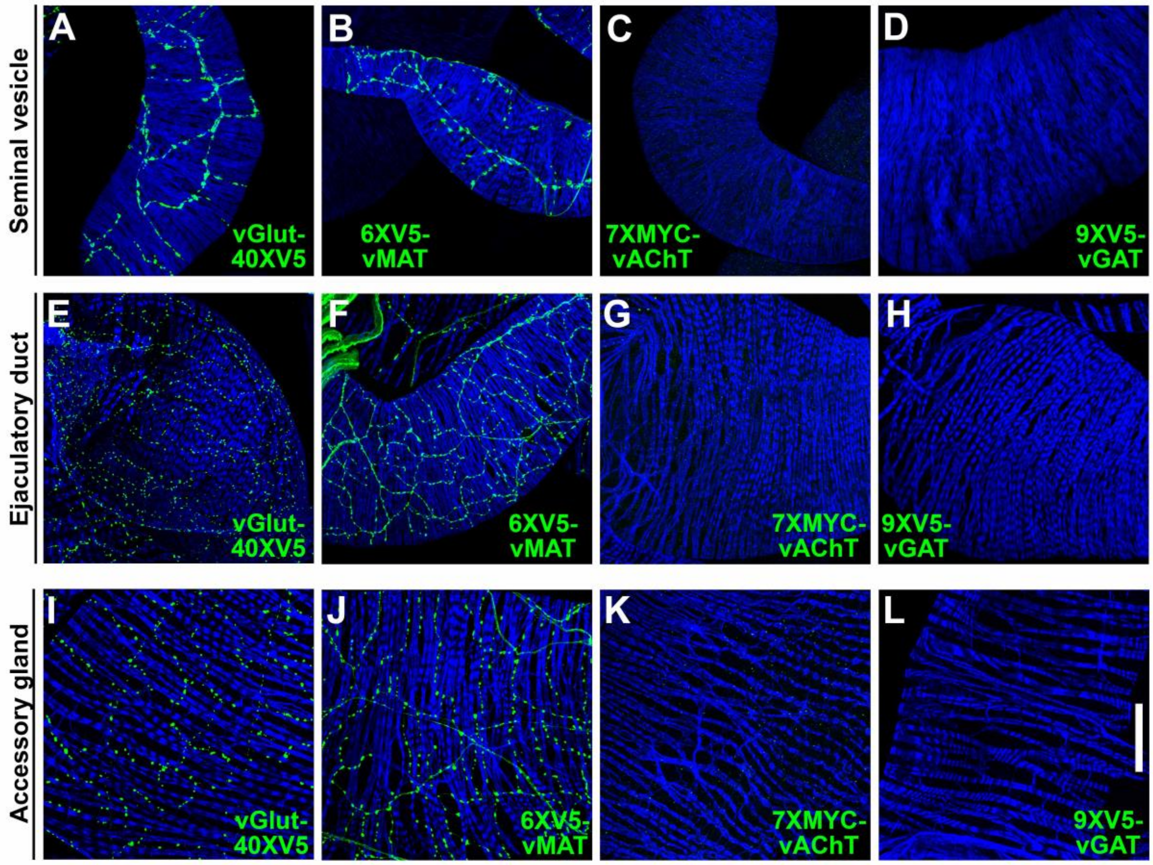

Figure 6S5. Expression of vGlut-40XMYC, 6XV5-vMAT, 7XMYC-vAChT, and 9XV5-vGAT in the SV, ED, and AG of the Drosophila male reproductive system. A-D) SV. A) vGlut-40XV5; B) 6XV5-vMAT; C) 7XMYC-vAChT; D) 9XV5-vGAT. E-H) ED. E) vGlut-40XV5; F) 6XV5-vMAT; G) 7XMYC-vAChT; H) 9XV5-vGAT. I-L) AG. I) vGlut-40XV5; J) 6XV5-vMAT; K) 7XMYC-vAChT; L) 9XV5-vGAT. Scale bar: 50μm.

bioRxiv preprint doi: https://doi.org/10.1101/2025.07.23.666348; this version posted July 28, 2025. The copyright holder for this preprint (which was not certified by peer review) is the author/funder, who has granted bioRxiv a license to display the preprint in perpetuity. It is made available under aCC-BY 4.0 International license.

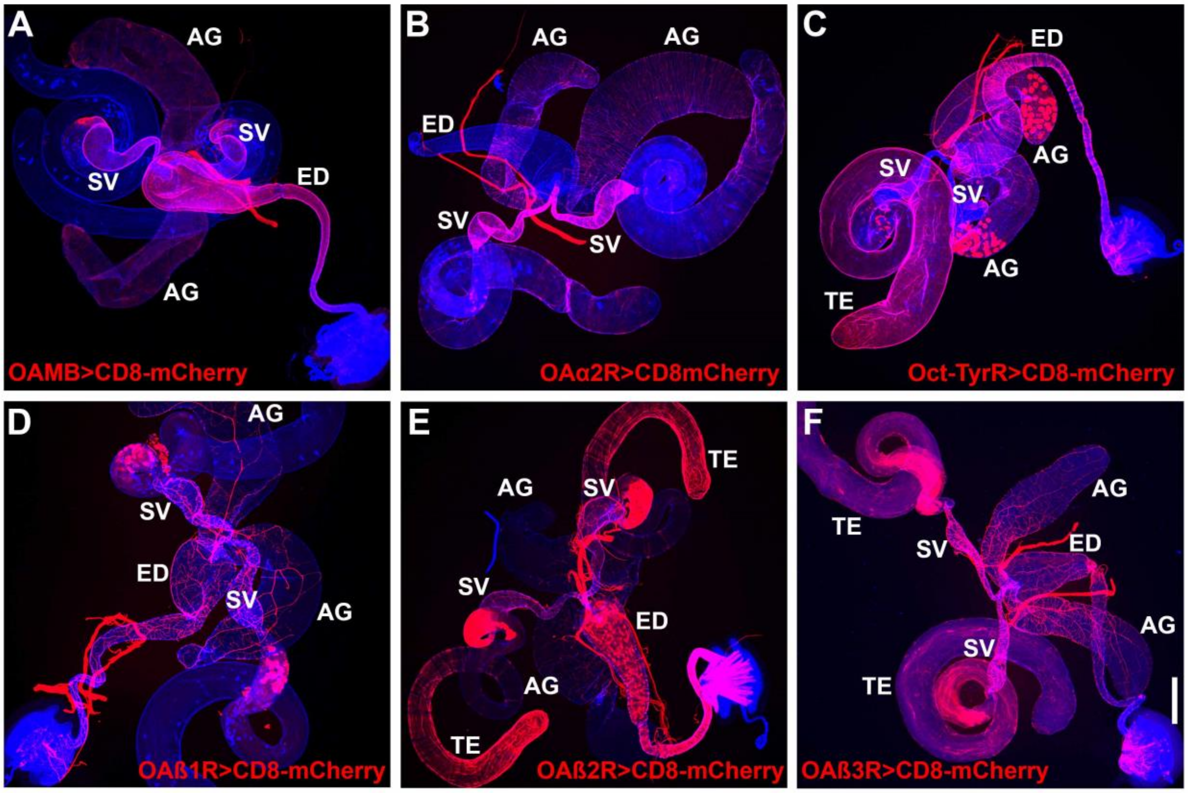

Figure 7S1. OA receptor GAL4 expression patterns in Drosophila male reproductive system. A) OAMB; B) OAα2R; C) Oct-TyrR; D) OAβ1R; E) OAβ2R; F) OAβ3R. Scale bar: 200μm.

bioRxiv preprint doi: https://doi.org/10.1101/2025.07.23.666348; this version posted July 28, 2025. The copyright holder for this preprint (which was not certified by peer review) is the author/funder, who has granted bioRxiv a license to display the preprint in perpetuity. It is made available under aCC-BY 4.0 International license.

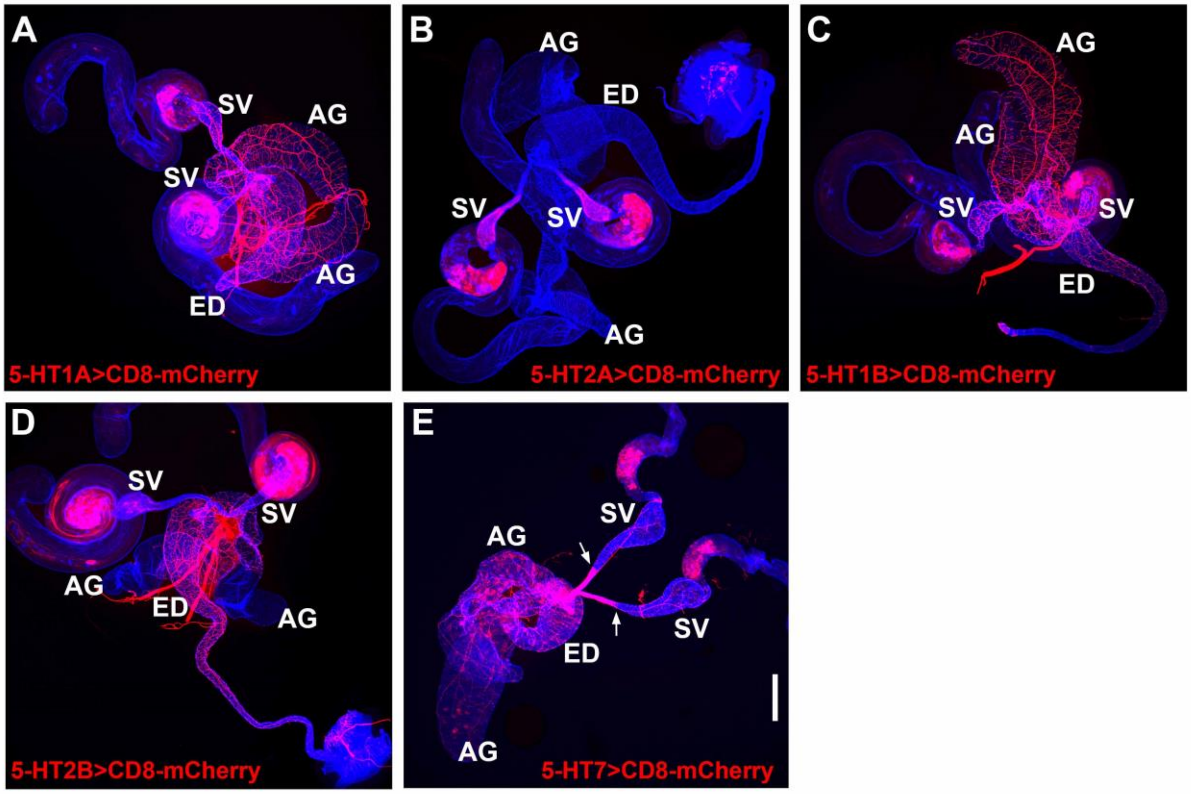

Figure 7S2. 5-HT receptor GAL4 expression patterns in Drosophila male reproductive system. A) 5-HT1A; B) 5-HT2A; C) 5-HT1B; D) 5-HT2B; E) 5-HT7. Scale bar: 200μm.

bioRxiv preprint doi: https://doi.org/10.1101/2025.07.23.666348; this version posted July 28, 2025. The copyright holder for this preprint (which was not certified by peer review) is the author/funder, who has granted bioRxiv a license to display the preprint in perpetuity. It is made available under aCC-BY 4.0 International license.

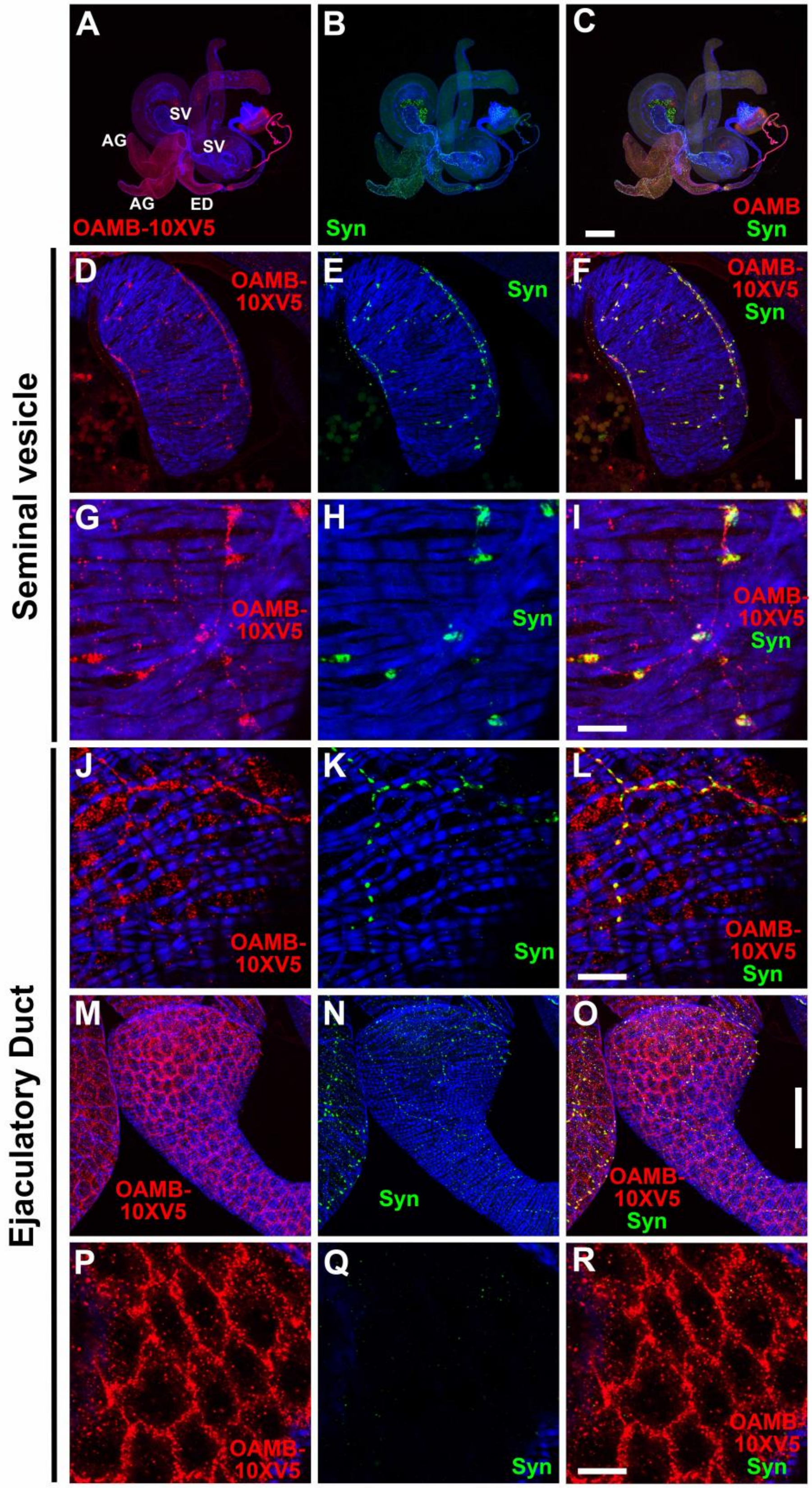

1398

bioRxiv preprint doi: https://doi.org/10.1101/2025.07.23.666348; this version posted July 28, 2025. The copyright holder for this preprint (which was not certified by peer review) is the author/funder, who has granted bioRxiv a license to display the preprint in perpetuity. It is made available under aCC-BY 4.0 International license.

Figure 8S1. OAMB expression in the Drosophila male reproductive system. A-C) Complete male reproductive system. A) OAMB-10XV5; B) Syn; C) OAMB-10XV5, Syn overlay. D-I) SV. D, G) OAMB-10XV5; E, H) Syn; F, I) OAMB-10XV5, Syn overlay. J-R) ED. J-L) muscle surface. J) OAMB-10XV5; K) Syn; L) OAMB-10XV5, Syn overlay. M-O) muscles and epithelial layers. M) OAMB-10XV5; N) Syn; O) OAMB-10XV5, Syn overlay. P-R) epithelial layer. P) OAMB-10XV5; Q) Syn; R) OAMB-10XV5, Syn overlay. Scale bars: C-200μm; F, O-50μm; I, L, R-10μm.

bioRxiv preprint doi: https://doi.org/10.1101/2025.07.23.666348; this version posted July 28, 2025. The copyright holder for this preprint (which was not certified by peer review) is the author/funder, who has granted bioRxiv a license to display the preprint in perpetuity. It is made available under aCC-BY 4.0 International license.

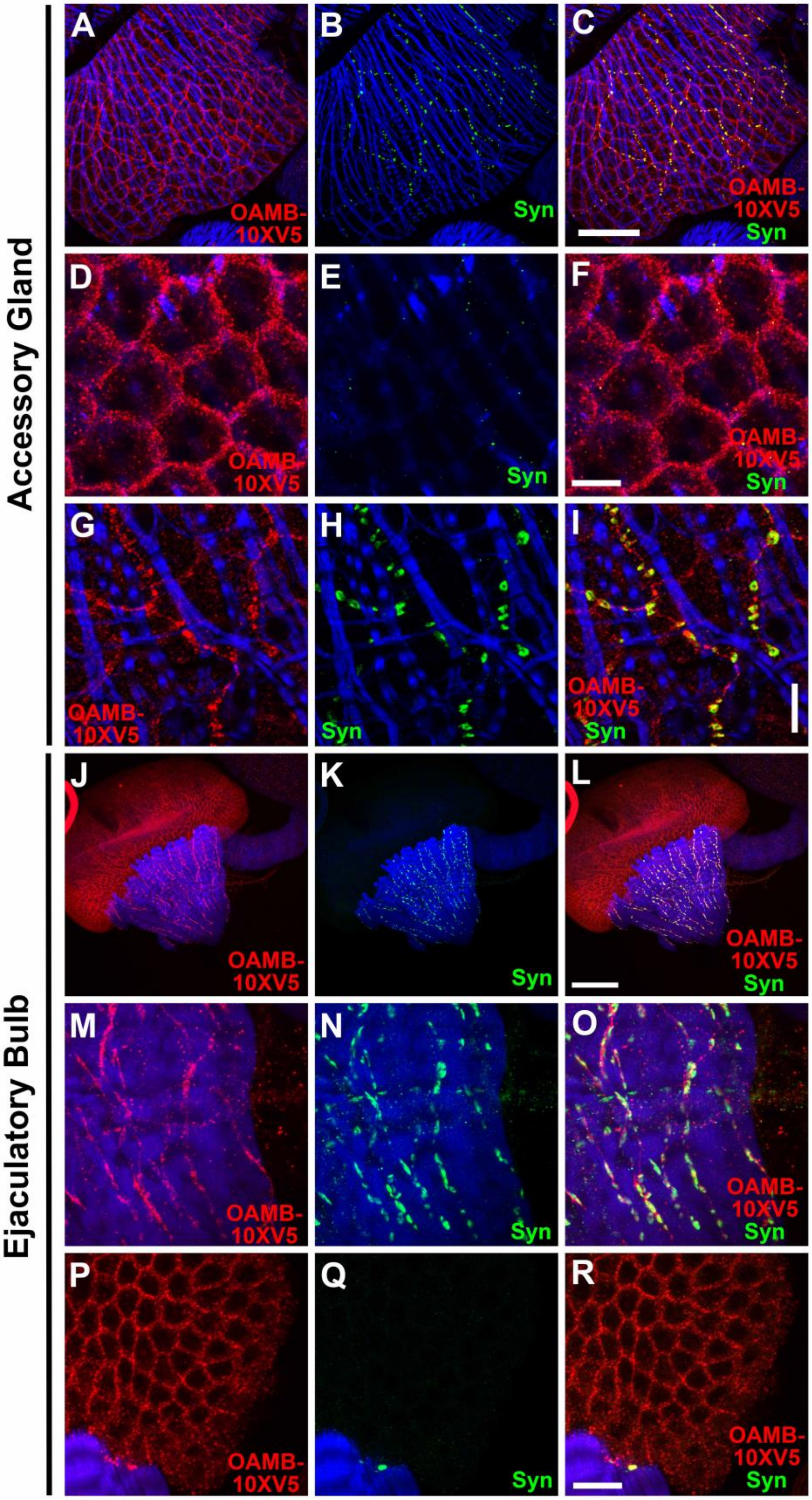

1406

bioRxiv preprint doi: https://doi.org/10.1101/2025.07.23.666348; this version posted July 28, 2025. The copyright holder for this preprint (which was not certified by peer review) is the author/funder, who has granted bioRxiv a license to display the preprint in perpetuity. It is made available under aCC-BY 4.0 International license.

Figure 8S2. OAMB expression in the Drosophila male reproductive system. A-C) AG. A) OAMB-10XV5; B) Syn; C) OAMB-10XV5, Syn overlay. D-F) Epithelial layer of AG. D) OAMB-10XV5; E) Syn; F) OAMB-10XV5, Syn overlay. G-I) Muscle layer of AG. G) OAMB-10XV5; H) Syn; I) OAMB-10XV5, Syn overlay. J-L) Ejaculatory bulb. J) OAMB-10XV5; K) Syn; L) OAMB-10XV5, Syn overlay. M-O) muscle layer of ejaculatory bulb. M) OAMB-10XV5; N) Syn; O) OAMB-10XV5, Syn overlay. P-Q) Epithelial layer of ejaculatory bulb. P) OAMB-10XV5; Q) Syn; R) OAMB-10XV5, Syn overlay. Scale bars: C, L-50μm; F, I, R-10μm.

bioRxiv preprint doi: https://doi.org/10.1101/2025.07.23.666348; this version posted July 28, 2025. The copyright holder for this preprint (which was not certified by peer review) is the author/funder, who has granted bioRxiv a license to display the preprint in perpetuity. It is made available under aCC-BY 4.0 International license.

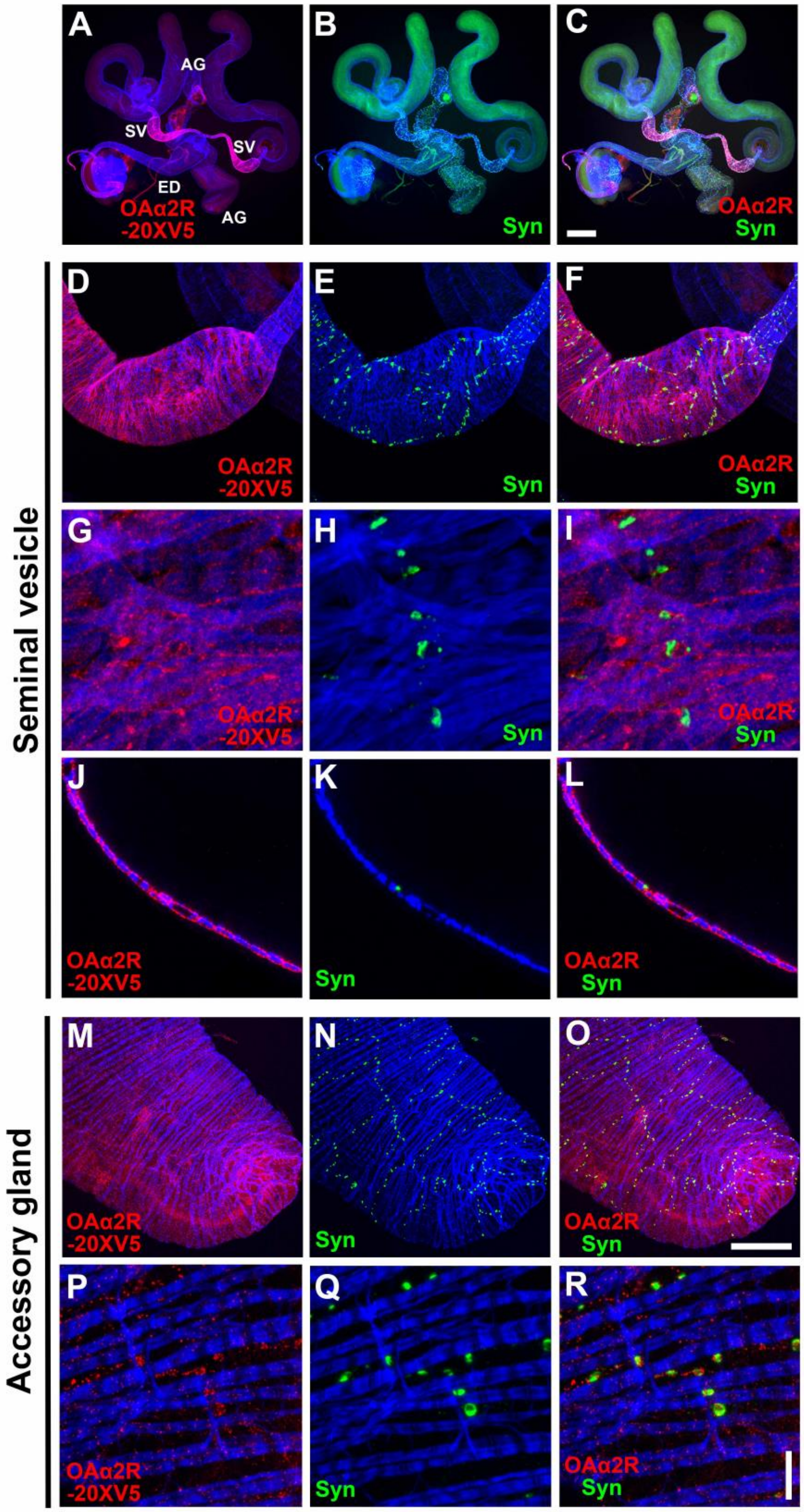

1415

bioRxiv preprint doi: https://doi.org/10.1101/2025.07.23.666348; this version posted July 28, 2025. The copyright holder for this preprint (which was not certified by peer review) is the author/funder, who has granted bioRxiv a license to display the preprint in perpetuity. It is made available under aCC-BY 4.0 International license.

Figure 8S3. OAα2R-20XV5 expression in the Drosophila male reproductive system. A-C) Complete male reproductive system. A) OAα2R-20XV5; B) Syn; C) OAα2R-20XV5, Syn overlay. D-I) SV. D, G) OAα2R-20XV5; E, H) Syn; F, I) OAα2R-20XV5, Syn overlay. Cross-section of SV. J) OAα2R-20XV5: K) Syn; L) OAα2R-20XV5, Syn overlay. M-R) AG. M, P) OAα2R-20XV5; N, Q) Syn; O, R) OAα2R-20XV5, Syn overlay. Scale bars: C-200μm; O-50μm; R-10μm.

bioRxiv preprint doi: https://doi.org/10.1101/2025.07.23.666348; this version posted July 28, 2025. The copyright holder for this preprint (which was not certified by peer review) is the author/funder, who has granted bioRxiv a license to display the preprint in perpetuity. It is made available under aCC-BY 4.0 International license.

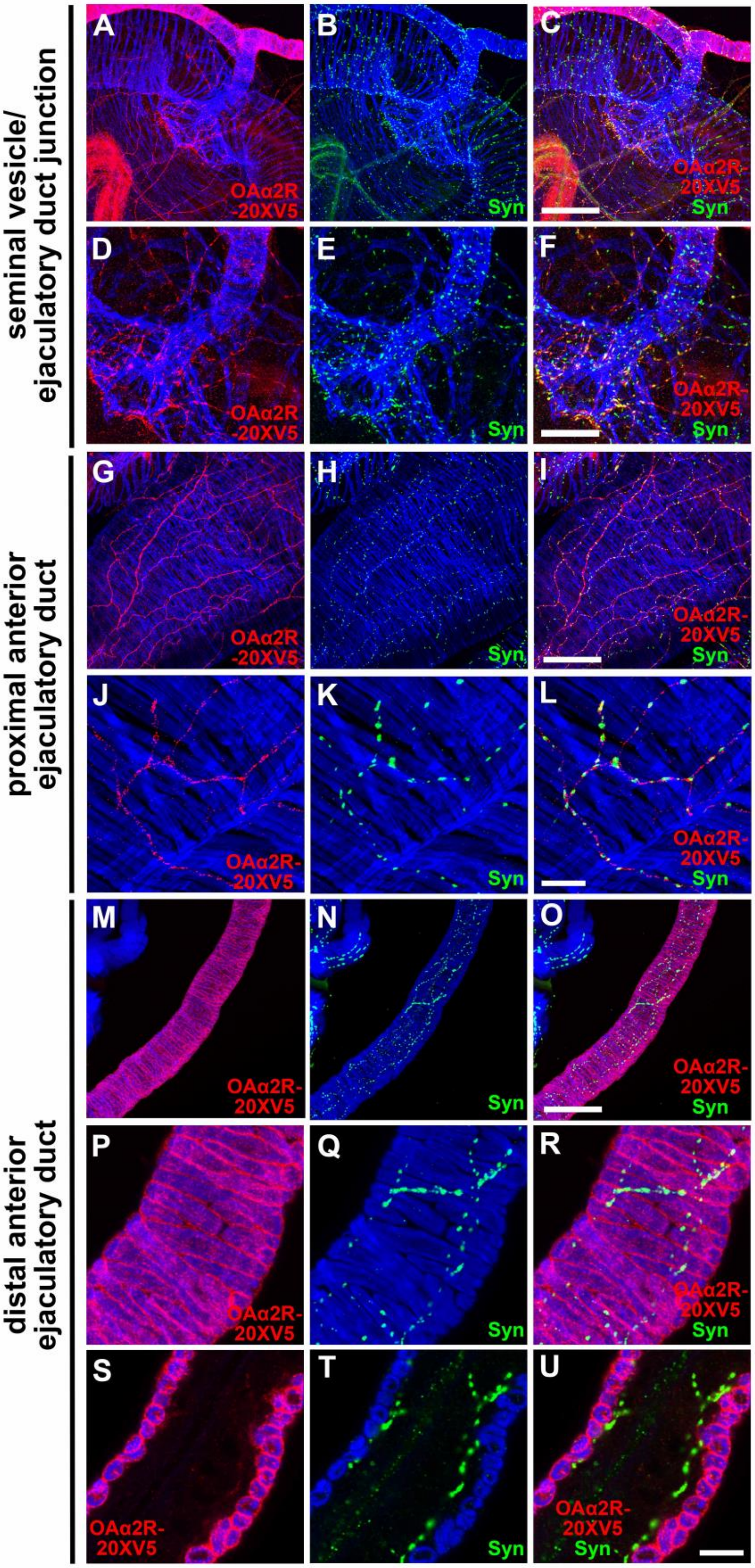

1422

bioRxiv preprint doi: https://doi.org/10.1101/2025.07.23.666348; this version posted July 28, 2025. The copyright holder for this preprint (which was not certified by peer review) is the author/funder, who has granted bioRxiv a license to display the preprint in perpetuity. It is made available under aCC-BY 4.0 International license.

Figure 8S4. OAα2R-20XV5 expression in the Drosophila male reproductive system. A-C) SV/ED junction. A, D) OAα2R-20XV5; B, E) Syn; C, F) OAα2R-20XV5, Syn overlay. G-L) Proximal anterior ED. G, J) OAα2R-20XV5; H, K) Syn; I, L) OAα2R-20XV5, Syn overlay. M-U) Distal anterior ED. M, P, S) OAα2R-20XV5; N, Q, T) Syn; O, R, U) OAα2R-20XV5, Syn overlay. Scale bars: C, I, O-50μm; F-25μm; L, U-10μm.

bioRxiv preprint doi: https://doi.org/10.1101/2025.07.23.666348; this version posted July 28, 2025. The copyright holder for this preprint (which was not certified by peer review) is the author/funder, who has granted bioRxiv a license to display the preprint in perpetuity. It is made available under aCC-BY 4.0 International license.

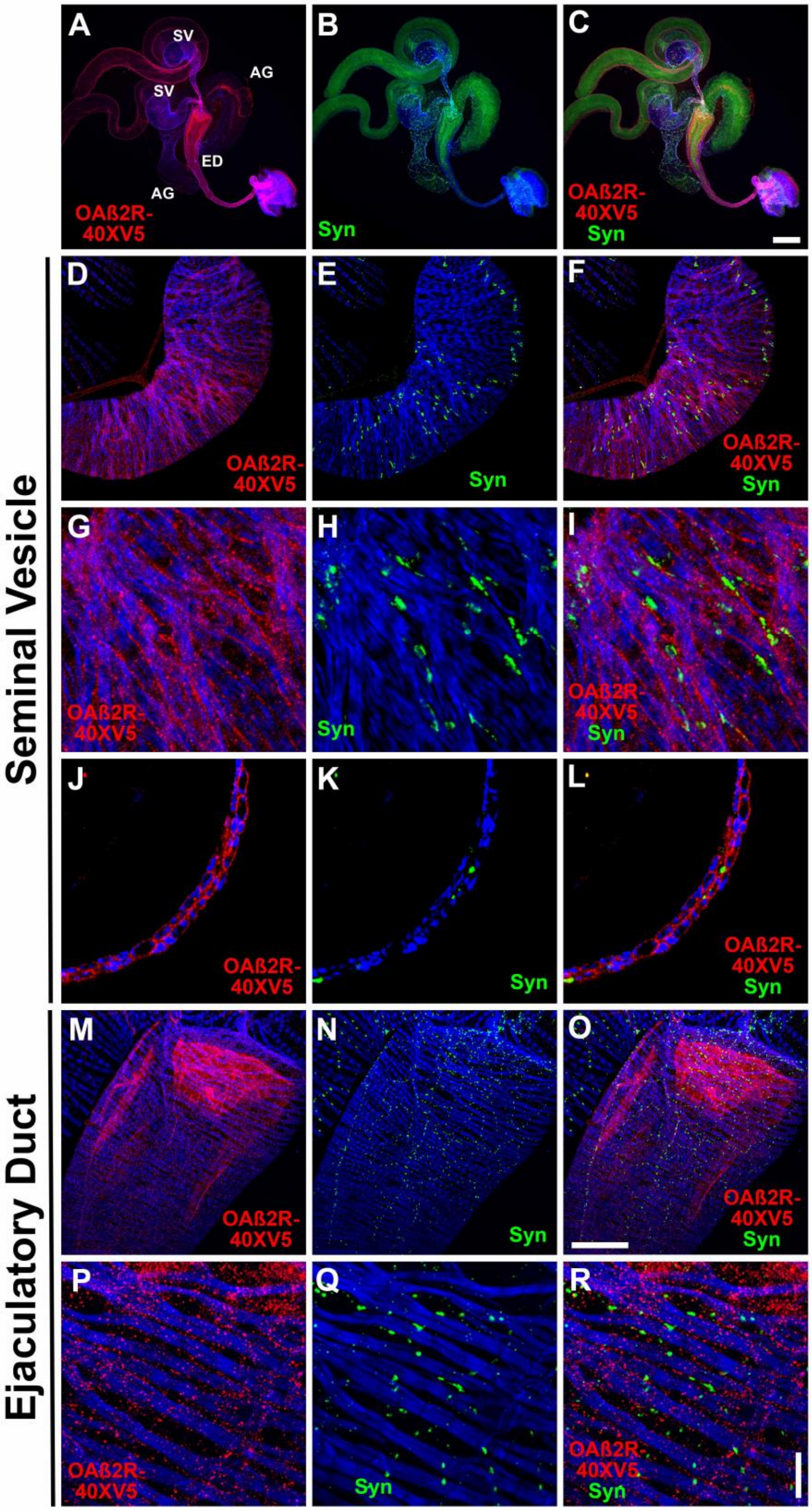

1428

bioRxiv preprint doi: https://doi.org/10.1101/2025.07.23.666348; this version posted July 28, 2025. The copyright holder for this preprint (which was not certified by peer review) is the author/funder, who has granted bioRxiv a license to display the preprint in perpetuity. It is made available under aCC-BY 4.0 International license.

Figure 8S5. OAβ2R-40XV5 expression in the Drosophila male reproductive system. A-C) Complete male reproductive system. A) OAβ2R-40XV5; B) Syn; C) OAβ2R-40XV5, Syn overlay. D-I) SV. D, G,) OAβ2R; E, H) Syn; F, I) OAβ2R-40XV5, Syn overlay. J-L) Cross section of SV. J) OAβ2R-40XV5; K) Syn; L) OAβ2R-40XV5, Syn overlay. M-R) ED. M, P) OAβ2R; N, Q) Syn; O, R) OAβ2R-40XV5, Syn overlay. Scale bars: C-200μm; O-50μm; R-10μm.

bioRxiv preprint doi: https://doi.org/10.1101/2025.07.23.666348; this version posted July 28, 2025. The copyright holder for this preprint (which was not certified by peer review) is the author/funder, who has granted bioRxiv a license to display the preprint in perpetuity. It is made available under aCC-BY 4.0 International license.

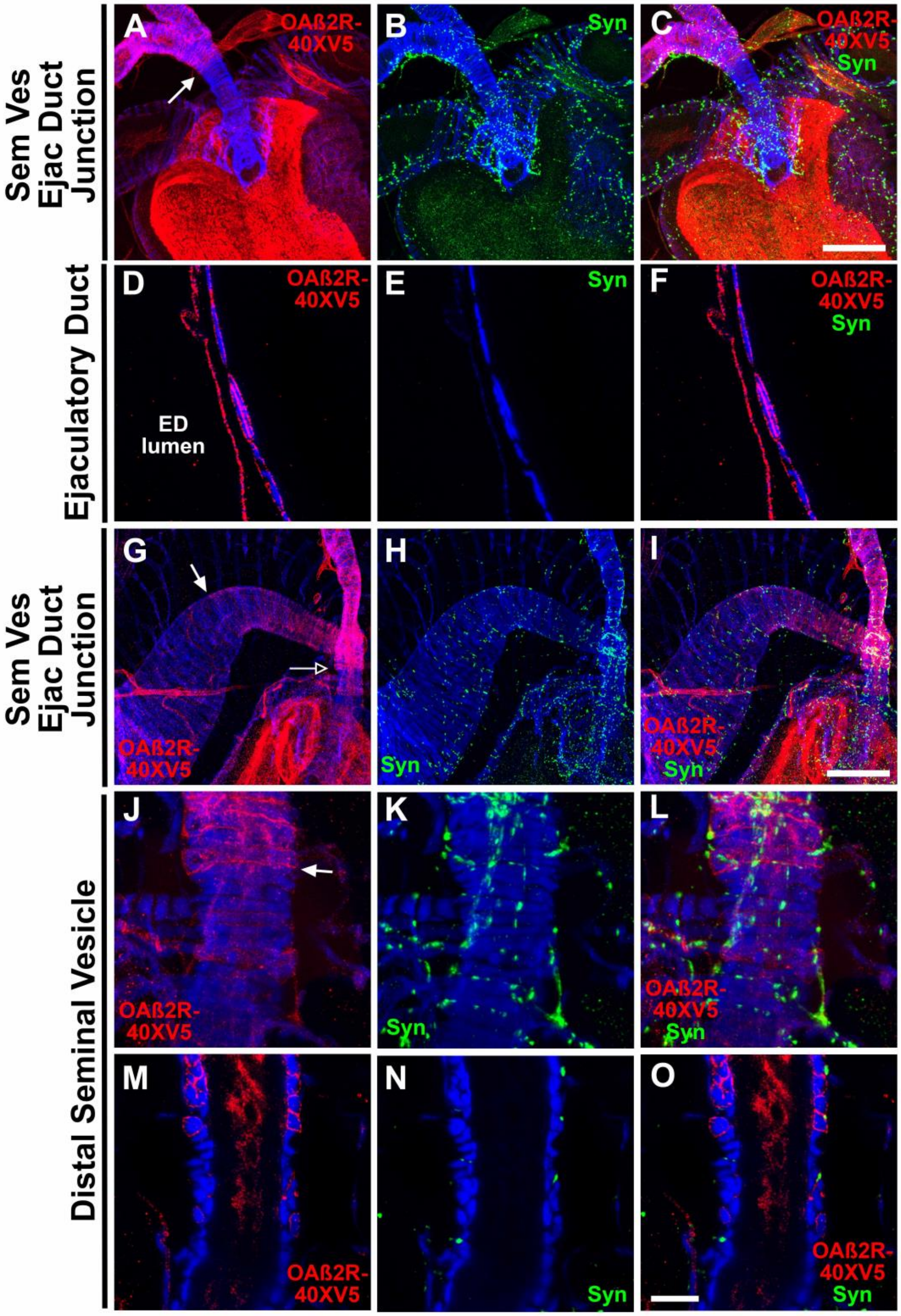

bioRxiv preprint doi: https://doi.org/10.1101/2025.07.23.666348; this version posted July 28, 2025. The copyright holder for this preprint (which was not certified by peer review) is the author/funder, who has granted bioRxiv a license to display the preprint in perpetuity. It is made available under aCC-BY 4.0 International license.

Figure 8S6. OAβ2R-40XV5 expression in the Drosophila male reproductive system. A-C) Junction of SV and ED. A) OAβ2R-40XV5; B) Syn; C) OAβ2R-40XV5, Syn overlay. D-F) Cross section of ED. D) OAβ2R-40XV5; E) Syn; F) OAβ2R-40XV5, Syn overlay. G-I) Junction of SV and ED. G) OAβ2R-40XV5; H) Syn; I) OAβ2R-40XV5, Syn overlay. J-L) Distal SV. J) OAβ2R-40XV5; E) Syn; F) OAβ2R-40XV5, Syn overlay. M-O) Cross section of distal SV. M) OAβ2R-40XV5; N) Syn; O) OAβ2R-40XV5, Syn overlay. Scale bars: C, I-50μm; O-10μm.

bioRxiv preprint doi: https://doi.org/10.1101/2025.07.23.666348; this version posted July 28, 2025. The copyright holder for this preprint (which was not certified by peer review) is the author/funder, who has granted bioRxiv a license to display the preprint in perpetuity. It is made available under aCC-BY 4.0 International license.

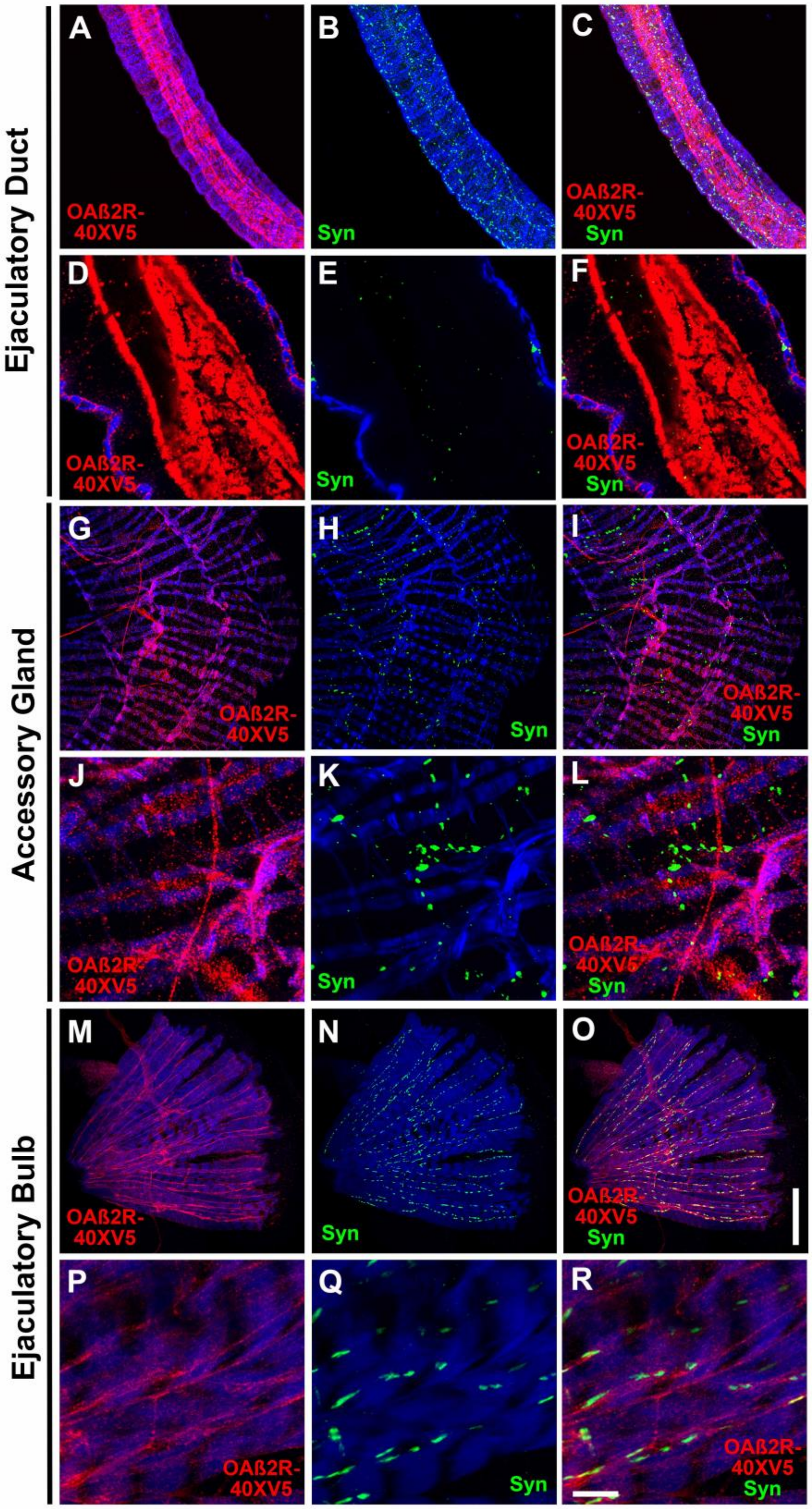

1443

bioRxiv preprint doi: https://doi.org/10.1101/2025.07.23.666348; this version posted July 28, 2025. The copyright holder for this preprint (which was not certified by peer review) is the author/funder, who has granted bioRxiv a license to display the preprint in perpetuity. It is made available under aCC-BY 4.0 International license.

Figure 8S7. OAβ2R-40XV5 expression in the Drosophila male reproductive system. A-C) Distal anterior ED. A) OAβ2R-40XV5; B) Syn; C) OAβ2R-40XV5, Syn overlay. D-F) Cross section of distal anterior ED. D) OAβ2R-40XV5; E) Syn; F) OAβ2R-40XV5, Syn overlay. G-L) AG. G, J) OAβ2R-40XV5; H, K) Syn; I, L) OAβ2R-40XV5, Syn overlay. M-O) Ejaculatory bulb. M, O) OAβ2R-40XV5; N, Q) Syn; O, R) OAβ2R-40XV5, Syn overlay. Scale bars: O-50μm; R-10μm.

bioRxiv preprint doi: https://doi.org/10.1101/2025.07.23.666348; this version posted July 28, 2025. The copyright holder for this preprint (which was not certified by peer review) is the author/funder, who has granted bioRxiv a license to display the preprint in perpetuity. It is made available under aCC-BY 4.0 International license.

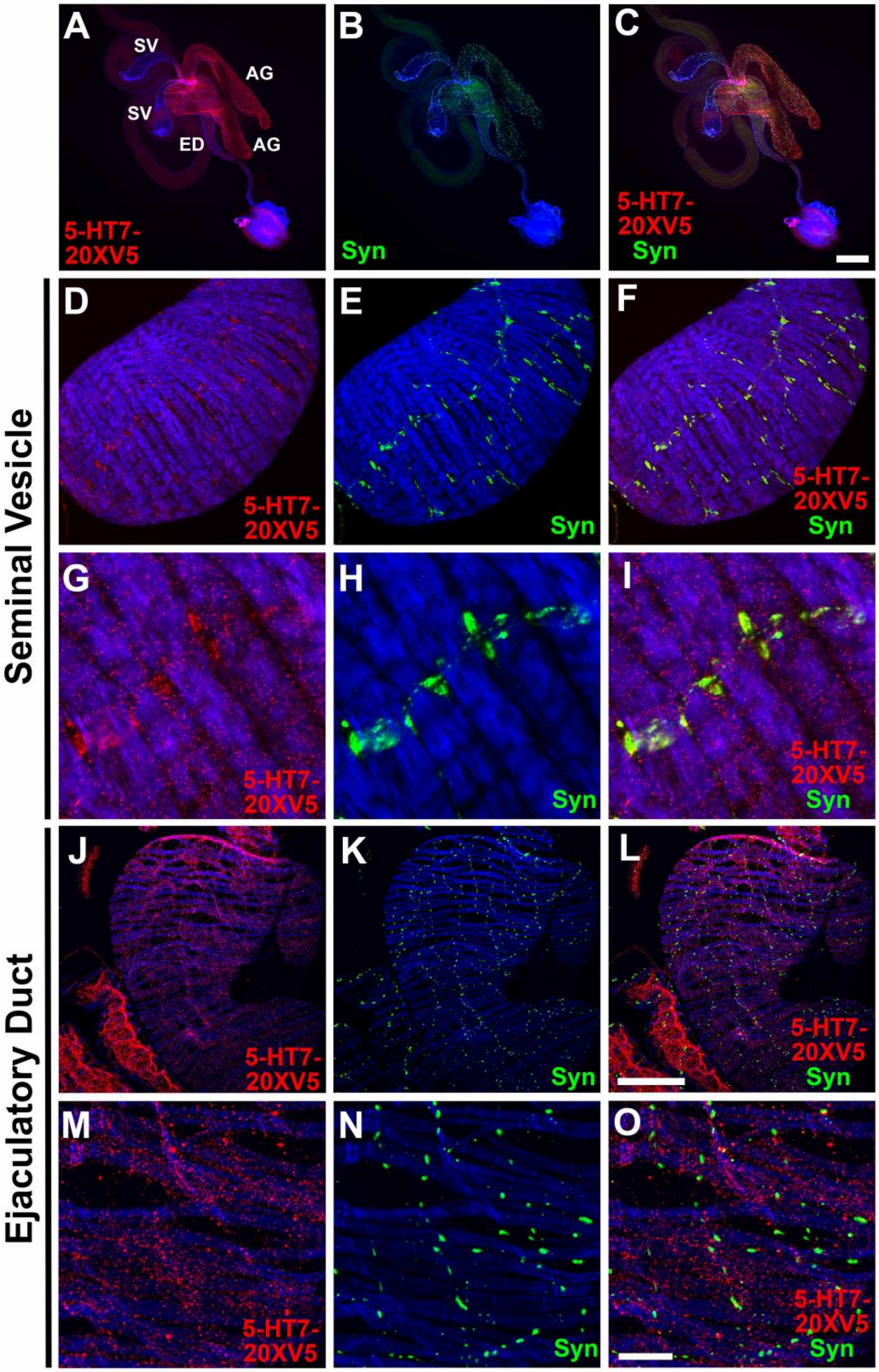

1450

bioRxiv preprint doi: https://doi.org/10.1101/2025.07.23.666348; this version posted July 28, 2025. The copyright holder for this preprint (which was not certified by peer review) is the author/funder, who has granted bioRxiv a license to display the preprint in perpetuity. It is made available under aCC-BY 4.0 International license.

Figure 8S8. 5-HT7-20XV5 expression in the Drosophila male reproductive system. A-C) Complete male reproductive system. A) 5-HT7-20XV5.; B) Syn; C) 5-HT7-20XV5, Syn overlay. D-I) SV. D, G) 5-HT7-20XV5.; E, H) Syn; F, I) 5-HT7-20XV5, Syn overlay. J-O) ED. J, M) 5-HT7-20XV5.; K, N) Syn; L, O) 5-HT7-20XV5, Syn overlay. Scale bars: C-200μm; L-50μm; O-10μm.

bioRxiv preprint doi: https://doi.org/10.1101/2025.07.23.666348; this version posted July 28, 2025. The copyright holder for this preprint (which was not certified by peer review) is the author/funder, who has granted bioRxiv a license to display the preprint in perpetuity. It is made available under aCC-BY 4.0 International license.

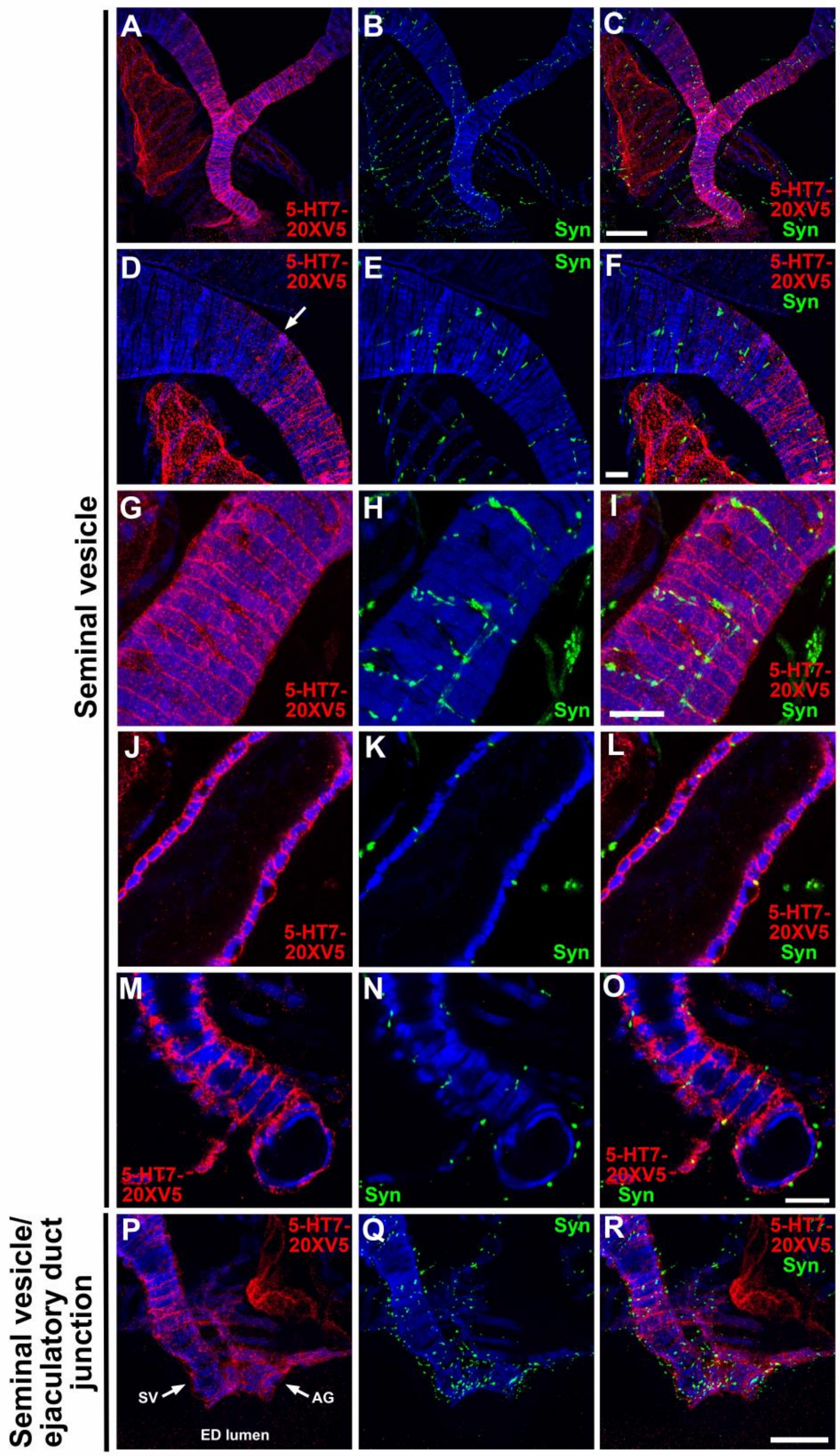

1456

bioRxiv preprint doi: https://doi.org/10.1101/2025.07.23.666348; this version posted July 28, 2025. The copyright holder for this preprint (which was not certified by peer review) is the author/funder, who has granted bioRxiv a license to display the preprint in perpetuity. It is made available under aCC-BY 4.0 International license.

Figure 8S9. 5-HT7-20XV5 expression in the Drosophila male reproductive system. A-I) SV. A, D, G,) 5-HT7-20XV5.; B, E, H,) Syn; C, F, I,) 5-HT7-20XV5, Syn overlay. J-L) Cross section of SV. J) 5-HT7-20XV5.; K) Syn; L) 5-HT7-20XV5, Syn overlay. M-O) SV terminus. M) 5-HT7-20XV5.; N) Syn; O) 5-HT7-20XV5, Syn overlay. P-R) SV/ED junction. P) 5-HT7-20XV5.; Q) Syn; R) 5-HT7-20XV5, Syn overlay. Scale bars: C, G-50μm; F, O-10μm; R-25μm.

bioRxiv preprint doi: https://doi.org/10.1101/2025.07.23.666348; this version posted July 28, 2025. The copyright holder for this preprint (which was not certified by peer review) is the author/funder, who has granted bioRxiv a license to display the preprint in perpetuity. It is made available under aCC-BY 4.0 International license.

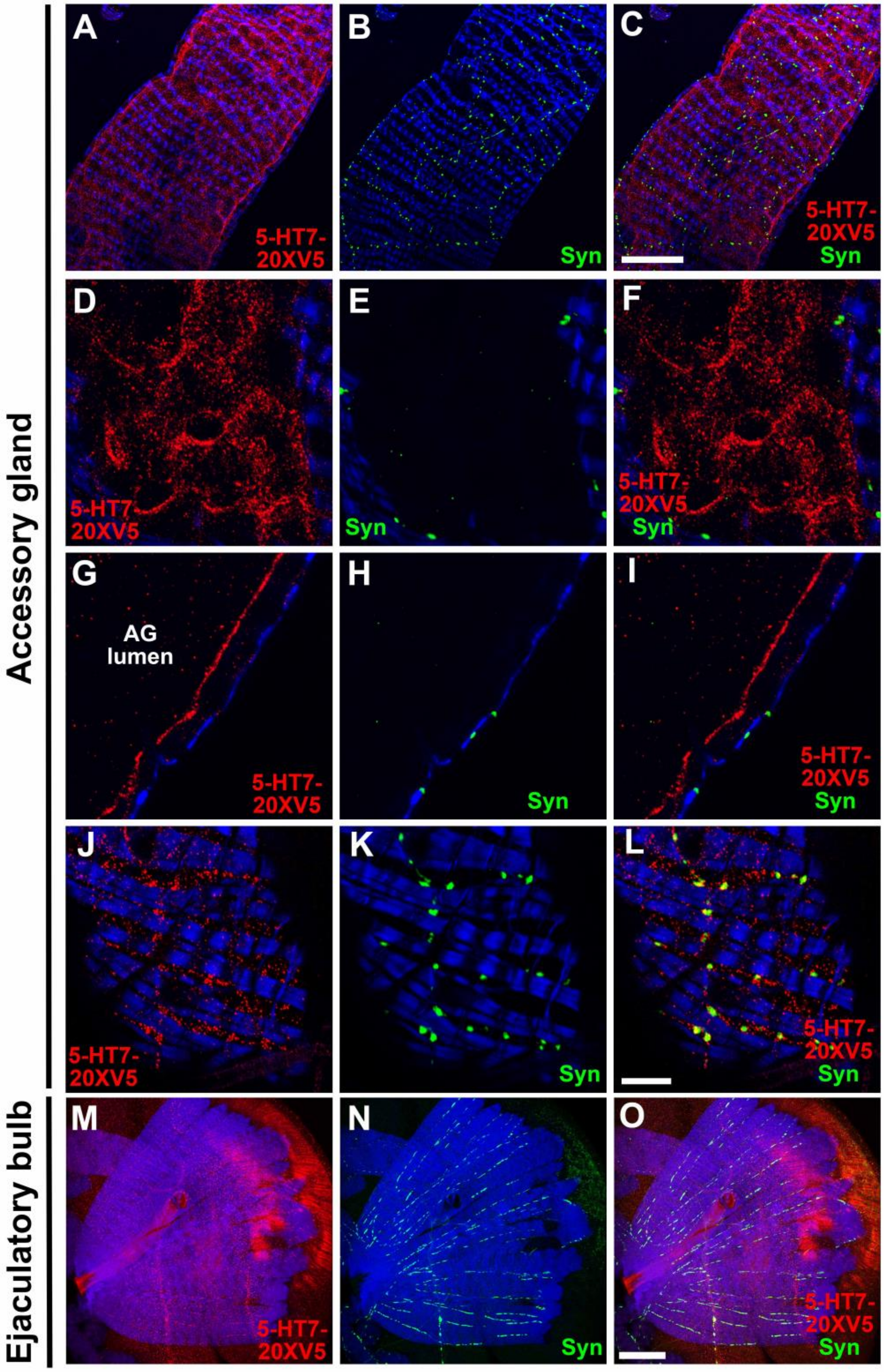

1463

bioRxiv preprint doi: https://doi.org/10.1101/2025.07.23.666348; this version posted July 28, 2025. The copyright holder for this preprint (which was not certified by peer review) is the author/funder, who has granted bioRxiv a license to display the preprint in perpetuity. It is made available under aCC-BY 4.0 International license.

Figure 8S10. 5-HT7-20XV5 expression in the Drosophila male reproductive system. A-C) AG. A) 5-HT7-20XV5.; B) Syn; C) 5-HT7-20XV5, Syn overlay. D-F) Epithelial layer of AG. D) 5-HT7-20XV5.; E) Syn; F) 5-HT7-20XV5, Syn overlay. G-I) Cross-section of epithelial layer of AG. G) 5-HT7-20XV5.; H) Syn; I) 5-HT7-20XV5, Syn overlay. J-L) Muscle layer of AG. J) 5-HT7-20XV5.; K) Syn; L) 5-HT7-20XV5, Syn overlay. M-O) Ejaculatory bulb. M) 5-HT7-20XV5.; N) Syn; O) 5-HT7-20XV5, Syn overlay. Scale bars: C, O-50μm; L-10μm.

bioRxiv preprint doi: https://doi.org/10.1101/2025.07.23.666348; this version posted July 28, 2025. The copyright holder for this preprint (which was not certified by peer review) is the author/funder, who has granted bioRxiv a license to display the preprint in perpetuity. It is made available under aCC-BY 4.0 International license.

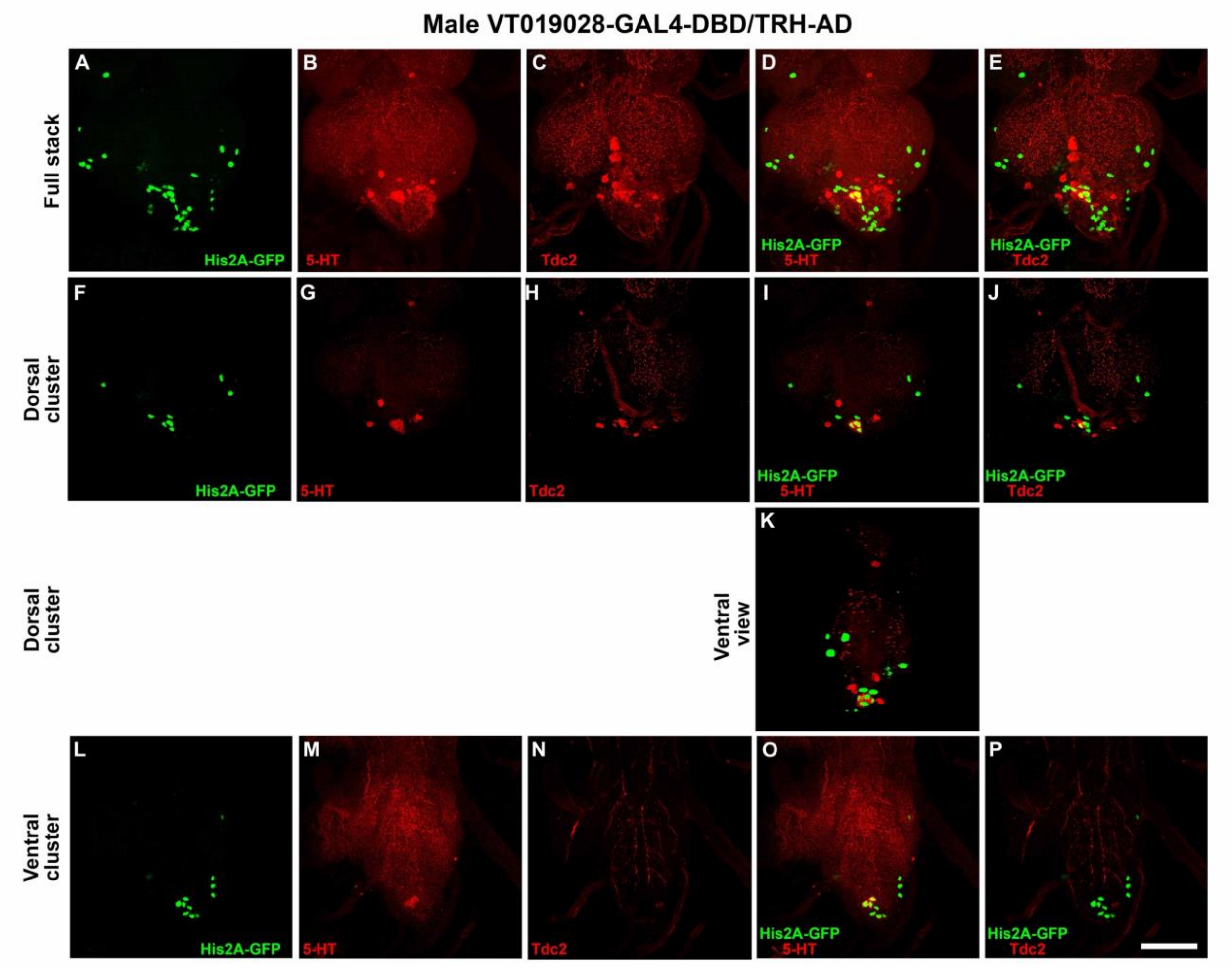

Figure 9S1. High resolution images of nuclear expression of *VT019028-GAL4-DBD* in combination with *TRH-AD* in posterior ventral nerve cord of Drosophila male adult nervous system relative to 5-HT and Tdc2. A-E) Dorsal view of complete stack of confocal images. A) His2A-GFP; B) 5-HT; C) Tdc2; D) His2A-GFP, 5-HT overlay; E) His2A-GFP, Tdc2 overlay. F) Ventral view of His2A-GFP/5-HT overlay of subset of slices containing the dorsal cluster of 5-HT neurons; G-K) Dorsal view of subset of slices containing the dorsal cluster of 5-HT neurons. G) His2A-GFP; H) 5-HT; I) Tdc2; J) His2A-GFP, 5-HT overlay; K) His2A-GFP, Tdc2 overlay. L-P) Dorsal view of subset of slices containing the ventral cluster of 5-HT neurons. L) His2A-GFP; M) 5-HT; N) Tdc2; O) His2A-GFP, 5-HT overlay; P) His2A-GFP, Tdc2 overlay. Scale bar: 50μm.

bioRxiv preprint doi: https://doi.org/10.1101/2025.07.23.666348; this version posted July 28, 2025. The copyright holder for this preprint (which was not certified by peer review) is the author/funder, who has granted bioRxiv a license to display the preprint in perpetuity. It is made available under aCC-BY 4.0 International license.

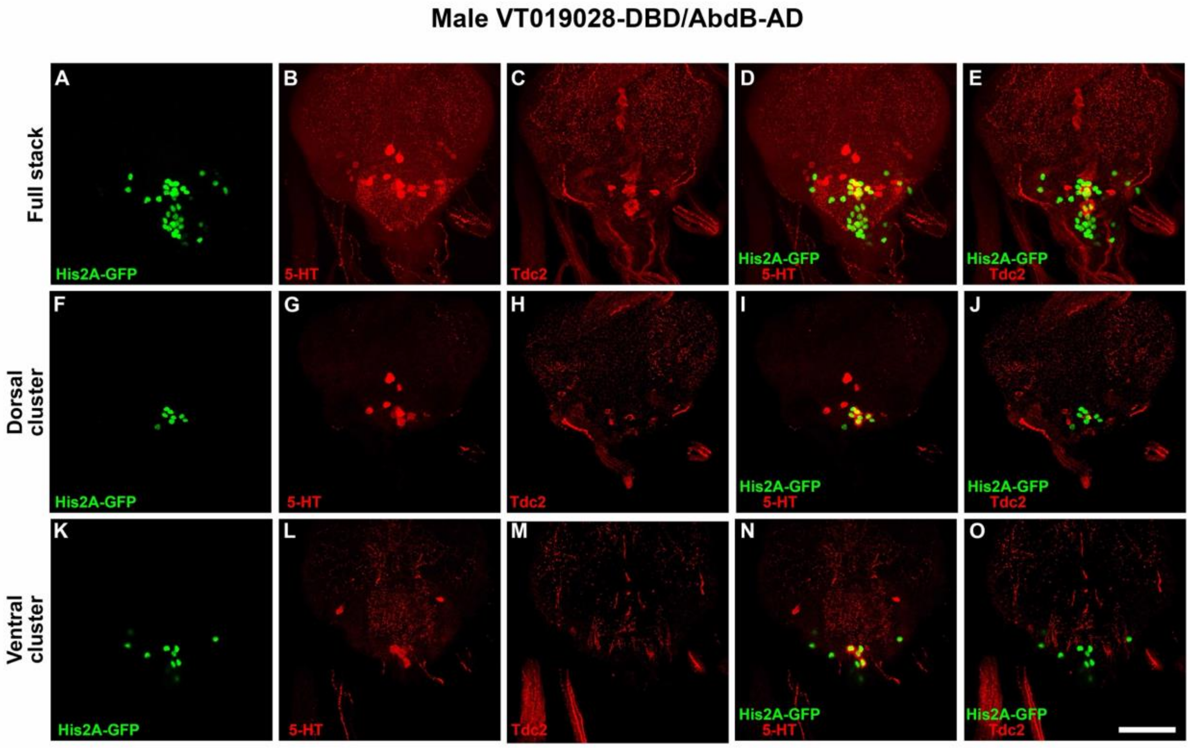

Figure 9S2. High resolution images of nuclear expression of *VT019028-GAL4-DBD* in combination with *AbdB-AD* in posterior ventral nerve cord of Drosophila male adult nervous system relative to 5-HT and Tdc2. A-E) Dorsal view of complete stack of confocal images. A) His2A-GFP; B) 5-HT; C) Tdc2; D) His2A-GFP, 5-HT overlay; E) His2A-GFP, Tdc2 overlay. F-J) Dorsal view of subset of slices containing the dorsal cluster of 5-HT neurons. F) His2A-GFP; G) 5-HT; H) Tdc2; I) His2A-GFP, 5-HT overlay; J) His2A-GFP, Tdc2 overlay. K-O) Dorsal view of subset of slices containing the ventral cluster of 5-HT neurons. K) His2A-GFP; L) 5-HT; M) Tdc2; N) His2A-GFP, 5-HT overlay; O) His2A-GFP, Tdc2 overlay. Scale bar: 50μm.

bioRxiv preprint doi: https://doi.org/10.1101/2025.07.23.666348; this version posted July 28, 2025. The copyright holder for this preprint (which was not certified by peer review) is the author/funder, who has granted bioRxiv a license to display the preprint in perpetuity. It is made available under aCC-BY 4.0 International license.

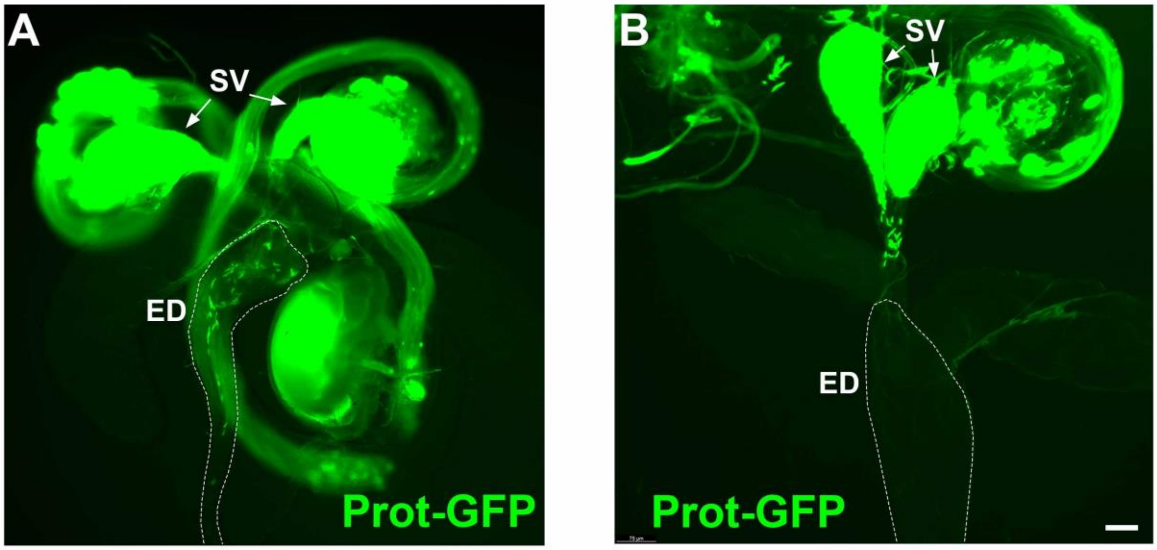

Figure 10S1. Expression of ProtB-GFP fluorescent marker of sperm nuclei in male reproductive system of control and *TRH-AD/VT019028-GAL4-DBD* neuron-silenced flies interrupted mid-mating. A) control. B) *TRH-AD/VT019028-GAL4-DBD* neurons silenced with BONT-C. Prot-GFP-Protamine GFP. SV-SV, ED-ED. Scale bar: 75µm.

bioRxiv preprint doi: https://doi.org/10.1101/2025.07.23.666348; this version posted July 28, 2025. The copyright holder for this preprint (which was not certified by peer review) is the author/funder, who has granted bioRxiv a license to display the preprint in perpetuity. It is made available under aCC-BY 4.0 International license.

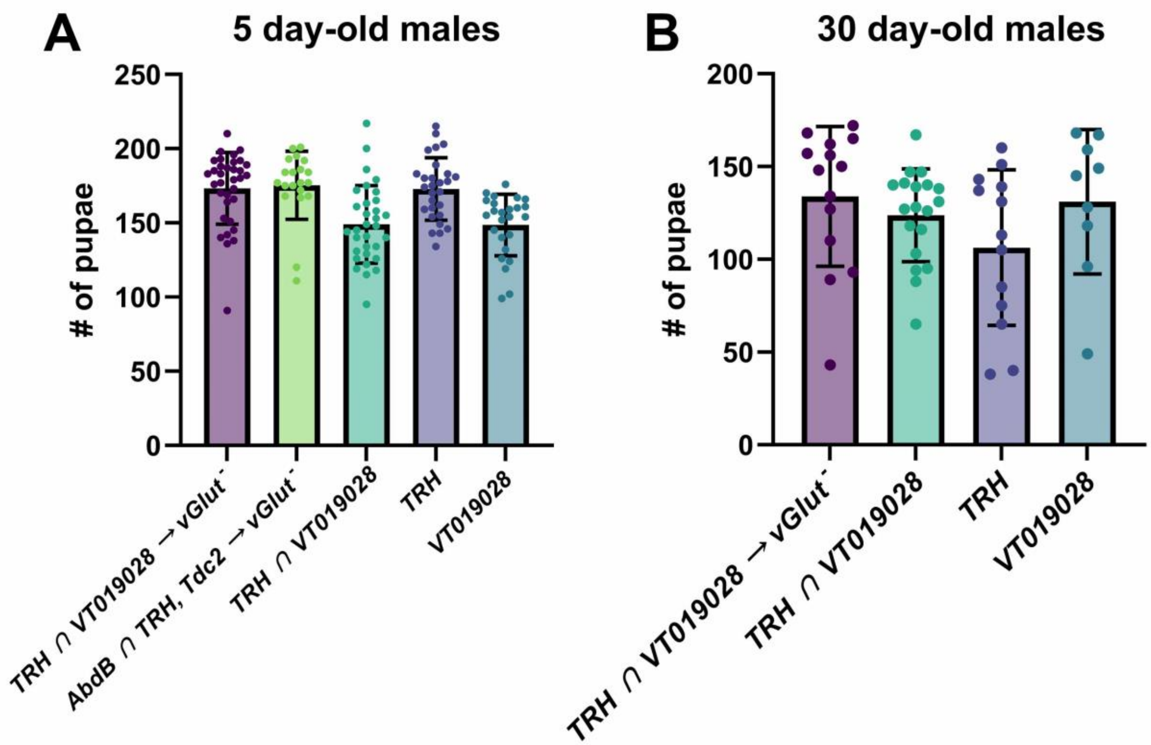

Figure 10S2. Comparison of male drosophila fecundity between genotypes. Bar plots show the mean ± SD for each group with individual data points overlaid on each bar. Figure 10S1A males were aged 5 days before copulation. Figure 10S1B males were aged 30 days before copulation. (A) Columns from left to right: TRH ∩ VT019028 → vGlut-: n=34, mean=173.2, SD=24.22. AbdB ∩ TRH, Tdc2 → vGlut-: n=20, mean=175.3, SD=22.92. TRH ∩ VT019028: n=32, mean=149.0, SD=26.10. TRH: n=28, mean=172.8, SD=21.11. VT019028: n=25, mean=148.6, SD=20.81. (B) TRH ∩ VT019028 → vGlut-: n=14, mean=133.9, SD=37.64. TRH ∩ VT019028: n=19, mean=123.7, SD, 25.02. TRH: n=13, mean=106.3, SD=41.93. VT019028: n=9, mean=131.0, SD=38.88.

bioRxiv preprint doi: https://doi.org/10.1101/2025.07.23.666348; this version posted July 28, 2025. The copyright holder for this preprint (which was not certified by peer review) is the author/funder, who has granted bioRxiv a license to display the preprint in perpetuity. It is made available under aCC-BY 4.0 International license.

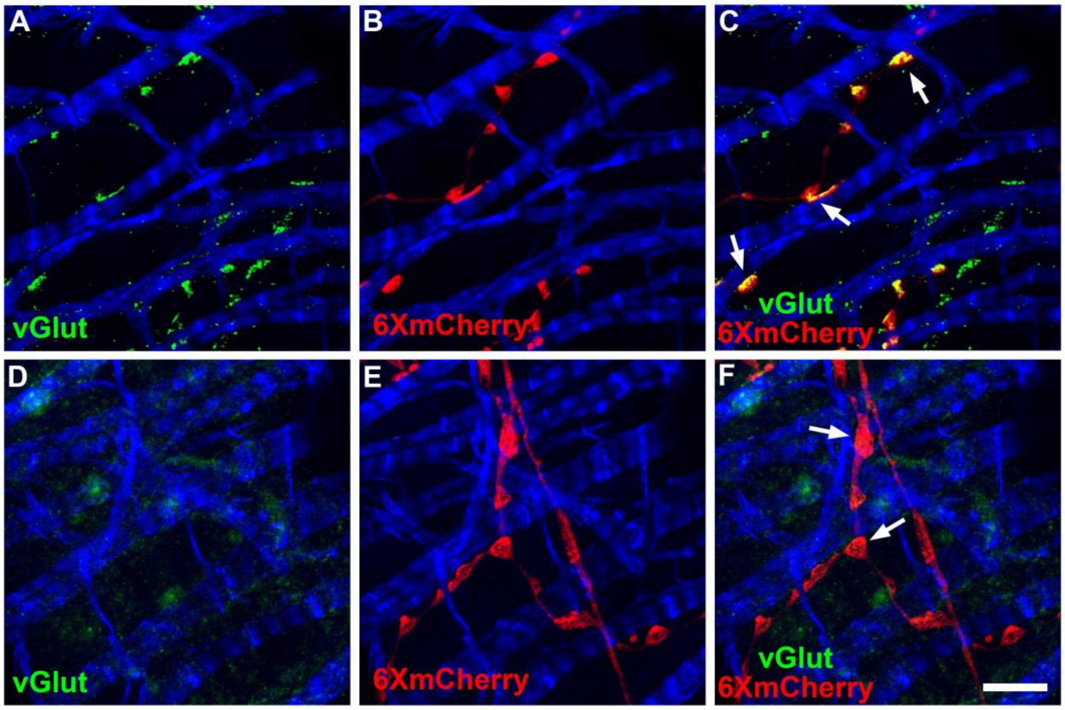

Figure 10S3. vGlut expression in AGs of control and vGlut conditional mutant. A-C) control male. A) vGlut; B) 6XmCherry; C) overlay. D-F) experimental male conditionally silenced for vGlut in *TRH-AD*/*VT019028-GAL4* neurons. Scale bar: 50μm.

Video 1. Male reproductive system of a ProtB-GFP control. Sperm with green fluorescent nuclei are visible in the ejaculatory duct of a male subjected to a mid-mating interruption.

Video 2. Male reproductive system of a ProtB-GFP experimental male in which a subset of the *TRH-AD*/*VT019028-GAL4-DBD* subset of serotonergic neurons innervating the male reproductive system have been silenced. Sperm with green fluorescent nuclei are restricted to the seminal vesicle and never enter the ejaculatory duct from a male subjected to a mid-mating interruption.

Video 3. GCAMP8m Ca++ imaging of an ejaculatory duct experiencing spontaneous peristaltic waves of muscle contractions.

Video 4. GCAMP8m Ca++ imaging of the SV/ED junction showing the initiation site of the spontaneous peristaltic waves.

Video 5. GCAMP8m Ca++ imaging of the SV/ED junction showing the subtle variation in the initiation site of spontaneous peristaltic waves as compared to Video 4.

bioRxiv preprint doi: https://doi.org/10.1101/2025.07.23.666348; this version posted July 28, 2025. The copyright holder for this preprint (which was not certified by peer review) is the author/funder, who has granted bioRxiv a license to display the preprint in perpetuity. It is made available under aCC-BY 4.0 International license.

Video 6. GCAMP8m Ca++ imaging of the SV showing spontaneous uncoordinated muscle activity.

Video 7. GCAMP8m Ca++ imaging of an AG showing spontaneous uncoordinated muscle activity.

Video 8. GCAMP8m Ca++ imaging of a testes showing spontaneous uncoordinated muscle activity.

Video 9. GCAMP8m Ca++ imaging of the AG showing strong coordinated muscle activity.

Complete Genotypes

Figure 3S1. *yw*; *B3RT-vGlut-LexA*/*20XUAS-6XGFP*; TRH-GAL4/*13XLexAop-6XmCherry*.

bioRxiv preprint doi: https://doi.org/10.1101/2025.07.23.666348; this version posted July 28, 2025. The copyright holder for this preprint (which was not certified by peer review) is the author/funder, who has granted bioRxiv a license to display the preprint in perpetuity. It is made available under aCC-BY 4.0 International license.

Figure 3S2. *yw*; *B3RT-vGlut-LexA*/*Tdc2-GAL4*; TRH-GAL4/*20XUAS-6XGFP*/*13XLexAop-6XmCherry*.

Figure 3S3. A-C) *yw*; *B3RT-vGlut-LexA*/*20XUAS-6XGFP*; fru-GAL4/*13XLexAop-6XmCherry*. D) *yw*; *Tdc2-AD*/*dsx-GAL4-DBD*, *UAS-CD8-mCherry*. E) *yw*; *TRH-AD*/+; *dsx-GAL4-DBD*, *UAS-CD8-mCherry*/+.

Figure 4S1. A-L) *w*, *TBH-GFP*; *vGlut-40XV5*/*vGlut-40XV5*. M-X) yw; *vGlut-40XV5*/*vGlut-40XV5*.

Figure 4S2. A-L) *w*, *TBH-GFP*; *vGlut-40XV5*/*vGlut-40XV5*. M-X) yw; *vGlut-40XV5*/*vGlut-40XV5*.

Figure 4S3. A-L) *w*, *TBH-GFP*; *vGlut-40XV5*/*vGlut-40XV5*. M-X) yw; *vGlut-40XV5*/*vGlut-40XV5*.

Figure 4S4. A-F) *w*, *TBH-GFP*; *vGlut-40XV5*/*vGlut-40XV5*.

Figure 5S1. A-L) *yw*; *B2RT-STOP-B2RT-40XMYC*, *RSRT-STOP-RSRT-6XV5-vMAT*/+; *TRH-GAL4*, *UAS-CD8-mCherry*, *UAS-B2*, *UAS-R*. M-O) *yw*; *B2RT-STOP-B2RT-40XMYC*, *RSRT-STOP-RSRT-6XV5-vMAT*/*Tdc2-GAL4*; *UAS-CD8-mCherry*, *UAS-B2*, *UAS-R*/+.

Figure 5S2. A-L) *yw*; *B2RT-STOP-B2RT-40XMYC*, *RSRT-STOP-RSRT-6XV5-vMAT*/+; *TRH-GAL4*, *UAS-CD8-mCherry*, *UAS-B2*, *UAS-R*. M-O) *yw*; *B2RT-STOP-B2RT-40XMYC*, *RSRT-STOP-RSRT-6XV5-vMAT*/*Tdc2-GAL4*; *UAS-CD8-mCherry*, *UAS-B2*, *UAS-R*/+.

Figure 5S3. A-I) *yw*; *B2RT-STOP-B2RT-40XMYC*, *RSRT-STOP-RSRT-6XV5-vMAT*/+; *TRH-GAL4*, *UAS-CD8-mCherry*, *UAS-B2*, *UAS-R*.

Figure 5S4. A-I) *yw*; *B2RT-STOP-B2RT-40XMYC*, *RSRT-STOP-RSRT-6XV5-vMAT*/*Tdc2-GAL4*; *UAS-CD8-mCherry*, *UAS-B2*, *UAS-R*/+.

Figure 5S5. A-B) *yw*.

bioRxiv preprint doi: https://doi.org/10.1101/2025.07.23.666348; this version posted July 28, 2025. The copyright holder for this preprint (which was not certified by peer review) is the author/funder, who has granted bioRxiv a license to display the preprint in perpetuity. It is made available under aCC-BY 4.0 International license.

Figure 6S1. A-X) *yw*; *vGlut-40XMYC*, *6XV5-vMAT*/*vGlut-40XMYC*, *6XV5-vMAT*.

Figure 6S2. A-V) *yw*; *vGlut-40XMYC*, *6XV5-vMAT*/*vGlut-40XMYC*, *6XV5-vMAT*.

Figure 6S3. A-O) *yw*; *vGlut-40XMYC*, *6XV5-vMAT*/*IA2-GFP*.
Figure 6S4. A-O) *yw*; *vGlut-40XMYC*, *6XV5-vMAT*/*IA2-GFP*.

Figure 6S5. A, E, I) *yw*; *vGlut-40X5*/*vGlut-40XV5*. B, F, J) *yw*; *6XV5-vMAT*/*6XV5-vMAT*. C, G, K) *yw*; *7XMYC-vAChT*/*7XMYC-vAChT*. D, H, L) *yw*; *9XV5-vGAT*/*9XV5-vGAT*.

Figure 7S1. A) *yw*; *OAMB-GAL4*/*UAS-CD8-mCherry*. B) *yw*; OAα2R-GAL4/*UAS-CD8-mCherry*. C) *yw*; *Oct-TyrR-GAL4*/*UAS-CD8-mCherry*. D) *yw*; OAβ1R-GAL4/*UAS-CD8-mCherry*. E) *yw*; *OAβ2R-GAL4*/*UAS-CD8-mCherry*. F) *yw*; OAβ3R-GAL4/*UAS-CD8-mCherry*.

Figure 7S2. A) *yw*; *5-HT71A-GAL4*/+; UAS-CD8-mCherry/+. B) yw; *5-HT71B-GAL4*/+; *UAS-CD8-mCherry*/+. C) *yw*; *5-HT2A-GAL4*/*UAS-CD8-mCherry*. D) yw; *5-HT2B-GAL4*/*UAS-CD8-mCherry*. E) *yw*; *5-HT7-GAL4*/*UAS-CD8-mCherry*.

Figure 8S1. A-R) *yw*; *OAMB-10XV5*/*OAMB-10XV5*.

Figure 8S2. A-R) *yw*; *OAMB-10XV5*/*OAMB-10XV5*.

Figure 8S3. A-R) *yw*; *OAα2R-20XV5*/*OAα2R-20XV5*.

Figure 8S4. A-U) *yw*; *OAα2R-20XV5*/*OAα2R-20XV5*.

Figure 8S5. A-R) *yw*; *OAβ2R-40XV5*/*OAβ2R-40XV5*.

Figure 8S6. A-O) *yw*; *OAβ2R-40XV5*/*OAβ2R-40XV5*.

Figure 8S7. A-R) *yw*; *OAβ2R-40XV5*/*OAβ2R-40XV5*.

Figure 8S8. A-O) *yw*; *5-HT7-20XV5*/*5-HT7-20XV5*.

Figure 8S9. A-R) *yw*; *5-HT7-20XV5*/*5-HT7-20XV5*.

bioRxiv preprint doi: https://doi.org/10.1101/2025.07.23.666348; this version posted July 28, 2025. The copyright holder for this preprint (which was not certified by peer review) is the author/funder, who has granted bioRxiv a license to display the preprint in perpetuity. It is made available under aCC-BY 4.0 International license.

Figure 8S10. A-O) *yw*; *5-HT7-20XV5*/*5-HT7-20XV5*.

Figure 9S1. A-P) male *yw*; *UAS-H2A-GFP*/*TRH-AD*; *VT019028-GAL4-DBD*/+.

Figure 9S2. A-O) male *yw*; *UAS-H2A-GFP*/+; *VT019028-GAL4-DBD*/*AbdB-AD*.

Figure 9S3. A) male *yw*; *UAS-H2A-GFP*/*TRH-AD*; *VT019028-GAL4-DBD*/+. B) male *yw*; *UAS-H2A-GFP*/+; *VT019028-GAL4-DBD*/*AbdB-AD*.

Figure 10S1. A) *yw*; *ProtB-GFP*/+; B) *yw*; *ProtB-GFP*/+; *VT019028-GAL4-DBD*, *UAS-BONT-C*.

Figure 10S2. A) column 1-*yw*; *B3RT-vGlut-LexA*/*TRH-AD*, vGlut^{SS1}; *VT019028-GAL4-DBD*/*UAS-B3*. column 2-*yw*; *B3RT-vGlut-LexA*/*vGlut*^{SS1}; *TRH-GAL4-DBD*, *Tdc2-GAL4-DBD*/*AbdB-AD*, *UAS-B3*. column 3-*yw*; *B3RT-vGlut-LexA*/TRH-AD; *VT019028-GAL4-DBD*/*UAS-B3*. column 4-*yw*; *B3RT-vGlut-LexA*/*TRH-AD*, *vGlut*^{SS1}; *UAS-B3*/+. column 5-*yw*; *B3RT-vGlut-LexA*/*vGlut*^{SS1}; *VT019028-GAL4-DBD*/*UAS-B3*. B) column 1-*yw*; *B3RT-vGlut-LexA*/*TRH-AD*, *vGlut*^{SS1}; *VT019028-GAL4-DBD*/*UAS-B3*. column 2- yw; *B3RT-vGlut-LexA/TRH-AD; VT019028-GAL4-DBD/UAS-B3. column 3- yw; B3RT-vGlut-LexA*/*TRH-AD*, *vGlut*^{SS1}; *UAS-B3*/+. column 4-*yw*; *B3RT-vGlut-LexA*/*vGlut*^{SS1}; *VT019028-GAL4-DBD*/*UAS-B3*.

Figure 10S3. A-F) *yw*; *B3RT-vGlut-LexA*/*TRH-AD*, vGlut^{SS1}; *VT019028-GAL4-DBD*/*UAS-B3*, *LexAop-6XmCherry*.

Video 1. *yw*; *ProtB-GFP*/+.

Video 2. *yw*; *ProtB-GFP*/+; *VT019028-GAL4-DBD*, *UAS-BONT-C*.

bioRxiv preprint doi: https://doi.org/10.1101/2025.07.23.666348; this version posted July 28, 2025. The copyright holder for this preprint (which was not certified by peer review) is the author/funder, who has granted bioRxiv a license to display the preprint in perpetuity. It is made available under aCC-BY 4.0 International license.

Videos 3-9. *yw*: *MHC-GCAMP8m*.
